# Selective organ deposition of nanoparticles directed by metal–organic network coatings

**DOI:** 10.1038/s41467-026-77181-0

**Published:** 2026-09-02

**Authors:** Jingqu Chen, Yuang Gu, Zhaoran Wang, Tianzheng Wang, Zhixing Lin, Chang Liu, Omid Mazaheri, Shiyao Li, Soraia Fernandes, Ching-Seng Ang, Robert De Rose, Christina Cortez-Jugo, Frank Caruso

**Affiliations:** 1 Department of Chemical Engineering, The University of Melbourne, Parkville, Victoria, Australia; 2 Messenger Bio Pty Ltd, 700 Swanston St, Carlton, Victoria, Australia; 3 School of Science, RMIT University, Melbourne, Victoria, Australia; 4 Bio21 Institute, The University of Melbourne, Parkville, Victoria, Australia; 5 Department of Infectious Diseases, The University of Melbourne, Parkville, Victoria, Australia; 6 Present Address: Department of Chemical and Petroleum Engineering, Research and Innovation Center for Graphene and 2D Materials, Khalifa University, Abu Dhabi, United Arab Emirates

**Keywords:** Biomedical materials, Molecular self-assembly, Nanoparticles

## Abstract

Despite significant advances in nanoengineering, developing a universal nanoparticle engineering strategy that enables tailored nanoparticle organ deposition post intravenous administration remains challenging. Here, we report a simple and modular strategy mediated by metal-organic network coatings composed of polyethylene glycol (PEG), proteins, phenolic ligands, and metal ions. The versatile binding affinity of phenolic ligands facilitates the formation of coatings on diverse nanoparticle platforms, including quantum dots, lipid, polymeric, inorganic, and metal–organic nanoparticles. The organ deposition selectivity of these coated nanoparticles is readily directed towards the kidney, lung, heart, and brain by varying the coating composition. Mechanistic studies reveal that the combined effect of PEG, phenolic ligands, and metal ions on the surface properties governs the overall biodistribution of the coated nanoparticles, while the protein component contributes to the coating integrity. This work provides a generalizable approach for developing an organ-targeting nanotechnology, with the potential to advance nanoparticle-mediated treatments and theranostics.

## Abstract (web summary)

Developing a simple and broadly applicable strategy for tailoring the organ tropism of nanoparticles has been a long-standing challenge. Here, the authors address this challenge by introducing a metal–organic network coating approach that enables selective organ tropism across a wide range of established nanoparticle platforms by varying the coating composition.

## Introduction

Nanoparticle (NP)-enabled therapeutic delivery modalities have had a transformative impact on pharmaceutical industries and clinical practices^1–3^. For example, lipid nanoparticles (LNPs), the most clinically advanced gene delivery platform, have been applied in COVID-19 vaccines (e.g., Pfizer and Moderna vaccines) and in multiple clinical trials for vaccination^4^, gene editing^5^, protein replacement therapy^6^, and cancer immunotherapy^7^. Despite these advances, most nonviral NPs tend to be largely deposited within the liver and spleen post-intravenous (IV) administration^8,9^, significantly hampering their applications in other organ-specific diseases. There is thus ongoing research in developing viable approaches for organ-/tissue-selective therapeutic delivery. In many cases, selective organ/tissue targeting of NPs has been achieved through surface modification. This primarily includes the following strategies: incorporation of targeting moieties (e.g., monoclonal antibodies) that specifically bind to receptors or biomolecules overexpressed by cells from the target organs^10^; modulation of surface charge (e.g., incorporating additional building blocks with different charges)^11^; surface functionalization with polyethylene glycol (termed PEGylation) to reduce immune clearance and nonspecific uptake, thereby extending circulation and increasing the likelihood of reaching the target organ^12^; and biomimetic engineering (e.g., leveraging the intrinsic tissue-homing ability of immune cells and extracellular vesicles^13^)^10^. However, the NP platform deployed can vary significantly to accommodate specific applications, necessitating tailoring of the surface modification approach on a case-by-case basis^14,15^. Furthermore, the synthesis, conjugation, and purification of targeting ligands often require harsh and/or sophisticated procedures, while their organ-targeting efficiency remains limited^16,17^. Therefore, developing a simple and universal strategy that can afford specific organ tropism for a range of established NP delivery platforms could significantly advance the field of NP-mediated targeted therapeutic delivery.

Metal–organic network composites have attracted interest across diverse disciplines owing to their tunable composition and physicochemical properties^18^. Phenolics are naturally abundant organic ligands with metal-chelation capabilities and versatile binding affinity for diverse guest molecules (e.g., polymers, biomacromolecules, functional small molecules)^19^, affording the formation of biofunctional metal–phenolic networks (MPNs)^20^. Furthermore, the adhesive nature of phenolics enables MPN binding to diverse substrates, including NPs, making MPNs suitable as coatings that can integrate multiple components (>2) and functionalities within a single metal–organic matrix^21–23^. Manipulation of MPN components readily affords modulation of the surface and physicochemical properties of the resulting MPN coatings, thereby providing a potential pathway to control bio–nano interactions and biodistribution.

**Fig. 1:**
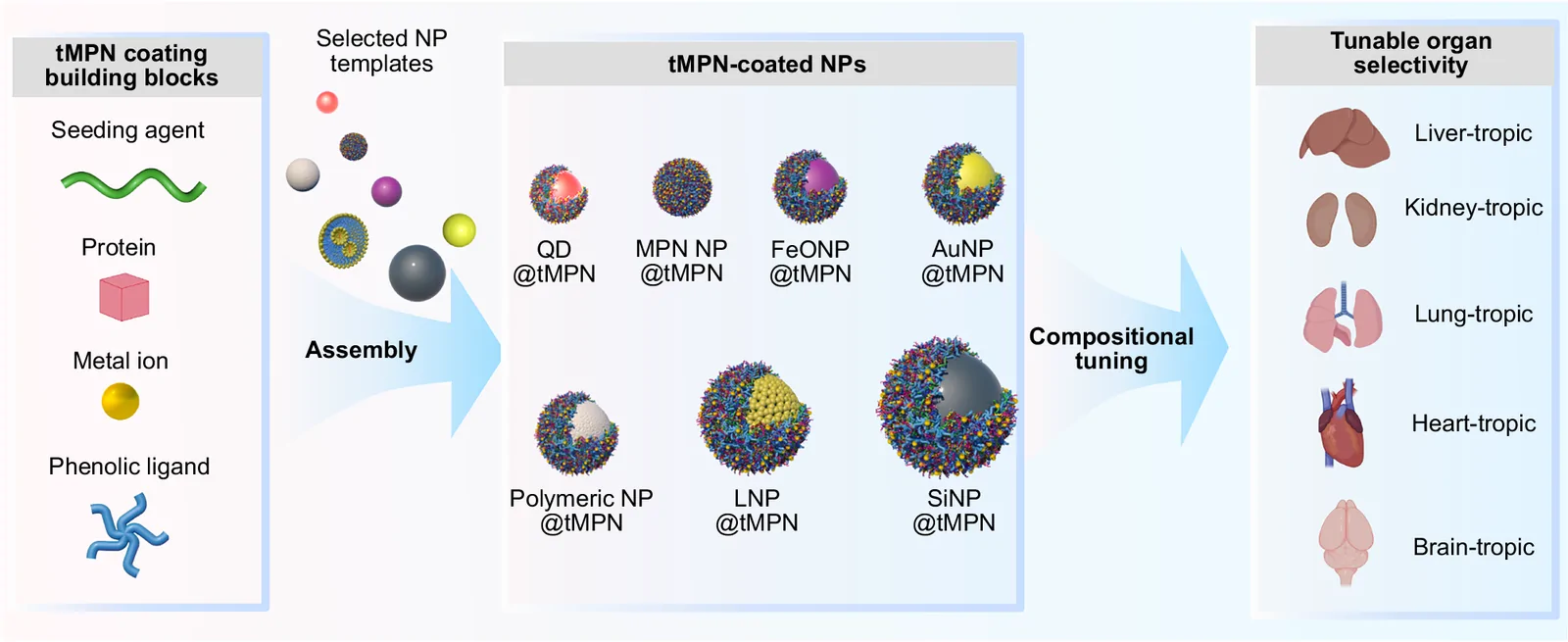
Schematic of the assembly of organ-targeting metal–phenolic network (tMPN) coatings that direct the organ deposition of established NP platforms of different size, charge, and composition. Organ deposition selectivity is enabled by modulating network composition (i.e., tMPN coating building blocks). QD quantum dot; MPN NP metal–phenolic network nanoparticle; FeONP paramagnetic Fe_3_O_4_ nanoparticle; AuNP gold nanoparticle; LNP lipid nanoparticle; SiNP silica nanoparticle. Figure 1 was partially created in BioRender. Chen, J. (2026) https://BioRender.com/rn4uct8.

In the present study, we report a generalizable metal–organic network coating strategy, which is termed organ-targeting metal–phenolic network (tMPN) coating, to tailor the organ tropism of diverse NPs (Fig. 1). The tMPN coating was typically composed of a seeding agent (i.e., PEG), a protein, a phenolic ligand, and a metal ion. The PEG, phenolic ligand, and metal ion collectively contributed toward governing the overall distribution of NPs in mice. The protein component showed minimal impact on biodistribution but was essential for the structural integrity of the tMPN coating. The seeding ability of PEG and the versatile binding affinity of phenolic ligands enabled coating of tMPNs on a range of established NP platforms, thereby modulating their surface properties. These platforms include LNPs, polymeric NPs, metal–organic NPs, gold NPs (AuNPs), silica NPs (SiNPs), quantum dots (QDs), and paramagnetic Fe_3_O_4_ NPs (FeONPs). Notably, simply by varying the metal ion type, phenolic ligand, and molecular weight (*M*_w_) of PEG, the organ tropism of these NP platforms was (re)-programmed/redirected to the kidney, lung, heart, or brain, achieving NP deposition beyond the liver and/or spleen after IV administration. Mechanistic studies suggest that this composition-mediated organ tropism is due to the combined effect of surface properties, physicochemical properties, and biomolecular corona formed on the NPs, with surface attributes playing a predominant role. The modularity and adaptability of the coating strategy, along with its selective organ deposition efficacy, not only advance the bioapplications of metal–organic systems but also open avenues to developing next-generation organ-targeting nanotechnology platforms for drug delivery, imaging, and theranostics.

## Results

### Effect of composition on organ deposition selectivity

**Fig. 2:**
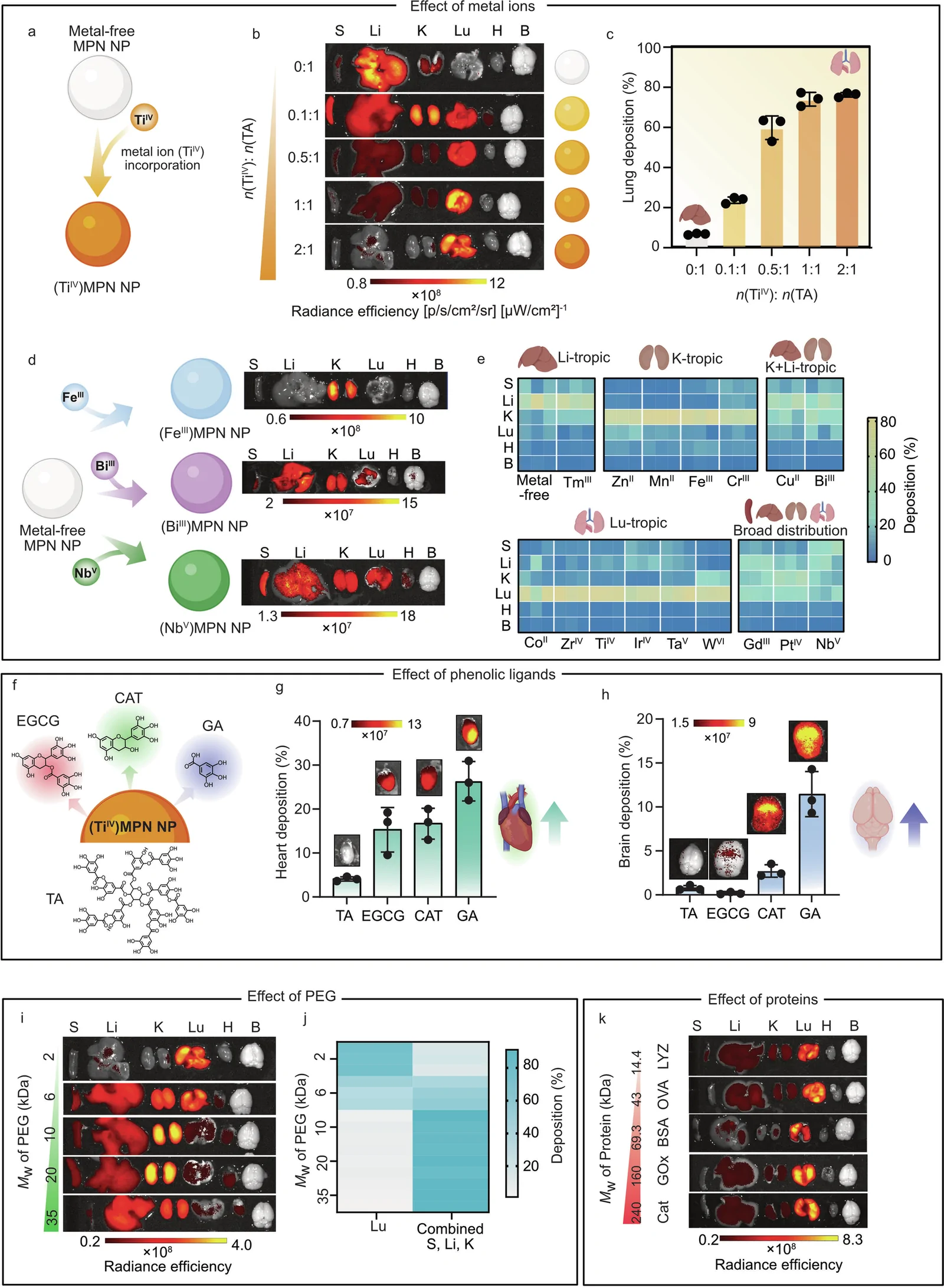
Effect of composition on organ deposition selectivity of metal–phenolic network nanoparticles (MPN NPs). **a** Schematic illustrating Ti^IV^ incorporation into metal-free MPN NPs, yielding Ti^IV^-containing MPN NPs (i.e., (Ti^IV^)MPN NPs). TA, 2 kDa PEG, and BSA were used as the phenolic ligand, PEG *M*_w_, and protein component, along with a metal ion-to-phenolic ligand molar ratio of 2:1, unless specified otherwise. **b**,** c** Effect of the amount of Ti^IV^ added to NPs on their biodistribution and lung targeting efficiency. Fluorescently labeled (Ti^IV^)MPN NPs were intravenously administered to C57BL/6 J mice. Both representative images (**b**) and quantitative data (**c**) were obtained by the In Vivo Imaging System (IVIS). Li, liver; K, kidney; Lu, lung; H, heart; S, spleen; B, brain. **d** Schematic of modulating the surface identity of MPN NPs by altering metal ions (Ti^IV^ is replaced by Fe^III^, Bi^III^, or Nb^V^ ions) and representative IVIS images showing organ selectivity of the resulting MPN NPs. **e** Heat map depicting organ deposition of MPN NPs composed of one of 16 different metal ions. **f–h** Effect of phenolic ligands on heart and brain deposition: schematic showing the substitution of phenolic ligands from TA to smaller-sized phenolics (**f**), quantitative and representative IVIS images of heart (**g**) and brain (**h**) deposition of (Ti^IV^)MPN NPs prepared using different phenolic ligands. **i**,** j** Effect of polyethylene glycol (PEG) on organ selectivity: representative IVIS images (**i**) and heat map (**j**) showing organ deposition of (Ti^IV^)MPN NPs prepared using different PEG *M*_w_. **k** Representative IVIS images of (Ti^IV^)MPN NP deposition upon varying proteins. The unit of radiance efficiency in all IVIS images is [p/s/cm^2^/sr][μW/cm^2^]^–1^. *n* = 3 mice per group; data are presented as mean ± sd. EGCG, (−)-epigallocatechin-3-*O*-gallate; CAT catechin; GA gallic acid; LYZ lysozyme; OVA ovalbumin; BSA bovine serum albumin; GOx glucose oxidase; Cat catalase. Illustrations in (**a–e, g, h**) were partially created in BioRender. Chen, J. (2026): **a** https://BioRender.com/ytlgd9u; **b** https://BioRender.com/43krkwc; **c** https://BioRender.com/28kunak; **d** https://BioRender.com/fsxdb96; **e** https://BioRender.com/ktnzmgf; **g**, **h** https://BioRender.com/dpq1w3f.

The surface composition of NPs plays an essential role in governing their transport in vivo^9^. Therefore, we first examined the effect of tMPN composition in directing organ tropism. To simplify the experimental setup, a metal-free MPN NP composed of PEG (2 kDa), bovine serum albumin (BSA), rhodamine 800 (Rh800; selected fluorophore), and tannic acid (TA) was assembled and employed as a particulate substrate. The surface of the metal-free MPN NPs was then modified by introducing different amounts of Ti^IV^, yielding Ti^IV^-incorporated MPN NPs (i.e., (Ti^IV^)MPN NPs) (Fig. 2a and Supplementary Table 1).

To determine the effect of metal ions on biodistribution, both the metal-free and (Ti^IV^)MPN NPs were intravenously injected into C57BL/6 J mice (NP dosage = 5 mg kg^–1^). Major organs, including the spleen, liver, kidney, lung, heart, and brain, were harvested and imaged for organ deposition by tracking Rh800 (Supplementary Fig. 1). The metal-free MPN NPs primarily accumulated in the liver (~60% of the accumulation across all harvested organs; Fig. 2b, c and Supplementary Fig. 2a). With the addition of Ti^IV^ at a Ti^IV^-to-TA molar ratio (*n*(Ti^IV^): *n*(TA)) of 0.1:1, the NPs were more broadly distributed across all organs. Increasing the molar ratio (i.e., the proportion of Ti^IV^) progressively increased lung deposition specificity, reaching ~80% at ratios of 1:1 and 2:1 (Fig. 2b, c and Supplementary Fig. 2b). This lung-selective deposition occurred within 4 h of injection and slowly decayed after 24 h (Supplementary Fig. 3). These results not only demonstrate biodistribution of MPN NPs mediated by metal ions but also indicate a likely threshold of metal ion proportion required for organ deposition selectivity.

To further evaluate the influence of metal ions on organ deposition selectivity, we varied the metal ion by incorporating Fe^III^, Bi^III^, or Nb^V^ at a metal ion-to-TA molar ratio of 2:1. As shown in Fig. 2d, introducing Fe^III^ into the metal-free MPN NPs shifted the organ tropism from the liver to the kidney. Incorporating Bi^III^ led to preferential deposition in metabolic organs (i.e., liver and kidney), whereas incorporating Nb^V^ resulted in broad distribution across multiple organs. We expanded our study to 16 different metal ions with valencies ranging from II to VI (Supplementary Fig. 4 and Supplementary Table 2), affording a variety of organ tropism (Fig. 2e and Supplementary Fig. 5): liver-tropic (e.g., 58% liver deposition enabled by Tm^III^), kidney-tropic (e.g., 68% enabled by Zn^II^), lung-tropic (e.g., 69% enabled by W^VI^), metabolic organ-tropic (e.g., Cu^II^), and broad distribution (e.g., Gd^III^). Furthermore, microscopy analysis on sectioned tissues indicated efficient cell association of the MPN NPs upon localization in the organs (Supplementary Figs. 6–8). Despite variations in the MPN NP size induced by altering metal ions (Supplementary Table 2), linear regression and correlation analyses revealed minimal correlation between NP size and organ tropism (Supplementary Fig. 9). We also note that 5 out of the 6 (Co^II^, Zr^IV^, Ti^IV^, Ir^IV^, Ta^V^, W^VI^) metal ions that enable lung-selective deposition (i.e., all >60%) possess valencies of IV or above (Fig. 2e and Supplementary Fig. 5), suggesting that incorporating metal ions with higher valencies may have a greater propensity to direct NPs to the lung.

Phenolic ligands coordinate multiple interactions among NP components^22^, and thus are anticipated to play a pivotal role in determining the properties and biodistribution of MPN NPs. To validate this hypothesis, additional phenolic ligands were examined (Fig. 2f). Notably, replacing TA (*M*_w_ = 1701.19 g mol^−1^) with small phenolic ligands enriched heart deposition of the MPN NPs (Fig. 2g and Supplementary Fig. 10). For example, substituting TA with catechin (i.e., CAT, *M*_w_ = 290.27 g mol^−1^) and gallic acid (i.e., GA, *M*_w_ = 170.12 g mol^−1^) led to ~four-fold and ~six-fold increases, respectively, in cardiac deposition (Fig. 2g). Cardiac enrichment, enabled by small phenolic ligands, was consistently observed regardless of the metal ion type (e.g., Tm^III^, Fe^III^, or Gd^III^) (Supplementary Fig. 11). This persistent enrichment further indicates the primary role of phenolic ligands in mediating cardiac deposition. Replacing TA with GA or CAT also enriched brain tropism by 15-fold or 3.5-fold, respectively (Fig. 2h). Small phenolic ligands are known to influence the permeability of brain endothelial cells^24^, which may provide a possible mechanism for the entry of MPN NPs into the brain. Overall, directing NPs to the heart and brain remains elusive in nanomedicine^25,26^. Leveraging tMPN compositions offers a potential pathway for more efficient delivery to these organs.

Next, we examined the role of the PEG component in modulating organ tropism. As shown in Fig. 2i,j, using 2 kDa PEG along with BSA, TA, and Ti^IV^ to assemble MPN NPs typically resulted in lung tropism. In contrast, using a higher PEG *M*_w_ gradually reduced the extent of lung tropism and resulted in a broader distribution pattern. When the PEG *M*_w_ increased to 10 kDa, <10% of MPN NPs were retained in the lung, and >80% were distributed across the spleen, liver, and kidney. As PEG is a low-fouling polymer and PEGylation can increase the stealthiness of NPs^27^, we investigated whether the broader organ distribution induced by a higher PEG *M*_w_ can be attributed to prolonged circulation time beyond 6 h. At 10 and 24 h postinjection (when most NPs are expected to exit circulation), the MPN NPs composed of a longer PEG showed a distribution pattern comparable to that observed at 6 h (Supplementary Fig. 12). These findings indicate that the *M*_w_ of PEG may influence the degree of organ deposition selectivity, with higher *M*_w_ PEGs more likely to attenuate organ selectivity and promote broader distribution of MPN NPs across organs.

Finally, the effect of the protein component was investigated by replacing BSA with alternative proteins having distinct *M*_w_s (14.4–240 kDa) and isoelectric points (4.3–10.7). Changing the protein component resulted in negligible changes in the lung-selective tropism of the (Ti^IV^)MPN NPs, suggesting the minimal role of protein components in determining the organ deposition of MPN NPs (Fig. 2k and Supplementary Fig. 13).

Our systematic investigation into the influence of composition on the organ deposition selectivity of MPN NPs reveals that metal ions, phenolic ligands, and PEG collectively play essential roles in determining the fate of NPs upon IV administration. PEG with low *M*_w_ (e.g., <6 kDa) may be required for achieving specific organ-selective distribution. Varying the metal ion type affords diverse organ-tropic distribution profiles, whereas varying the phenolic ligand type enables tuning the degree of tropism toward the heart and brain. Such a composition–organ selectivity correlation is expected to facilitate the rational design of organ-targeting coatings (i.e., tMPN coating) using these compositions to modify the surface of existing NP platforms and program their organ deposition selectivity.

### Redirecting organ tropism of MPN NPs with a tMPN coating

**Fig. 3:**
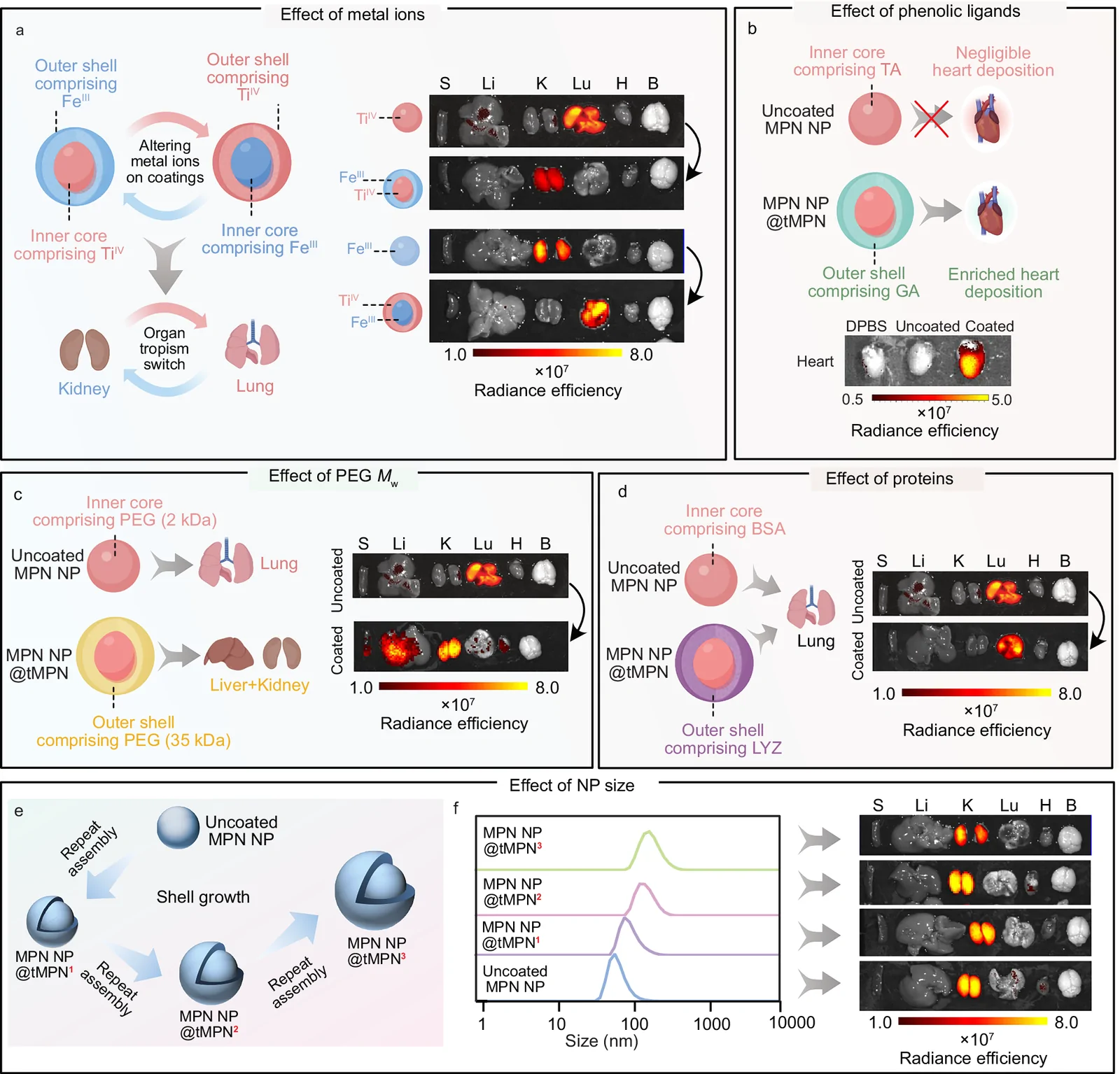
Redirecting organ tropism of metal–phenolic network nanoparticles (MPN NPs) with an organ-targeting metal–phenolic network (tMPN) coating. **a** Schematic and representative IVIS images showing organ tropism switch by introducing an outer shell with a different metal ion on MPN NPs. **b** Schematic and representative IVIS images showing heart enrichment of MPN NPs after introducing a gallic acid (GA)-containing outer tMPN shell. TA tannic acid; DPBS Dulbecco’s phosphate-buffered saline. **c** Schematic and representative IVIS images showing redirected biodistribution using a 35 kDa polyethylene glycol (PEG)-based outer shell. **d** Schematic and representative IVIS images distribution of MPN NPs after introducing a lysozyme (LYZ)-containing outer shell. BSA, bovine serum albumin. **e**, **f** Effect of NP size on biodistribution: schematic of MPN NPs with varied shell thickness prepared via repeated coating assembly (**e**) and corresponding NP size distribution (diameter, measured by dynamic light scattering) and biodistribution (imaged via IVIS) after 1, 2, and 3 shell growth iterations (**f**). The unit of radiance efficiency in all IVIS images is [p/s/cm^2^/sr][μW/cm^2^]^–1^. S spleen; Li liver; K kidney; Lu lung; H heart; B brain. Illustrations in (**a**–**d**) were partially created in BioRender. Chen, J. (2026): **a** https://BioRender.com/zaxmyq1; **b** https://BioRender.com/gx8x3x6; **c** https://BioRender.com/2eiyt8e; **d** https://BioRender.com/oziafru.

To demonstrate the proof-of-concept for redirecting organ tropism through a tMPN coating (metal ion-to-phenolic ligand molar ratio is 2:1 unless specified otherwise), we applied a kidney-tropic coating (tMPN^K^; composition: 2 kDa PEG, BSA, TA, Fe^III^) on a lung-tropic MPN NP substrate (i.e., (Ti^IV^)MPN NPs) (Fig. 3a and Supplementary Fig. 14). Fe^III^ in the outer shell directed the coated MPN NPs to the kidney despite the presence of lung-tropic metal ions (Ti^IV^) in the inner core (Fig. 3a). Similarly, coating a kidney-tropic inner core (containing Fe^III^) with a lung-tropic tMPN shell (containing Ti^IV^) (Supplementary Fig. 15) led to lung-tropic deposition (Fig. 3a).

In another example, uncoated (Ti^IV^)MPN NPs exhibited minimal accumulation in the heart, but coating of the NPs with heart-tropic tMPNs (composition: 2 kDa PEG, BSA, Ti^IV^, GA) resulted in NPs with pronounced cardiac deposition (Fig. 3b). This result is consistent with that shown in Fig. 2h, wherein substitution of TA in (Ti^IV^)MPN NPs with GA resulted in cardiac enrichment. In addition to phenolic ligands, the effects of PEG *M*_w_ and proteins on biodistribution were also adapted for tMPN coatings. Coating lung-selective (Ti^IV^)MPN NPs with tMPNs composed of 35 kDa PEG, BSA, TA, and Ti^IV^ altered biodistribution, leading to broad distribution across the spleen, liver, and kidney (Fig. 3c), whereas coating with tMPNs composed of lysozyme (LYZ), PEG (2 kDa), TA, and Ti^IV^ did not alter organ tropism (Fig. 3d).

The assembly of a tMPN coating on NP substrates is expected to increase the size of the NPs. To investigate the influence of size increases on the efficacy of organ tropism redirection, a kidney-tropic tMPN coating was formed on kidney-tropic (Fe^III^)MPN NP substrates (same composition). Coating was repeated accordingly to produce 1-layer (tMPN^1^), 2-layer (tMPN^2^), and 3-layer (tMPN^3^) coatings (Fig. 3e). Increasing the number of coating layers increased NP size, but kidney tropism was retained (Fig. 3f and Supplementary Fig. 16). Similarly, the application of 3 layers of lung-tropic or liver-tropic coatings resulted in organ tropism similar to that observed with the single-layer coatings (Supplementary Figs. 17 and 18). These results further support the correlation between organ tropism and tMPN composition and demonstrate the applicability of this relationship when tMPNs are engineered as coatings.

### Redirecting organ tropism of established NP platforms with a tMPN coating

**Fig. 4:**
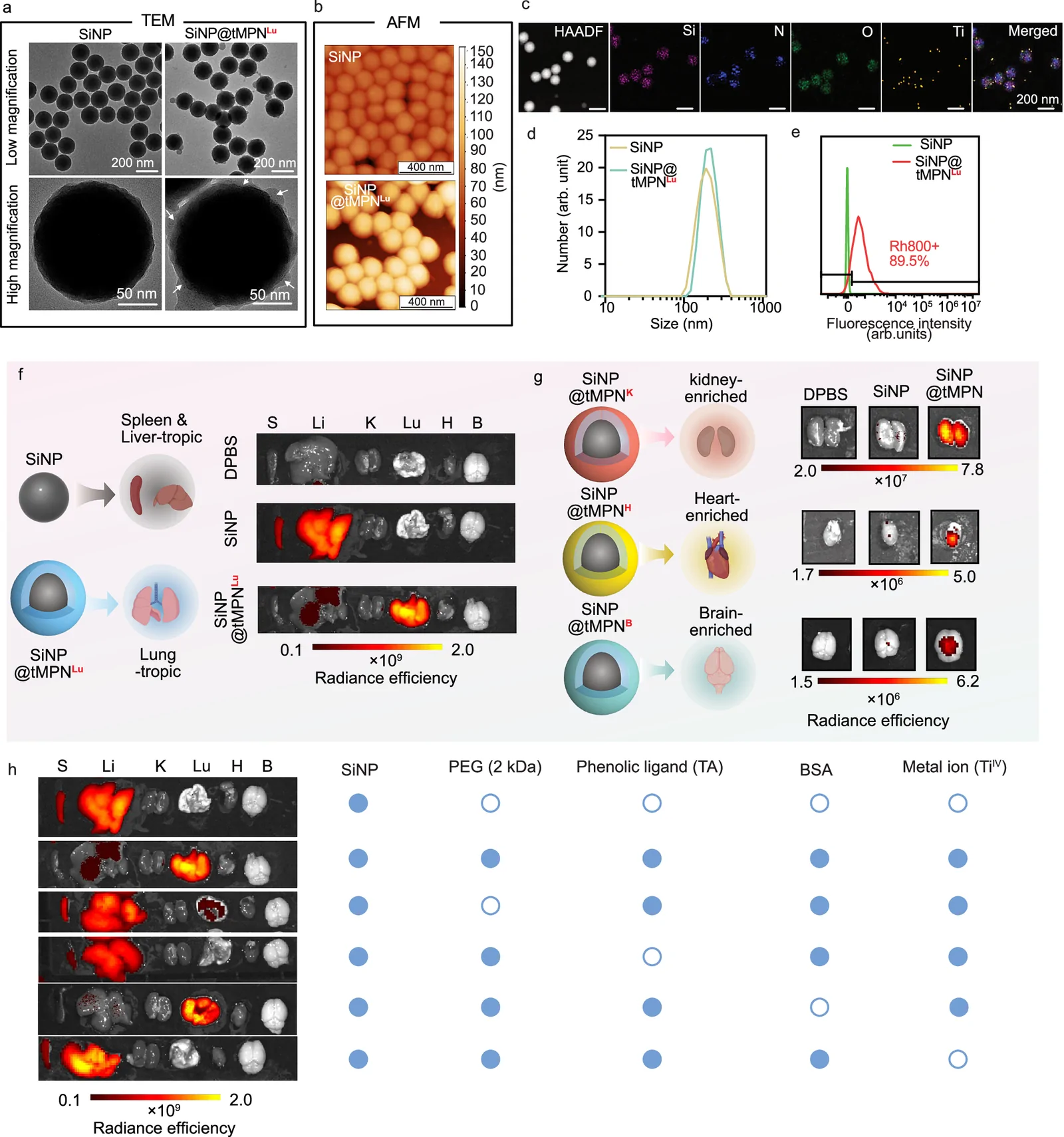
Redirecting organ tropism of silica nanoparticles (SiNPs) with an organ-targeting metal–phenolic network (tMPN) coating. a–e, Characterization of SiNP@tMPN^Lu^. Representative transmission electron microscopy (TEM) (**a**) and atomic force microscopy (AFM) (**b**) images of SiNPs before and after tMPN^Lu^ coating. White arrows in the high-magnification TEM image in (**a**) indicate representative surface changes upon tMPN coating. EDX mapping of SiNP@tMPN^Lu^ (**c**) and size distribution (diameter) of SiNPs before and after tMPN^Lu^ coating measured by dynamic light scattering (**d**). HAADF, high-angle annular dark field. For **a**–**c**, representative images from at least 3 independent experiments are shown. Quantification of the portion of SiNPs successfully coated with tMPNs (**e**). In coating quantification studies, SiNPs were not fluorescently labeled. Rh800 was selected as the fluorescent dye to probe tMPN^Lu^. **f**, **g** Schematic and representative IVIS images showing redirection of SiNP organ tropism using a tMPN coating. tMPN coatings applied on SiNPs are as follows: tMPN^Lu^ (composition: 2 kDa PEG, BSA, TA, Ti^IV^); tMPN^K^ (composition: 2 kDa PEG, BSA, TA, Cr^III^); tMPN^H^ (composition: 2 kDa PEG, BSA, EGCG, Ti^IV^); tMPN^B^ (composition: 2 kDa PEG, BSA, CAT, Ti^IV^). DPBS, Dulbecco’s phosphate-buffered saline. **h** Representative IVIS images and coating composition showing the role of each coating component in redirecting the biodistribution of SiNPs. The unit of radiance efficiency in all IVIS images is [p/s/cm^2^/sr][μW/cm^2^]^–1^. Solid blue circles show components used in coatings; empty circles show components not included in coatings. SiNPs were conjugated with Dylight 800 for biodistribution visualization post-IV injection. PEG polyethylene glycol; TA tannic acid; BSA bovine serum albumin; S spleen; Li liver; K kidney; Lu lung; H heart; B brain. Illustrations in (**f,** **g**) were partially created in BioRender. Chen, J. (2026) https://BioRender.com/smoiher.

In addition to MPN NP substrates, we next evaluated the versatility of this organ targeting/redirecting approach by coating tMPNs onto diverse established NP platforms. As a well-established and widely studied drug delivery platform, commercially available SiNPs (143 nm) were first examined. A lung-tropic tMPN coating (tMPN^Lu^; composition: 2 kDa PEG, BSA, TA, and Ti^IV^) was applied as a representative tMPN coating. The change in surface morphology observed upon tMPN^Lu^ coating in the transmission electron microscopy (TEM) images (Fig. 4a), the uniform increase in height profile across captured NPs (Fig. 4b), and the co-existence of Si and Ti determined by energy-dispersive X-ray spectroscopy (EDX) mapping (Fig. 4c) collectively confirm the tMPN^Lu^ coating of the SiNPs. Particle distribution analysis and atomic force microscopy (AFM) height profiling showed an approximately 30 nm increase in size and height post-coating (Fig. 4b, d, respectively), indicating a shell thickness of ~15 nm. Furthermore, flow cytometry analysis demonstrated that nearly 90% of SiNPs were coated with tMPN^Lu^ (Fig. 4e).

Next, the biodistribution of tMPN-coated SiNPs in mice was assessed by tracking the Dylight800-conjugated SiNPs under IVIS. As shown in Fig. 4f, SiNPs, which typically display splenic and hepatic tropism, were redirected to the lung upon coating with tMPN^Lu^. Likewise, when SiNPs were coated with the kidney-tropic (tMPN^K^), heart-enriching (tMPN^H^), or brain-enriching (tMPN^B^) tMPN, redirection of organ deposition to the kidney, heart, and brain, respectively, was observed (Fig. 4g).

To elucidate the role of individual coating components in redirecting organ tropism, control coatings were prepared by selectively omitting one of the four components (i.e., PEG, BSA, phenolic ligands, or metal ions) from the standard tMPN coating formulation (Fig. 4h). Using tMPN^Lu^ (composition: 2 kDa PEG, BSA, TA, Ti^IV^) as a representative coating, we noticed that the exclusion of any of the three components—2 kDa PEG, TA, or Ti^IV^—significantly impaired the redirection of the SiNPs to the lung. This finding suggests a combinatorial effect of PEG, phenolic ligands, and metal ions in modulating organ tropism. Notably, although the absence of BSA showed minimal effect on the efficacy of redirection, it was retained in all coating formulations because of its key role in maintaining coating stability.

**Fig. 5:**
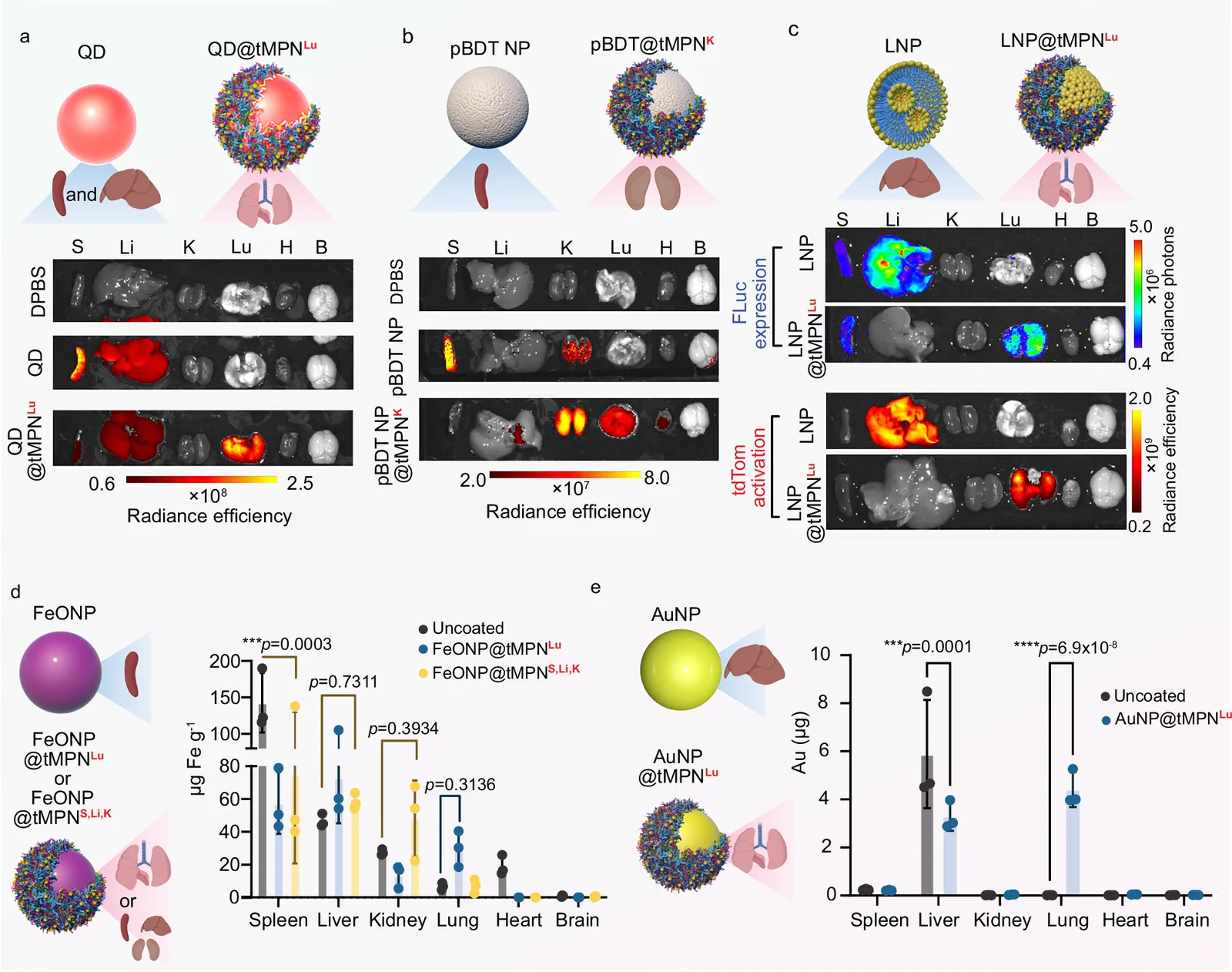
Redirecting organ tropism of established nanoparticle (NP) platforms with an organ-targeting metal–phenolic network (tMPN) coating. **a**–**c**, Representative IVIS images showing organ tropism redirection of quantum dots (QDs) (**a**), polymerized benzene-1,4-dithiol (pBDT) NPs (**b**), and SM-102 lipid nanoparticles (LNPs) (**c**). Images were captured 6 h post-IV administration in C57BL/6 J mice, while tdTomato (tdTom) expression imaging was conducted 3 days post-IV administration in B6.Cg-Gt(ROSA)26Sor^tm14(CAG-tdTomato)Hze^/J mice. Organ tropism redirection of LNPs (SM-102 LNPs) was indicated by tdTom and firefly luciferase (FLuc) expression in the lung. The dosages of Cre and FLuc mRNA were 5 µg per mouse. The unit of radiance efficiency is [p/s/cm^2^/sr][μW/cm^2^]^–1^, and the unit of radiance photons is [p/s/cm^2^/sr]. DPBS Dulbecco’s phosphate-buffered saline; S spleen; Li liver; K kidney; Lu lung; H heart; B brain. **d**, **e** ICP-MS elemental analysis of harvested organs showing organ tropism redirection of FeONPs (by tracking Fe) (**d**) and AuNPs (by tracking Au) (**e**). *n* = 3 mice per group; and data are presented as mean ± sd. Statistical significance was analyzed using two-way analysis of variance (ANOVA). In (**d**), although the individual values in the uncoated and FeONP@tMPN^S,Li,K^ groups (for liver deposition), and in the uncoated and FeONP@tMPN^Lu^ groups (for lung deposition) do not overlap, the statistical analysis does not indicate a significant difference, possibly because of the limited sample size. FeONP, paramagnetic Fe_3_O_4_ nanoparticle; AuNP, gold nanoparticle; Illustrations in (**a**–**e**) were partially created in BioRender. Chen, J. (2026) https://BioRender.com/vun7nce.

Five additional well-studied NP platforms—FeONPs, AuNPs, LNPs, QDs, and polymeric (polymerized benzene-1,4-dithiol or pBDT) NPs—were investigated and employed as particulate substrates for growth of organ-tropic tMPN coatings (Supplementary Figs. 19–23). As shown in Fig. 5a, b, the deposition of QDs (spleen- and liver-tropic) and pBDT NPs (spleen-tropic) was redirected primarily to the lung and kidney, respectively, via coating of the QDs and NPs with lung-tropic tMPNs (i.e., QD@tMPN^Lu^) and kidney-tropic tMPNs (pBDT NP@tMPN^K^).

LNPs are the most clinically advanced delivery platform and are considered the benchmark in nucleic acid delivery, yet LNPs typically accumulate mainly in the liver^8^. However, by coating LNPs with tMPN^Lu^ and tMPN^K^, liver-tropic LNPs (with SM102 as the ionizable lipid) were directed toward the lung and kidney, respectively (Fig. 5c and Supplementary Fig. 24). Importantly, the mRNA encapsulated within the LNPs remained functional after coating, as determined from the observed expression of firefly luciferase (FLuc) and Cre recombinase (Cre) in the organs. This work presents a strategy for organ-specific delivery of LNPs administered via IV.

Moreover, tMPN coating endowed inert NPs (e.g., FeONPs and AuNPs) with organ-selective capability. Inductively coupled plasma mass spectrometry (ICP-MS) analysis revealed that pristine FeONPs and AuNPs primarily localized in the spleen and liver, respectively. After coating the NPs with tMPN^Lu^ (i.e., FeONP@tMPN^Lu^ and AuNP@tMPN^Lu^; tMPN^Lu^ composition: 2 kDa PEG, BSA, TA, Ti^IV^), Fe and Au signals were enriched in the lung, consistent with their biodistribution observed on IVIS (Fig. 5d, e, Supplementary Figs. 25 and 26). Furthermore, using 20 kDa PEG in tMPN coatings for FeONPs (i.e., FeONP@tMPN^S,Li,K^; tMPN^S,Li,K^ composition: 20 kDa PEG, BSA, TA, Ti^IV^) resulted in broader distribution across the spleen, liver, and kidney (Fig. 5d, and Supplementary Fig. 25). This result is consistent with that observed for the MPN NPs, wherein replacing 2 kDa PEG in a lung-tropic MPN NPs with a higher *M*_w_ PEG (e.g., 20 kDa) reduced lung tropism while promoting broad distribution, with ~80% deposition across the spleen, liver, and kidney (Fig. 2i, j). These results indicate that the influence of PEG on organ tropism remains consistent when incorporated as a coating component, further highlighting the versatility and modularity of tMPNs. Collectively, our simple and versatile coating strategy using organ-tropic tMPNs is envisaged to offer opportunities for the application of existing NP platforms to advance targeted therapy and theranostics.

### Mechanistic insight underlying tMPN-mediated organ deposition selectivity

To determine the role of tMPN composition in directing organ tropism, different tMPN compositions were assembled into organ-tropic MPN NPs.

**Fig. 6:**
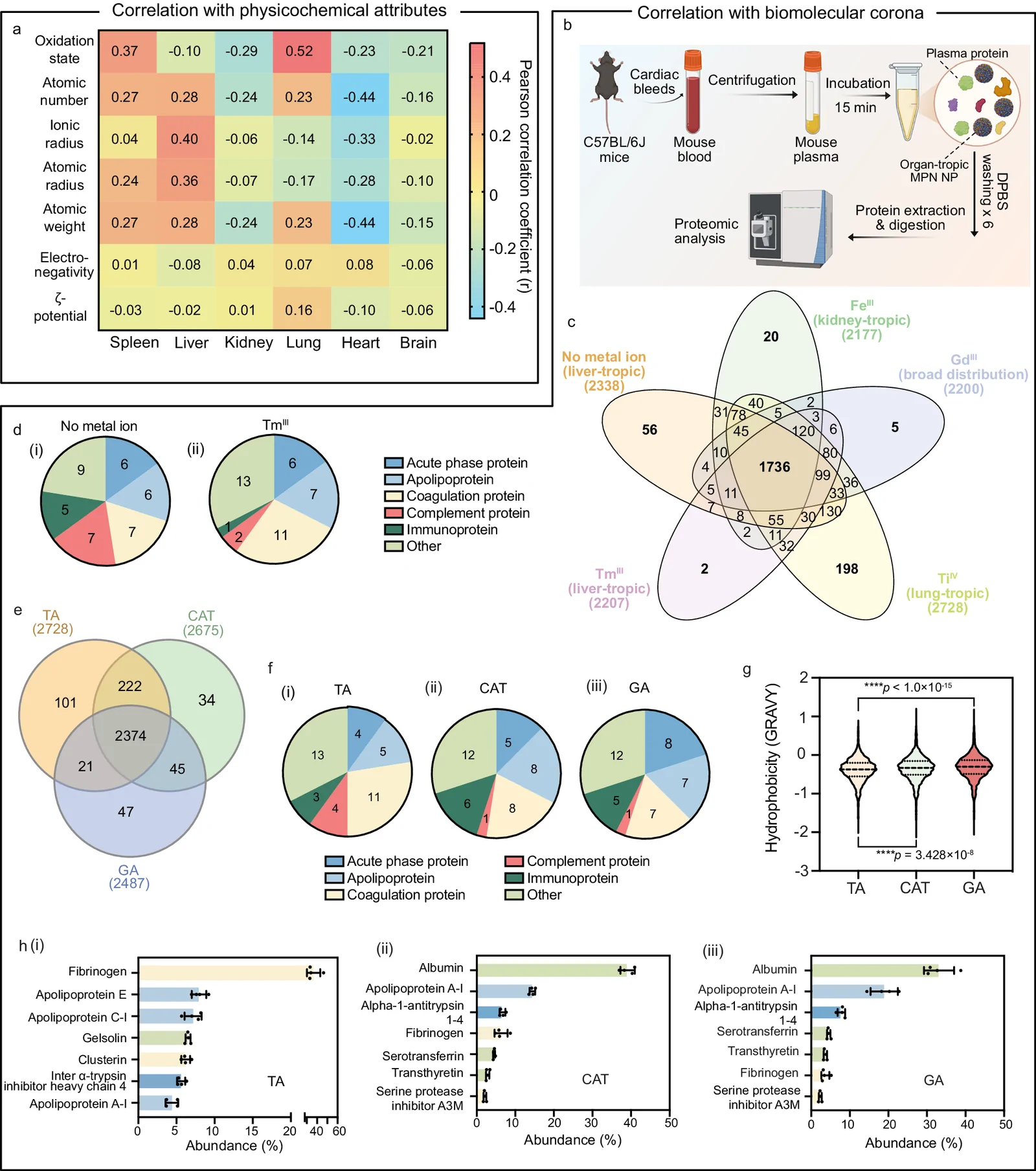
Mechanistic insights underlying selective organ tropism enabled by tMPN compositions. **a** Heat map of Pearson’s correlation coefficient corresponding to the correlation between each metal ion property and surface charge, with the extent of deposition in harvested organs. **b** Schematic depicting the collection of mouse plasma, formation, isolation, digestion, and analysis of protein corona formed on organ-tropic MPN NPs. The illustration in (**b**) was partially created in BioRender. Chen, J. (2026) https://BioRender.com/32kbwby. **c** Venn diagram of proteins identified in the corona formed on the MPN NPs composed of PEG (2 kDa), BSA, TA, and different metal ions. Proteins detected in at least one replicate were included in the analysis. **d**,** f** Average abundance of proteins with distinct biological functions in the protein coronas of NP formulations. Analysis was based on the top 40 most abundant plasma proteins identified in each selected MPN NPs. In (**d**), MPN NP composition: PEG (2 kDa), BSA, TA (for no metal ion) or PEG (2 kDa), BSA, TA, Tm^III^ (for Tm^III^). In **f**, MPN NP composition: PEG, BSA, Ti^IV^, and selected phenolic ligands (i.e., TA, CAT, or GA). **e** Venn diagram of proteins identified in the corona formed on the MPN NPs composed of PEG (2 kDa), BSA, Ti^IV^, and different phenolic ligands. **g** Violin plots showing the distribution of hydrophobicity of proteins associated with Ti^IV^-based MPN NPs prepared using different phenolic ligands. Dashed lines denote median values and interquartile ranges. Proteins detected in at least one replicate were included in the analysis. Statistical significance was analyzed using one-way ANOVA with the Kruskal–Wallis test. GRAVY, grand average of hydropathy. **h** Top seven most abundant plasma proteins that bind to different MPN NPs prepared using different phenolic ligands (TA (i), CAT (ii), and GA (iii)). Fibrinogen abundance is presented as a sum of alpha, beta, and gamma chain abundances. *n* = 4 independent replicates of a single NP formulation, each incubated with aliquots from the same pooled mouse plasma sample; and data are presented as mean ± sd. MPN NP composition: PEG, BSA, Ti^IV^, and selected phenolic ligands (i.e., TA, CAT, or GA). DPBS Dulbecco’s phosphate-buffered saline; TA tannic acid; CAT catechin, GA gallic acid.

The ζ-potential is an important characteristic of NPs that can influence their association with biological systems^28^. Therefore, we investigated the correlation between organ selectivity and ζ-potential. Furthermore, given the essential role of metal ion selection on the biodistribution of MPN NPs, we also investigated how metal properties (e.g., oxidation state, atomic number, ionic radius, atomic radius, atomic weight, and electronegativity) correlate with organ deposition selectivity^29^ (Supplementary Table 3), using linear regression and Pearson’s correlation coefficient (*r*) analyses. The organ-tropic TA-based MPN NPs in Fig. 2e were used in these analyses (Fig. 6a).

The MPN NPs were typically negatively charged regardless of the metal ions, and their ζ-potential displayed minimal linear correlation with tropism for all harvested organs in this work (Fig. 6a and Supplementary Fig. 27). Splenic deposition demonstrated some degree of positive linear correlation with the oxidation state of the metals (*r* = 0.37) (Fig. 6a and Supplementary Fig. 28). The atomic and ionic radius of the metal ions appeared to exert a positive linear correlation with liver deposition. Kidney deposition showed negligible linear correlation with all assessed parameters, but a slight positive correlation with NP size (*r* = 0.34) was observed (Fig. 6a, Supplementary Figs. 9, 27, and 28). It is speculated that ultrasmall (<10 nm) NPs may offer a pathway for kidney targeting as they tend to be cleared through renal filtration; however, larger NPs are likely to achieve kidney deposition by accumulating in the renal proximal tubule^30^. Notably, a stronger positive linear correlation between the oxidation state of the metals and lung deposition was observed (*r* = 0.52; Fig. 6a and Supplementary Fig. 28), which is consistent with our findings on metal ion-mediated biodistribution (Fig. 2e). Furthermore, heart deposition appeared to be negatively correlated with atomic number (*r* = –0.44) and atomic weight (*r* = –0.44), whereas brain deposition showed no distinct linear correlation with most assessed parameters (Fig. 6a, Supplementary Figs. 27 and 28).

The biomolecular corona, which defines the biological identity of NPs, has also been reported to influence biodistribution^9^. A biomolecular corona forms on the surface of NPs once they enter the biological milieu^31,32^, and its composition can be influenced by NP morphology, surface, and physicochemical properties^32^. TA-based metal-free NPs and a series of metal-containing MPN NPs with various organ tropisms were firstly employed to demonstrate how the metal ions influence biomolecular corona formation, and, consequently, biodistribution. The NPs were incubated with mouse plasma, and after six washes with Dulbecco’s phosphate-buffered saline (DPBS) to remove unbound proteins, the adsorbed plasma proteins were isolated and digested for proteomics analysis (Fig. 6b). The protein corona formed on NP surfaces is known to redistribute plasma protein representation prior to mass spectrometry analysis, facilitating the enrichment of lower-abundant protein species^33^. In our work, over 2000 protein species were identified across all tested TA-based MPN NPs, substantially exceeding those detected in the neat plasma (i.e., 1283 on average). The Venn diagram in Fig. 6c revealed that the examined TA-based MPN NPs shared 1736 proteins. The metal-free, TA/Fe^III^-based, TA/Gd^III^-based, TA/Ti^IV^-based, and TA/Tm^III^-based MPN NPs adsorbed 56, 20, 5, 198, and 2 unique proteins, respectively. Furthermore, Pearson correlation analysis demonstrated similarity (*r* > 0.92) in protein corona profiles among the metal ion-containing groups. In contrast, the metal-free group displayed relatively lower correlation values with each type of metal-containing group (i.e., *r* = 0.85–0.88), indicating that the inclusion of metal ions varies the protein corona composition (Supplementary Fig. 29).

Corona proteins with higher abundance have been shown to play a dominant role in determining the biodistribution of NPs^34^. Therefore, we analyzed the top 40 most abundant proteins for the selected TA-based MPN NPs (Supplementary Tables 4–9). These proteins were then categorized into acute phase proteins, apolipoproteins, coagulation proteins, complement proteins, immune proteins, and other proteins according to their biological functions^35^. Although the metal-free and TA/Tm^III^-based MPN NPs are both liver-tropic, they display distinct protein corona profiles (Fig. 6d). Specifically, in the metal-free MPN NPs, six categories of proteins were present at a comparable frequency. In comparison, for the TA/Tm^III^-based, as well as other examined metal ion-containing MPN NPs, the frequencies of complement proteins and immunoproteins were lower, whereas those of coagulation proteins and other proteins were higher (Fig. 6d and Supplementary Fig. 30a). We further categorized the top seven proteins based on their actual abundance (%) (Supplementary Fig. 30b and Supplementary Tables 4–9). Apolipoprotein A–I was highly enriched (17.9% abundance) on the metal-free MPN NPs. In contrast, the incorporation of and varying the metal ions led to minimal enrichment of specific proteins (Supplementary Fig. 30b). Likewise, the protein coronas exhibited highly comparable global physicochemical property distributions in terms of isoelectric point and hydrophobicity across these MPN NPs (Supplementary Fig. 31).

Although the overall protein corona composition appeared similar across TA-based MPN NPs, metal identity may still govern organ tropism through dynamic in vivo effects. Distinct metals can exhibit different coordination stability and protein exchange kinetics, leading to variations in partial dissociation, degradation behavior, particle size evolution, and aggregation under physiological conditions^36^. Such differences may alter surface exposure and interfacial properties over time, influencing vascular interactions^37^. Beyond the corona, metal-dependent modulation of hydration structure, local surface charge distribution, and hydrogen-bonding dynamics may influence interactions with organ-specific endothelial cells, which differ in shear stress, fenestration, and receptor expression. Coordinated metal centers could possibly engage in secondary interactions with phosphate-containing biomolecules^38^, the endothelial glycocalyx^39^, integrins^40^, or immune components^41^. Differential complement activation or macrophage recognition may also drive metal-dependent biodistribution despite similar bulk protein adsorption profiles.

We also investigated the effect of phenolic ligands on the protein corona using Ti^IV^ as the representative metal ion. MPN NPs prepared with different phenolic ligands shared 2374 proteins. The TA-based, CAT-based, and GA-based MPN NPs adsorbed 101, 34, and 47 unique proteins, respectively (Fig. 6e). Similar to the approach applied to MPN NPs prepared with different metal ions, we categorized the top 40 most abundant proteins into acute phase proteins, apolipoproteins, coagulation proteins, complement proteins, immune proteins, and other proteins. Compared to TA-based MPN NPs, the use of smaller phenolic ligands such as CAT or GA reduced the frequency of coagulant proteins and complement proteins while increasing the frequency of acute phase proteins and apolipoproteins (Fig. 6f). Notably, CAT-based and GA-based MPN NPs enriched more hydrophobic proteins compared to TA-based MPN NPs, as deduced by their higher grand average of hydropathy (GRAVY) values (Fig. 6g). To further analyze the protein corona profiles, we sorted the top seven proteins based on their actual abundance (%) (Fig. 6h, Supplementary Tables 7, 10 and 11). Fibrinogen was the most abundant protein adsorbed on the TA-based MPN NPs, with a typical relative abundance of 36.7% for TA/Ti^IV^-based MPN NPs. Replacing TA with CAT or GA reduced fibrinogen abundance to <10%, whereas albumin became the most predominant protein species (39.1% for CAT-based and 33.6% for GA-based MPN NPs). The relative abundance of apolipoprotein A-I increased to 14.4% and 18.7% for CAT-based and GA-based MPN NPs, respectively (Fig. 6h, Supplementary Tables 10 and 11). The enrichment of albumin and apolipoprotein A-I might prolong NP circulation in the bloodstream and facilitate interactions with low-density lipoprotein receptors, thereby enriching the heart and brain deposition^42^. These results support our IVIS observation for MPN NPs assembled using small ligands (Fig. 2f–h).

Collectively, our mechanistic studies show that selective organ deposition enabled by tMPN compositions may not be governed solely by NP physicochemical properties or protein corona composition. Instead, it is likely attributed to the combinatorial effect of NP composition and dynamic interactions with biological systems.

### In vivo safety and biocompatibility of tMPN compositions

Finally, the toxicity and biocompatibility of tMPN compositions were evaluated by formulating tMPN compositions into representative organ-tropic MPN NPs (i.e., liver-, kidney-, and lung-tropic, and broadly distributed) and intravenously injected into mice. In addition to the main organ(s) of NP deposition, two primary metabolic organs (i.e., liver and kidney) were collected for histological analysis and metal content analysis by ICP-MS. The ICP-MS data in Supplementary Fig. 32 revealed that, regardless of organ tropism, most of the metal ions introduced were retained in the liver and/or kidney 7 days postinjection and metabolized through these organs. Furthermore, hematoxylin and eosin staining was performed on sectioned tissues, with no significant inflammatory response, cellular swelling, or histological alterations observed in collected tissues (Supplementary Fig. 33), consistent with our previous toxicity findings on mRNA-loaded MPN NPs^21^. Moreover, these compositions induced negligible cytokine (interleukin-6 or tumor necrosis factor-α) release or weight loss in mice (Supplementary Fig. 34), collectively indicating high biocompatibility and minimal in vivo toxicity of the tMPN compositions examined.

## Discussion

We developed a highly versatile and biocompatible metal–organic network coating-mediated strategy to tailor organ deposition selectivity. The metal–organic network coating, termed tMPN, comprised metal ions, phenolic ligands, and PEG building blocks that collectively define NP composition. A fourth protein component, which plays a minimal role in organ deposition selectivity, serves as the structural component. The modularity of the tMPN coating and its diverse choice of building blocks enabled the generation of a toolbox for a broad spectrum of organ tropism, including kidney-tropic, lung-tropic, cardiac-enriched, and brain-enriched deposition. Notably, tMPN coatings showed broad applicability to existing NP platforms, including LNPs, polymeric NPs, metal–organic NPs, SiNPs, AuNPs, and FeONPs, redirecting them from sites of premature clearance (i.e., liver and/or spleen) to the target organs. Mechanistic studies revealed that organ deposition selectivity is not driven by a single factor but rather, a combined effect of surface composition, NP physicochemical properties, and the biomolecular corona, which influence the dynamic interactions of the NPs with the organ environment. However, further work is needed to understand the precise mechanism, which would involve a combination of proteomics, simulation, and artificial intelligence studies (e.g., to facilitate integrated analysis of proteomics and molecular dynamics simulation and to enable the development of predictive modeling to elucidate the interactions between endogenous proteins/enzymes and tMPN compositions). Overall, this tMPN coating-mediated strategy is expected to facilitate more predictable NP and therapeutic delivery to specific organs, while its modular nature may enable the secondary engineering of NPs for interactions with specific cell types. We envision that this strategy may underpin the rational design of organ-tropic nanotechnology platforms, paving the way for targeted delivery of therapeutic modalities and theranostics.

## Methods

### Ethics

This work was conducted in accordance with the Australian code for the care and use of animals for scientific purposes, and experiments were approved by The University of Melbourne Animal Ethics Committee (Ethics Nos. 10404, 31339, and 27608). In accordance with these approved ethics protocols, the in vitro biocompatibility of candidate nanoparticles was evaluated using an XTT cell viability assay in mammalian cells (MDA-MB-231 and HEK293T). Particle size and aggregation state (as indicated by both the size and polydispersity index) were characterized prior to administration, and only non-aggregated and non-cytotoxic formulations were subsequently evaluated in vivo.

### Materials

DPBS (catalog number #14190144), ultrapure DNase/RNase-free distilled water (#10977015), Dulbecco’s modified Eagle medium (#10569069), Alexa Fluor 594 NHS ester (#A20004), and 1,1′-dioctadecyl-3,3,3′,3′-tetramethylindotricarbocyanine iodide (DiR; #D12731) were purchased from Thermo Fisher Scientific. TA (ACS reagent grade, #403040), (−)-epigallocatechin-3-*O*-gallate (≥95%, #E4143), CAT (≥98%, #C1251), GA (certified reference material, *Trace*CERT^®^, #91215), CuCl_2_·2H_2_O (≥99%, #307483), CoCl_2_ (97%, #232696), TmCl_3_·6H_2_O (99.99%, #204668), CrCl_3_·6H_2_O (96%, #230723), titanium(IV) bis(ammonium lactato)dihydroxide (supplied as 50 wt.% solution in H_2_O, #388165), ZrCl_4_ (≥99.5%, #221880), FeCl_3_·6H_2_O (97–102%, #236489), ZnCl_2_ (≥98%, #208086), MnCl_2_·4H_2_O (≥99%, #244589), BiCl_3_ (99.99%, #254142), GdCl_3_·6H_2_O (99%, #G7532), IrCl_4_ (technical grade, #334480), PtCl_4_ (≥99.99%, #379840), NbCl_5_ (99%, #215791), TaCl_5_ (99.8%, #218634), WCl_6_ (≥99.9%, #241911), linear PEG (1.1 kDa, #17738; 2 kDa, #202509; 3.35 kDa, BioUltra grade, #88276; 6 kDa, #81260; 10 kDa, #30902820; 20 kDa, #81275; and 35 kDa, #81310), Rh800 (#83701), 3-(*N*-morpholino)propanesulfonic acid (MOPS; ≥99.5%, #M1254), BSA (≥98.0%, #05470), ovalbumin (≥98.0%, #A5503), LYZ (≥90.0%, #L6876), glucose oxidase (100,000–250,000 units g^−1^ solid, #G7141), and catalase (2000–5000 units mg^−1^ protein, #C9322) were purchased from Sigma-Aldrich (USA). mRNA encoding FLuc (#FLUC1000P) and Cre recombinase (#CRE1000P) were purchased from Messenger Bio Pty. Ltd. (Australia). 8-[(2-Hydroxyethyl)[6-oxo-6-(undecyloxy)hexyl]amino]-octanoic acid, 1-octylnonyl ester (SM-102; 98%, #C1042) was purchased from APExBIO (TX, USA). 1,2-Distearoyl-*sn*-glycero-3-phosphocholine (DSPC; ≥99%, #850365), cholesterol (≥98–99%, #700100), and 1,2-dimyristoyl-*rac*-glycero-3-methoxypolyethylene glycol-2000 (DMG-PEG-2000; ≥99%, #880151) were purchased from Avanti Polar Lipids (AL, USA). All chemicals were used as received without further purification. All aqueous solutions were filtered with 220 nm diameter membranes before the in vivo experiments.

### Assembly of fluorescent TA-based organ-tropic MPN NPs

TA/Ti^IV^-based organ-tropic MPN NPs (i.e., (Ti^IV^)MPN NPs) were employed as the lead NP formulation in this work. In a typical synthesis, PEG (200 μL, 2 kDa, 20 mg mL^−1^), BSA (100 μL, 10 mg mL^−1^), Rh800 (200 μL, 23.8 μg mL^−1^), TA (200 μL, 5 mg mL^−1^), and Ti^IV^ (i.e., titanium(IV) bis(ammonium lactato)dihydroxide; 69.1 μL, 5 mg mL^−1^) were successively added to a vial containing Milli-Q water (2 mL) and DPBS (500 μL). The metal ion-to-TA molar ratio was 2:1. The NPs were obtained by stirring the mixture for 10 min at room temperature, followed by purification via centrifugation (8000 *g*, 10 min). The resultant MPN NPs were dispersed in Milli-Q water for future use.

BSA could be replaced with other proteins, including LYZ (100 μL, 2 mg mL^−1^), ovalbumin (100 μL, 6.8 mg mL^−1^), glucose oxidase (50 μL, 1.28 mg mL^−1^ in PBS), or catalase (50 μL, 2 mg mL^−1^ in PBS).

Fluorescent TA-based MPN NPs comprising different metal ions were also prepared. For instance, the assembly of TA-based MPN NPs comprising Ir^IV^ or W^VI^ followed the same procedure as the assembly of (Ti^IV^)MPN NPs, except that Ti^IV^ was replaced with Ir^IV^ (79 μL, 6.8 mg mL^−1^) or W^VI^ (232.5 μL, 1 mg mL^−1^ in methanol).

For NP assembly with other metal ions, PEG (200 μL, 2 kDa, 20 mg mL^−1^), BSA (100 μL, 10 mg mL^−1^), Rh800 (200 μL, 23.8 μg mL^−1^), selected metal ions, and TA (200 μL, 5 mg mL^−1^) were sequentially introduced into a vial containing Milli-Q water (2 mL). The metal ion-to-TA molar ratio was 2:1 and the following metal ions were used in this work: FeCl_3_·6H_2_O (64 μL, 5 mg mL^−1^), BiCl_3_ (74 µL, 5 mg mL^−1^ in methanol), ﻿CoCl_2_ (30.8 μL, 5 mg mL^−1^), CuCl_2_·2H_2_O (40 μL, 5 mg mL^−1^), CrCl_3_·6H_2_O (63 μL, 5 mg mL^−1^), TmCl_3_·6H_2_O (63.4 μL, 7.1 mg mL^−1^), ZrCl_4_ (55 μL, 5 mg mL^−1^), ZnCl_2_ (32 μL, 5 mg mL^−1^), MnCl_2_ (46.2 μL, 5 mg mL^−1^), NbCl_5_ (45 μL, 7 mg mL^−1^ in ethanol), and TaCl_5_ (26.5 μL, 16 mg mL^−1^ in ethanol).

### Assembly of tMPN coating on MPN NPs (MPN NP@tMPN)

For the fabrication of MPN NPs with a core–shell structure, the core MPN NP containing metal ion 1 was first formed as described above. An aliquot (1 mL) of the core NP suspension, which represents the seed, was added to Milli-Q water (1 mL) under stirring. Then, linear PEG (2 kDa), BSA, metal ion 2, and TA were added, followed by stirring for 10 min. This resulted in MPN NP@tMPN comprising two types of metal ions (e.g., Ti^IV^ in the core and Fe^III^ in the shell) that were then washed by centrifugation (8000 *g*, 10 min) and dispersed in Milli-Q water or buffer for future use.

### Assembly of tMPN coating on SiNPs (SiNP@tMPN)

To fabricate SiNP@tMPN, commercially available aminated SiNPs (800 μL; microParticles, Germany, #AR-1164) were first washed twice via centrifugation (4000 *g*, 5 min) and dispersed in Milli-Q water (800 μL). The SiNPs were then fluorescently labeled by incubating them overnight with Dylight 800 NHS ester (40 μL, 1 mg mL^−1^, #46421) under ambient conditions. The labeled SiNPs were purified by centrifugation (4000 *g*, 5 min) thrice to remove excess fluorescence dye and subsequently dispersed in Milli-Q water (800 μL).

Then, fluorescent SiNPs (60 μL), PEG (2 kDa, 100 μL, 20 mg mL^−1^), BSA (50 μL, 10 mg mL^−1^), CrCl_3_·6H_2_O (32 μL, 5 mg mL^−1^; to assemble SiNP@tMPN^K^ for redirection to the kidney) or Ti^IV^ (34 μL, 5 mg mL^−1^; to assemble SiNP@tMPN^Lu^ for redirection to the lung), and TA (100 μL, 5 mg mL^−1^) were successively added to a vial containing Milli-Q water (2 mL). After 10 min of stirring, the assembled NPs were stabilized by adding MOPS buffer (20 µL, 100 mM). The resultant SiNP@tMPN were purified by centrifugation (4000 *g*, 5 min) and dispersed in MOPS buffer (2 mM, pH 7). The percentage of SiNPs coated with tMPNs was quantified on a CytoFLEX Nano flow cytometer (Beckman Coulter, USA). The gating strategy is presented in Supplementary Fig. 35. Flow cytometry analysis was performed using FlowJo V10.

### Assembly of tMPN coating on LNPs (LNP@tMPN)

An ethanol phase containing SM-102, DSPC, cholesterol, DMG-PEG-2000, and DiR at a molar ratio of 50:10:38.5:1.5:0.2 was prepared. An aqueous phase was prepared by suspending Cre or FLuc mRNA in 25 mM citrate buffer (pH 4). The two phases were then combined at a nitrogen-to-phosphate ratio of 6:1 and an aqueous phase-to-ethanol phase ratio of 3:1 with a flow rate of 10 mL min^–1^ using a NanoAssemblr Ignite (Vancouver, Canada). SM-102 LNPs (LNPs simply) were then dialyzed overnight in DPBS using a 12 kDa cutoff dialysis tube (Sigma-Aldrich, USA, #PURX12015) and stored at 4 °C.

To fabricate LNP@tMPN, LNPs (with 5 μg mRNA), PEG (40 μL, 20 mg mL^−1^), BSA (12.5 μL, 10 mg mL^−1^), CrCl_3_·6H_2_O (12.6 μL, 5 mg mL^−1^; to assemble LNP@tMPN^K^ for redirection to the kidney) or CoCl_2_ (6 μL, 5 mg mL^−1^; to assemble LNP@tMPN^Lu^ for redirection to the lung), and TA (40 μL, 5 mg mL^−1^) were successively added to a vial containing Milli-Q water (1.8 mL). After 10 min of stirring, the assembled NPs were stabilized by adding MOPS buffer (20 μL, 100 mM). The resultant LNP@tMPN was purified via centrifugation (4000 *g*, 5 min) using Amicon ultra centrifugal filters (100 kDa cutoff, Sigma-Aldrich, USA, #UFC210024) and finally dispersed in MOPS buffer (200 μL, 2 mM). The percentage of LNPs coated with tMPNs was quantified by flow cytometry. The gating strategy is presented in Supplementary Fig. 36, and the coated LNPs were imaged via lattice structured illumination microscopy (lattice SIM).

### Characterization

The ζ-potential, number size distribution, and polydispersity index of the NPs were measured in solution (either Milli-Q water or buffer solution) via dynamic light scattering analysis on a Zetasizer Nano-ZS instrument (Malvern Instruments, UK). Super-resolution microscopy images were obtained on a lattice SIM instrument (Zeiss Elyra with Lattice SIM^2^ processing function). The morphology was analyzed by TEM on an FEI Tecnai Spirit instrument at an operating voltage of 120 kV. The elemental profiles of NPs were obtained via EDX mapping on an FEI Tecnai TF20 instrument at an operating voltage of 200 kV. UV–Vis absorption spectra were recorded on a Specord 250 Plus spectrophotometer (Analytik Jena AG, Germany).

### Animals and housing conditions

The 6–12-week-old C57BL/6 J mice and B6.Cg-Gt(ROSA)26Sortm14(CAG-tdTomato)Hze/J mice (also known as Ai14 mice)^43^ with mixed genders used for the experimental studies were sourced from the Bioresources Facility at the Peter Doherty Institute (Melbourne, Australia), Walter and Eliza Hall Institute of Medical Research (Melbourne, Australia), and Australian BioResources (New South Wales, Australia) and housed on a 12 h light/dark cycle at an ambient temperature of 19–20 °C and relative humidity of 40–70%, with ad libitum access to food and water.

### Biodistribution in mice

Briefly, the fluorescent NPs (i.e., MPN NPs or NP@tMPN) were intravenously administered into healthy C57BL/6 J mice (mixed sex; 6–12 weeks old) at a dosage of 5 mg kg^−1^ (according to the dosage guideline of approved animal Ethics Nos. 10404, 31339, and 27608) via the lateral tail vein. The same volume of DPBS was administered as the negative control. Mice were euthanized after 6 h (unless otherwise specified), while major organs, including the spleen, liver, kidney, lung, heart, and brain, were harvested and imaged using IVIS (V4.4, PerkinElmer).

For redirection studies using Cre^mRNA^-loaded LNPs, Ai14 mice were used to enable visualization of tdTom expression. LNP@tMPN were administered intravenously at an mRNA dose of 0.25 mg kg⁻¹. Mice were euthanized 3 days post-IV administration, and the abovementioned major organs were collected for IVIS imaging.

The average radiance efficiency was measured using the Living Image Software (V4.4 PerkinElmer). The normalized radiance efficiency and relative radiance efficiency (%) of each organ were calculated using the following equations^23,34^:

1 $${I}_{{{{\rm{normalized}}}}}=\frac{{I}_{{{{\rm{NP}}}}}}{{I}_{{{{\rm{DPBS}}}}}}$$

2 $${I}_{{{{\rm{relative}}}}}=\frac{{I}_{{{{\rm{NP}}}}}-{I}_{{{{\rm{DPBS}}}}}}{\sum ({I}_{{{{\rm{NP}}}}}-{I}_{{{{\rm{DPBS}}}}})}\times 100\%$$

where *I*_normalized_ is the normalized average radiance efficiency of the selected organ, *I*_NP_ is the average radiance efficiency of the organ in the NP-treated mice, *I*_DPBS_ is the average radiance efficiency of the organ in the DPBS-treated mice, and *I*_relative_ is the percentage of the average radiance efficiency of a specific organ relative to the total average radiance efficiency of all harvested organs.

For biodistribution studies conducted herein, sex-disaggregated analysis was not performed because sex was not the primary biological variable under investigation, and the study was not designed to assess sex-based differences. No sex-related data were collected.

### Elemental analysis via ICP-MS

MPN NPs, FeONP@tMPN, or AuNP@tMPN were intravenously administered into C57BL/6 J mice (mixed gender; 6–12 weeks old) at a dosage of 5 mg kg^–1^. Mice were euthanized at designated time points, and major organs, including the spleen, liver, kidney, lung, heart, and brain, were harvested.

The fresh organs were weighed and digested in 70% HNO_3_ at 80 °C for 30 min while the representative organ-tropic MPN NPs were digested by incubating with 70% HNO_3_ overnight at room temperature. After digestion, both organ and selected NP samples were diluted with Milli-Q water to achieve a final HNO_3_ concentration of 2%.

Metal ion standards were prepared using ICP single- or multi-element standard solutions (Sigma-Aldrich, USA, #67363, #01496, #51844, #110714). Metal content in the digested samples and the standard was quantified on a NexION 2000 ICP-MS instrument (PerkinElmer, USA).

### Isolation of plasma proteins adsorbed onto organ-tropic MPN NPs

Blood from untreated healthy C57BL/6 J mice (mixed gender; 6–12 weeks old) was collected via cardiac puncture, and mouse plasma was subsequently obtained through centrifugation (900 *g*, 15 min). Representative organ-targeted TA-based MPN NPs (i.e., metal-free, TA/Fe^III^-based, TA/Tm^III^-based, TA/Ti^IV^-based, and TA/Gd^III^-based) and Ti^IV^-based MPN NPs (i.e., TA/Ti^IV^-based, CAT/Ti^IV^-based, and GA/Ti^IV^-based) were synthesized according to the abovementioned protocol. Equal masses of NP suspension (200 μg per replicate) were mixed with mouse plasma (50 μL) at 2:1 (v/v) and incubated for 15 min to allow the formation of a biomolecular corona on the NP surface. After incubation, the corona-coated MPN NP suspensions were dispersed in 500 μL DPBS and centrifuged (9000 *g*, 10 min) six times, and pellets were redispersed in DPBS (30 μL).

### Preparation of plasma proteins for MS analysis

For protein extraction and digestion, proteins from neat plasma and an aliquot (30 μL) of NP suspensions were digested into peptides using S-Trap micro spin columns (ProtiFi, USA #CO2-micro), according to the manufacturer’s protocol.

Briefly, lysis buffer (12 μL, 10% sodium dodecyl sulfate, 100 mM tetraethylammonium bromide (TEAB, pH 8.5) was added to each sample (30 μL), followed by sonication to solubilize proteins. Then, the reducing agent (2 μL, 120 mM tris(2-carboxyethyl)phosphine in water) was added, and the samples were incubated at 55 °C for 15 min. Subsequently, the alkylating agent (2 μL, 500 mM iodoacetamide in 50 mM TEAB) was added and incubated at room temperature for 10 min. Next, acidifier (5 μL, 27.5% phosphoric acid in water) was added, followed by vortexing. To each sample, binding/wash buffer (150 μL, 100 mM TEAB in 90% methanol, pH 7.55) was added and mixed thoroughly. The sample was transferred to an S-Trap micro column placed in a 1.7 mL receiver tube and centrifuged at 4000 *g* for 30 s to trap proteins. The S-Trap was washed thrice with the binding/wash buffer (150 μL) and centrifuged at 4000 *g* for 30 s each time. After the final wash, the column was centrifuged at 4000 *g* for 1 min to completely remove residual buffer. For digestion, the digestion buffer (20 μL, 50 mM TEAB with 2 μg sequencing grade modified trypsin) was added to each column and incubated overnight at 37 °C. To elute peptides, three sequential elutions were performed using the following buffers (40 μL): (1) 50 mM TEAB in water, (2) 0.2% formic acid in water, and (3) 50% acetonitrile in water. After each addition, columns were centrifuged at 4000 *g* for 1 min. The eluates were pooled and freeze-dried. The freeze-dried peptides were resuspended in 2% (v/v) acetonitrile, 0.05% trifluoroacetic acid, before analysis by MS.

Liquid chromatography–data-independent acquisition (DIA)–MS was conducted on an Orbitrap Ascend mass spectrometer (Thermo Fisher Scientific) equipped with a nanoflow reversed phase high-performance liquid chromatography system (Ultimate 3000 RSLC, Dionex) fitted with an Acclaim PepMap nano-trap column (Dionex C18, 100 Å, 75 µm × 2 cm) and an Acclaim PepMap RSLC analytical column (Dionex C18, 100 Å, 75 µm × 50 cm). The tryptic peptides were injected into the enrichment column at an isocratic flow of 5 µL min^–1^ of 2% v/v CH_3_CN containing 0.1% v/v formic acid for 5 min, applied before the enrichment column was switched in-line with the analytical column. The eluents were 5% dimethyl sulfoxide in 0.1% v/v formic acid (solvent A) and 5% dimethyl sulfoxide in 100% v/v CH_3_CN and 0.1% v/v formic acid (solvent B). The flow gradient was (i) 0–6 min at 3% B, (ii) 6–7 min at 3–4% B, (iii) 7–82 min at 4–25% B, (iv) 82–86 min at 25–40% B, (v) 86–87 min at 40–80% B, (vi) 87–90 min to maintain at 80% B, (vii) 90–90.5 min at 80–3% B and equilibrated at 3% B for 10 min before the next sample injection. For DIA experiments, full MS resolutions were set to 120,000 at *m*/*z* 200 and scanning from 350–1400 *m*/*z* in profile mode. Full MS automatic gain control target was 250% with an injection time of 50 ms. The automatic gain control target value for fragment spectra was set at 2000%, 50 windows of 13.7 Da were used with an overlap of 1 Da, and the resolution was set to 30,000, maximum IT to 55 ms, and normalized collision energy at 30%. All data were acquired in centroid mode using positive polarity.

### Database searching and protein identification

DIA data were analyzed using the direct DIA analysis workflow with default settings on the Spectronaut software (v. 19.4.241104.62635) and against the Mus Musculus database (Uniprot, downloaded Mar 2025). Trypsin specificity was set to two missed cleavages. Carbamidomethyl was defined as a fixed modification, and acetylation (protein N-term) and oxidation (Met) as variable modifications. Results were filtered at a protein and peptide-spectrum match false discovery rate of 1%. Precursor filtering using the *q*-value and quantification performed on the MS2 level, and cross-run normalization strategy set to off. Quantitative data (PG.results) and iBAQ values (PG.IBAQ) were exported for statistical analyses, including correlation plots using the Perseus tool (version 2.0.3.1). iBAQ determines the abundance of a protein by dividing the total precursor intensities by the number of theoretically observable peptides of the protein. The top proteins detected on coronas were determined by percentage intensity based on iBAQ values^44^. Three-way and five-way Venn diagrams were generated using the InteractiVenn tool^45^.

### Statistics and reproducibility

Statistical analyses were performed using GraphPad PrismV.9 and V.10 (GraphPad Software). Linear regressions were performed using MATLAB R2024b (MathWorks). Statistical analysis details are provided in the caption of each figure. Unpaired *t*-tests were applied to compare two datasets. One-way and two-way ANOVA tests were used for more than two datasets, unless stated otherwise. Statistical analysis was performed with a 95% confidence interval. Data are expressed as mean values ± sd. ****p   <   0.0001; ***p   <   0.001; **p   <   0.01; *p   <   0.05.

### Reporting summary

Further information on research design is available in the Nature Portfolio Reporting Summary linked to this article.

## Supplementary information

Supplementary Information

Reporting Summary

Transparent Peer Review file

## Source data

Source data

## Data Availability

All relevant data supporting the findings of this study are available within the paper and Supplementary Information. Source Data are provided in this paper. The mass spectrometry proteomics data generated in this study have been deposited in the ProteomeXchange Consortium via the PRIDE^46^ partner repository under accession code PXD077631. All data underlying this study are available from the corresponding author upon request. Source data are provided with this paper.
