# Supplementary material for: Selective organ deposition of nanoparticles directed by metal–organic network coatings
Source: Nat Commun. 2026 Sep 2;17:10440. doi: 10.1038/s41467-026-77181-0 (PMC13631259; doi:10.1038/s41467-026-77181-0)
Supplement: Supplementary file 1 [file 41467_2026_77181_MOESM1_ESM.pdf]

Supplementary Information

Selective organ deposition of nanoparticles directed by metal–organic network coatings

*Jingqu Chen[1,†], Yuang Gu[1,†], Zhaoran Wang[1], Tianzheng Wang[1], Zhixing Lin[1,ξ], Chang Liu[1,2], Omid Mazaheri[1], Shiyao Li[1,3], Soraia Fernandes[1], Ching-Seng Ang[4], Robert De Rose[1,5], Christina Cortez-Jugo[1] & Frank Caruso[1]**

[1] Department of Chemical Engineering, The University of Melbourne, Parkville, Victoria 3010, Australia

[2] Messenger Bio Pty Ltd., 700 Swanston St, Carlton, Victoria 3053, Australia

[3] School of Science, RMIT University, Melbourne, Victoria 3000, Australia

[4] Bio21 Institute, The University of Melbourne, Parkville, Victoria 3010, Australia

[5] Department of Infectious Diseases, The University of Melbourne, Parkville, Victoria 3010, Australia

[ξ] Present address: Department of Chemical and Petroleum Engineering, Research and Innovation Center for Graphene and 2D Materials, Khalifa University, Abu Dhabi 127788, United Arab Emirates

[†] These authors contributed equally to this work

*email: fcaruso@unimelb.edu.au

Additional Supplementary Methods

Assembly of EGCG-based MPN NPs comprising different metal ions

Metal-containing EGCG-based MPN NPs were assembled as per the procedure used for the preparation of TA-based MPN NPs. Briefly, PEG (2 kDa, 20 mg mL^{-1}) and BSA (100 µL, 10 mg mL^{-1}), selected metal ions, and EGCG were successively introduced into a vial containing Milli-Q water (2 mL). The amount of metal ion and EGCG for each representative metal ion were as follows:

For Fe^{III} or Tm^{III}: $FeCl_3·6H_2O$ (99 µL, 5 mg mL^{-1}) or $TmCl_3·6H_2O$ (96 µL, 7.1 mg mL^{-1}) and EGCG (180 µL, 5 mg mL^{-1}). For Gd^{III}: $GdCl_3·6H_2O$ (132 µL, 5 mg mL^{-1}) and EGCG (270 µL, 5 mg mL^{-1}).

The suspensions were then stirred for 10 min, purified via centrifugation (8000 *g*, 10 min), and finally dispersed in Milli-Q water or buffer solutions for future use.

Assembly of a tMPN coating on AuNPs (AuNP@tMPN)

An aliquot (100 µL) of a AuNP suspension (50 nm, Sigma-Aldrich, USA, #742007) was briefly vortexed and dispersed in a vial containing Milli-Q water (2 mL) and DPBS (500 µL). Then, PEG (2 kDa, 200 µL, 20 mg mL^{-1}), BSA (100 µL, 10 mg mL^{-1}), Rh800 (100 µL, 23.8 µg mL^{-1}), TA (200 µL, 5 mg mL^{-1}), and Ti^{IV} (69.1 µL, 5 mg mL^{-1}) were sequentially added to the vial. The suspension was then stirred for 10 min, purified via centrifugation (8000 *g*, 10 min), and finally dispersed in Milli-Q water or buffer solutions for future use.

Assembly of a tMPN coating on pBDT NPs (pBDT NP@tMPN)

Fluorescence-labeled pBDT NPs were first prepared according to previous protocols[1,2]. Briefly, sodium dodecyl sulfate (SDS; 0.5% v/v; 750 µL), bicine buffer (10 mM, pH 8.5, 9 mL), BDT (2 mg mL^{-1} in dimethylformamide, 1.44 mL), TA (4 mg mL^{-1}, 1.5 mL), and Rh800 (2 mg mL^{-1} in dimethylformamide, 40 µL) were sequentially added to a vial containing Milli-Q water (3 mL). The mixture was stirred for 10 h to allow the formation of fluorescent pBDT NPs. Excess materials were removed by centrifugation (18000 *g*, 10 min) and the resulting fluorescent pBDT NPs were dispersed in Milli-Q water (500 µL) for future use.

In a vial containing Milli-Q water (2 mL), the pBDT NP suspension (60 µL), PEG (100 µL, 20 mg mL^{-1}), BSA (50 µL, 10 mg mL^{-1}), $CrCl_3·6H_2O$ (32 µL, 5 mg mL^{-1}), and TA (100 µL, 5 mg mL^{-1}) were successively introduced and the mixture was subsequently stirred for 10 min to enable the assembly of pBDT $NP@tMPN^{K}$ (kidney-tropic tMPN-coated NPs). Centrifugation (8000 *g*, 10 min) was conducted for purification and the resulting pBDT $NP@tMPN^{K}$ were dispersed in MOPS buffer (2 mM, pH 7). The percentage of pBDT NPs coated with tMPNs was quantified on a CytoFLEX Nano flow cytometer (Beckman Coulter, USA). The gating strategy used is shown in Supplementary Fig. 37.

Assembly of a tMPN coating on QDs (QD@tMPN)

In a vial containing Milli-Q water (1 mL) and DPBS (0.25 mL), commercial QDs (Sigma-Aldrich, #900226, 25 µL), PEG (50 µL, 20 mg mL^{-1}), BSA (25 µL, 10 mg mL^{-1}), TA (50 µL,

5 mg mL^{-1}), and Ti^{IV} (17 µL, 5 mg mL^{-1}) were successively introduced and the mixture was subsequently stirred for 10 min to enable the assembly of QD@tMPN^{Lu}. Centrifugation (8000 *g*, 10 min) was performed for purification and the resulting QD@tMPN^{Lu} were dispersed in Milli-Q water for future use. The structure of QD@tMPN^{Lu} was imaged via lattice SIM, with Rhodamine 110 (Rh110, Sigma-Aldrich, #432202, 50 µL, 3.5 µg mL^{-1}) incorporated to label the tMPN coating.

Assembly of a tMPN coating on FeONPs (FeONP@tMPN)

Fe_3O_4 NPs (i.e., FeONPs) were synthesized by hydrolysis of an aqueous solution containing iron salts and a base at room temperature (22 °C) in ambient atmosphere[3]. Ferrous sulfate ($FeSO_4$, Sigma-Aldrich, #1270355, 0.017 mol mL^{-1}) and ferric sulfate ($Fe_2(SO_4)_3$, Sigma-Aldrich, #307718, 0.033 mol mL^{-1}) were mixed and the pH of the solution was adjusted to 12 using 1,6-hexanediamine (Sigma-Aldrich, #1270355, H11696), resulting in immediate precipitation. After vigorous stirring for 24 h, the precipitation was separated using an external neodymium magnet and washed with ultra-pure water to remove excess amine molecules. FeONPs were finally collected as a black powder after drying at room temperature.

In a vial containing Milli-Q water (2 mL) and DPBS (0.5 mL), the prepared FeONPs (40 µL, 1 mg mL^{-1} in water), PEG (200 µL, 20 mg mL^{-1}), BSA (100 µL, 10 mg mL^{-1}), Rh800 (100 µL, 23.8 µg mL^{-1}), TA (200 µL, 5 mg mL^{-1}), and Ti^{IV} (69.1 µL, 5 mg mL^{-1}) were successively introduced and the mixture was subsequently stirred for 10 min to enable the assembly of FeONP@tMPN^{Lu}. For the preparation of FeONP@tMPN^{S,Li,K}, 20 kDa PEG was used, whereas 2 kDa PEG was used for the preparation of FeONP@tMPN^{Lu}. Centrifugation (8000 *g*, 10 min) was performed for purification and the resulting FeONP@tMPN were dispersed in Milli-Q water for future use. TEM imaging of FeONPs before and after coating was performed as detailed in the *Characterization* section. Mass quantification of Fe in harvested organs post-IV injection was performed as detailed in the *Elemental analysis via ICP-MS* section.

Sectioned organ analysis

Representative organ-tropic TA-based MPN NPs (i.e., metal free, TA/Fe^{III}-based, TA/Tm^{III}-based, TA/Ir^{IV}-based, and TA/Gd^{III}-based) were selected for microscopy analysis. The target organs were harvested 6 h post-IV administration. These organs were fixed with 4% paraformaldehyde (Thermo Fisher Scientific, #J19943.K2) (4 °C, overnight), dehydrated with 15 and 30% sucrose solutions (prepared with 1× PBS) until the organ sedimented, followed by embedding in frozen optimal cutting temperature medium. The entire tissue block was frozen in dry ice and cut into sections using a CM1950 cryostat (Leica Biosystems, Australia) with a thickness of 10–12 µm. The sections were placed on glass slides with 4',6-diamidino-2-phenylindole (DAPI) fluoromount (to stain the cell nucleus) for subsequent confocal microscopy analysis. Images were processed using ImageJ software (V2.3.0).

In vivo safety and biocompatibility of tMPN compositions

Representative organ-targeted TA-based organ-tropic MPN NPs (i.e., metal free, TA/Fe^{III}-based, TA/Tm^{III}-based, TA/Ti^{IV}-based, and TA/Gd^{III}-based) were intravenously administrated into C57BL/6J mice. Two primary metabolic organs (i.e., liver and kidney) and targeted organs

for each type of representative MPN NPs were harvested 7 days postinjection. These harvested organs were fixed with 4% paraformaldehyde (4 °C, overnight), dehydrated with 15 and 30% sucrose solutions until the organs settled down, followed by embedding in a frozen optimal cutting temperature medium. The entire tissue block was frozen in dry ice and cut into sections using the same cryostat with a thickness of 10–12 µm. The sections were placed on glass slides for subsequent hematoxylin and eosin staining (following manufacturer's protocol).

For cytokine assays, mouse blood was collected 7 days postinjection via cardiac puncture, and plasma was obtained via centrifugation (900 g, 15 min). The levels of interleukin-6 (IL-6) and tumor necrosis factor-α (TNF-α) were quantified using enzyme-linked immunosorbent assay kits (Invitrogen, USA).

Atomic force microscopy imaging and height analysis

Imaging was performed using a Cypher ES (Oxford Instruments/Asylum Research, Santa Barbara, USA) atomic force microscope, employing an air probe holder in AC mode at 25°C. Bare silica particles deposited on a silica wafer were imaged using an AC50TS (Oxford Instruments, UK) probe with nominal free air resonance $f = 1500$ kHz and nominal spring constant $k = 85$ N m^{-1}. Colloidal crystals of bare silica NPs allowed sizing by Fourier analysis and autocorrelation function of cross-sections in Gwyddion. SiNP@$tMPN^{Lu}$ were imaged using HQ:NSC 15/AlBs (nominal $f = 325$ kHz, nominal $k = 40$ n m^{-1}). Data processing was done using the Asylum Research native software (AR 18.45.52) and open-source Gwyddion 2.67.

Supplementary Figures

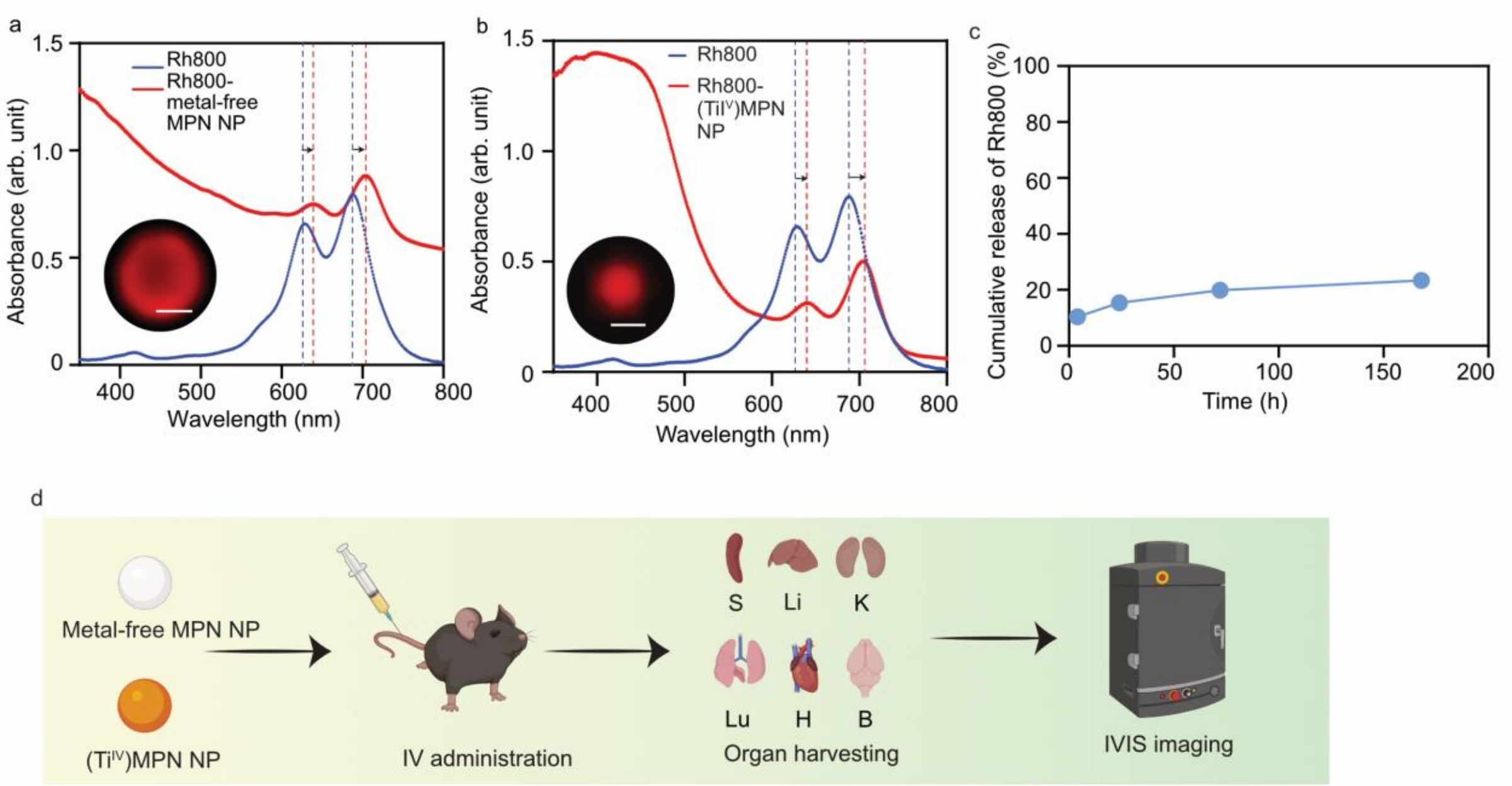

Supplementary Fig. 1 | Characterization and biodistribution experimental procedure of Rh800-labeled MPN NPs. a,b, UV–vis spectra of Rh800, and Rh800-labeled metal-free (**a**) and Ti^{IV}-incorporated ((Ti^{IV})MPN NPs) MPN NPs (**b**). Insets are lattice-SIM images of Rh800-labeled metal-free NPs (**a**) and (Ti^{IV})MPN NPs (**b**). Scale bars are 50 nm. **c,** Time-dependent release of Rh800 from (Ti^{IV})MPN NPs upon incubation with mouse serum. $n = 3$ independent experiments, and the data are presented as mean ± SD. Error bars are too small to be observed in the diagram. **d**, Schematic illustrating the evaluation of organ deposition of fluorescent MPN NPs injected intravenously in C57BL/6J mice. Li, liver; K, kidney; Lu, lung; H, heart; S, spleen; B, brain. Scheme in (**d**) was partially created in BioRender. Chen, J. (2026) https://BioRender.com/6nz4w6y. NP composition: 2 kDa PEG, BSA, TA, with or without Ti^{IV}; Ti^{IV}-to-TA molar ratio is 2:1.

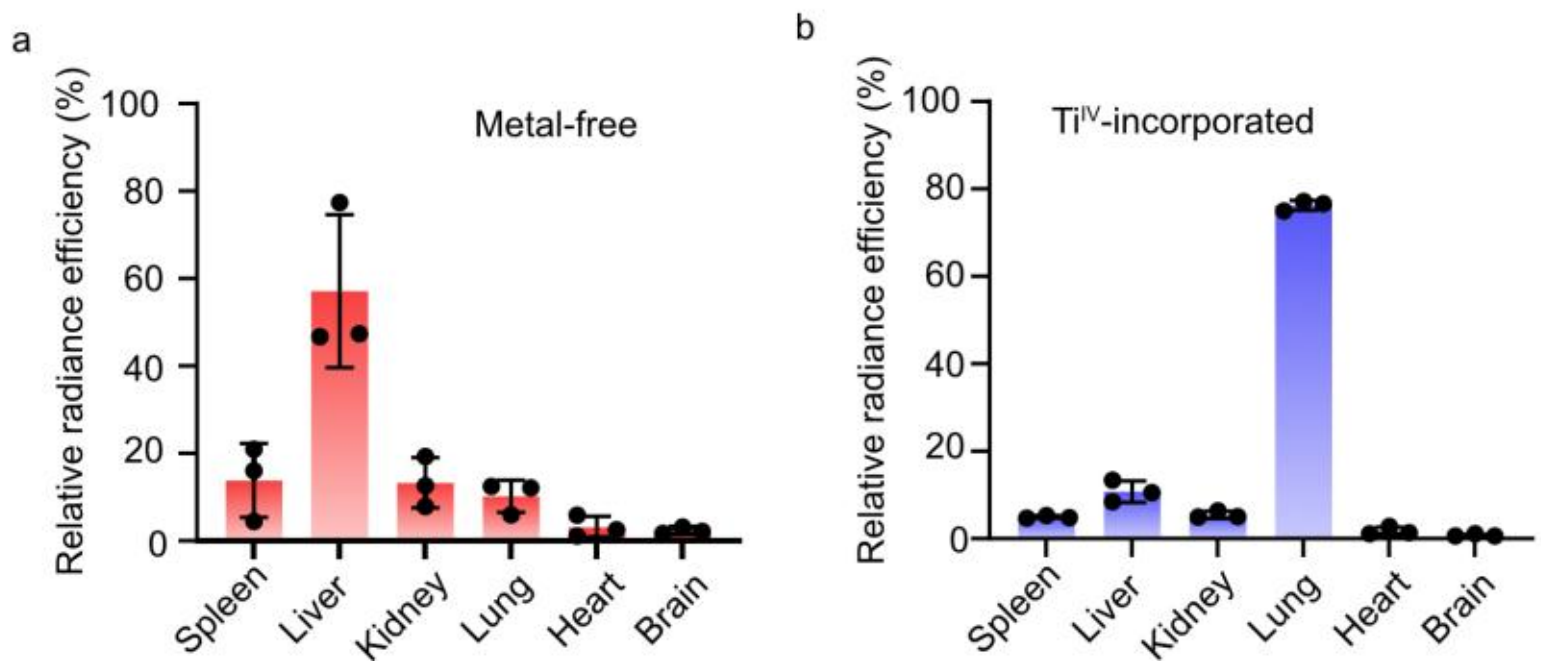

Supplementary Fig. 2 | Effect of Ti^{IV} incorporation on the biodistribution of MPN NPs. a,b, Biodistribution of MPN NPs before (**a**) and after (**b**) Ti^{IV} incorporation. The quantitative data were obtained using IVIS 6 h postinjection with a NP dosage of 5 mg kg^{-1}. $n = 3$ mice per group; the data are presented as mean ± SD. NP composition: 2 kDa PEG, BSA, and TA, with or without Ti^{IV}; Ti^{IV}-to-TA molar ratio is 2:1.

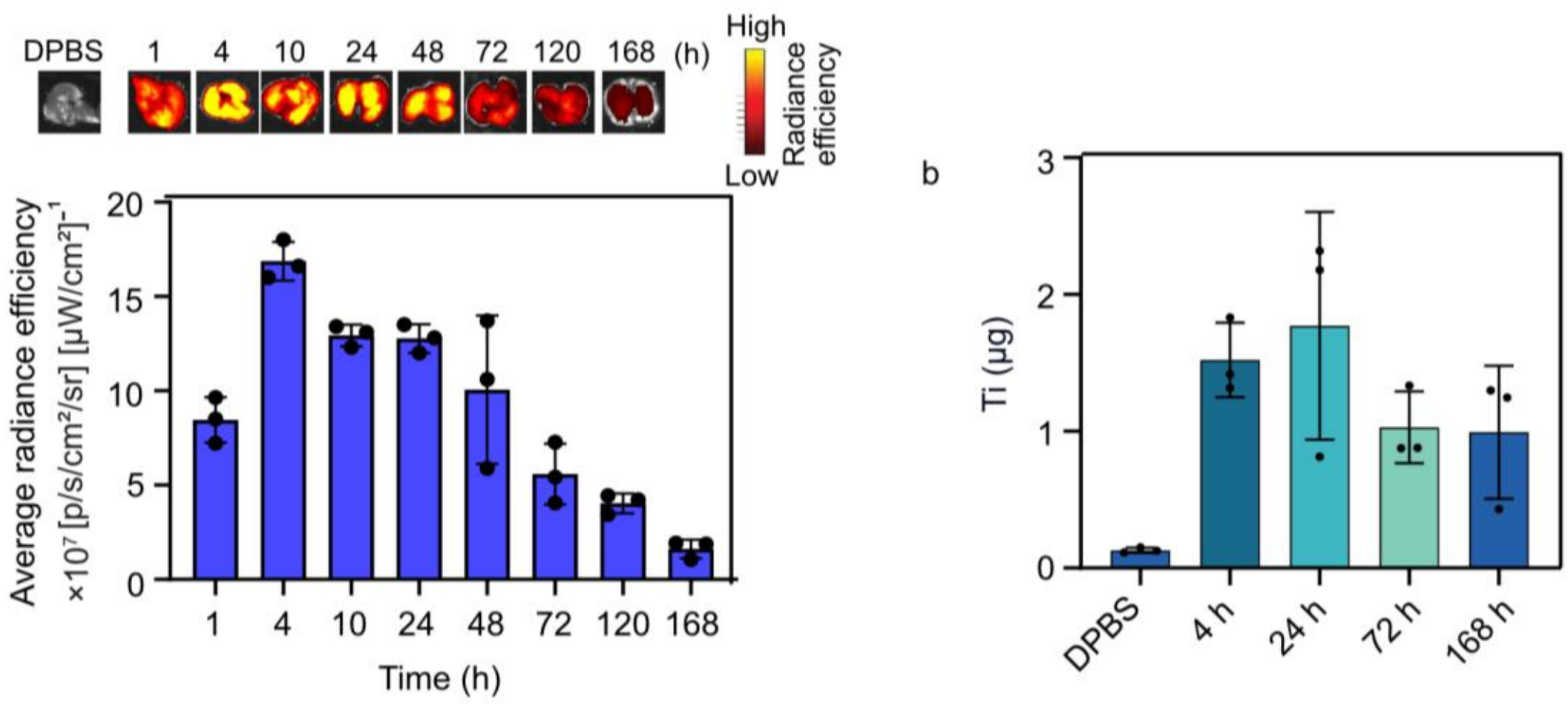

Supplementary Fig. 3 | Time-dependent lung tropism of (Ti^{IV})MPN NPs. a,b, Time-dependent lung deposition of (Ti^{IV})MPN NPs evaluated in terms of average radiance efficiency (i.e., fluorescence intensity) of Rh800 (**a**) and of the mass of Ti (**b**). Data in (**a**) were obtained using IVIS and data in (**b**) were obtained by ICP-MS. The unit of radiance efficiency is $[p/s/cm^2/sr][\mu W/cm^2]^{-1}$. $n = 3$ mice per group; the data are presented as mean ± SD. NP composition: 2 kDa PEG, BSA, TA, and Ti^{IV}; Ti^{IV}-to-TA molar ratio is 2:1.

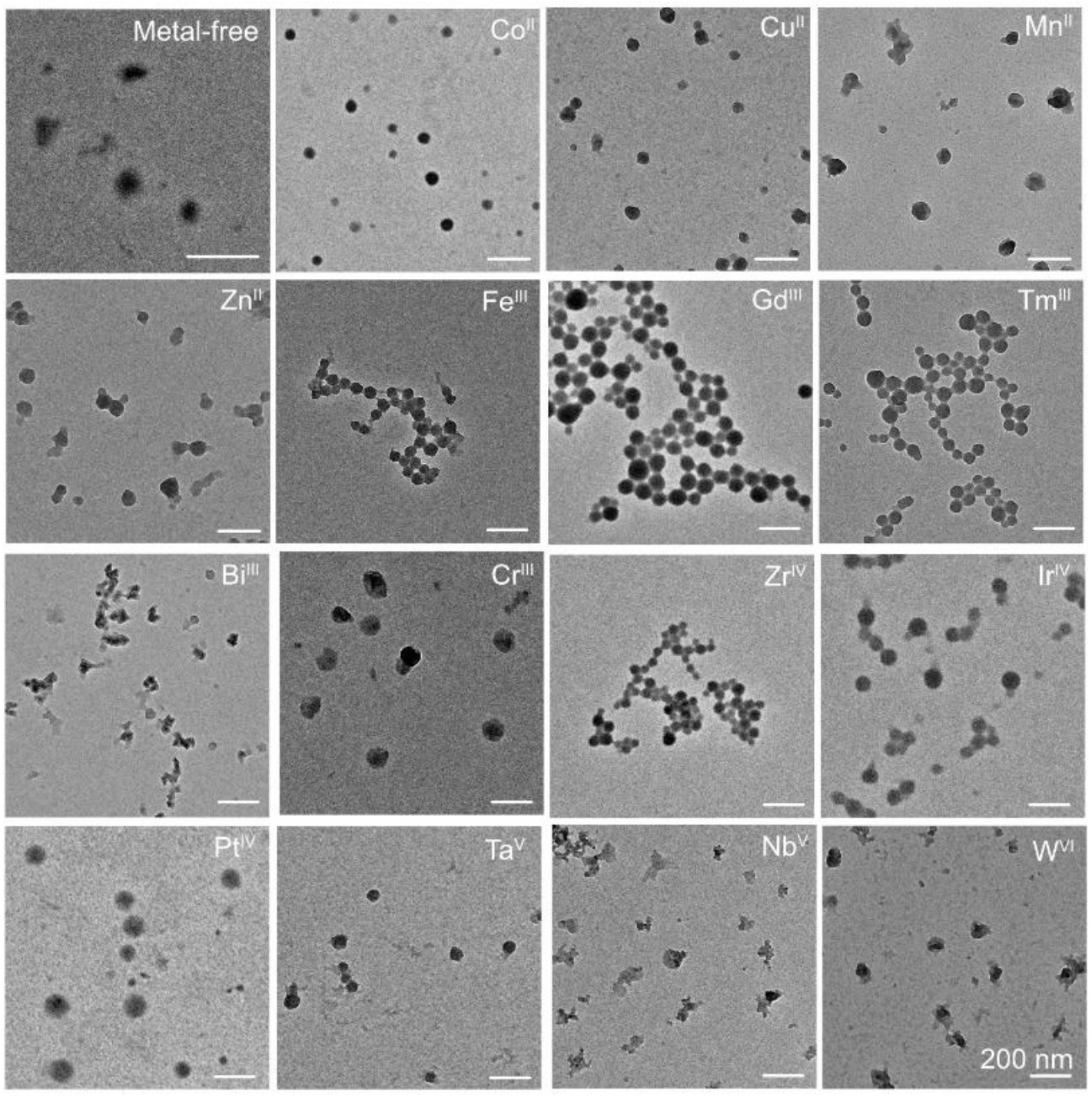

Supplementary Fig. 4 | TEM images of MPN NPs before and after the incorporation of different metal ions. The NPs are comprised of 2 kDa PEG, BSA, and TA, with or without metal ions; metal ion-to-TA molar ratio is 2:1.

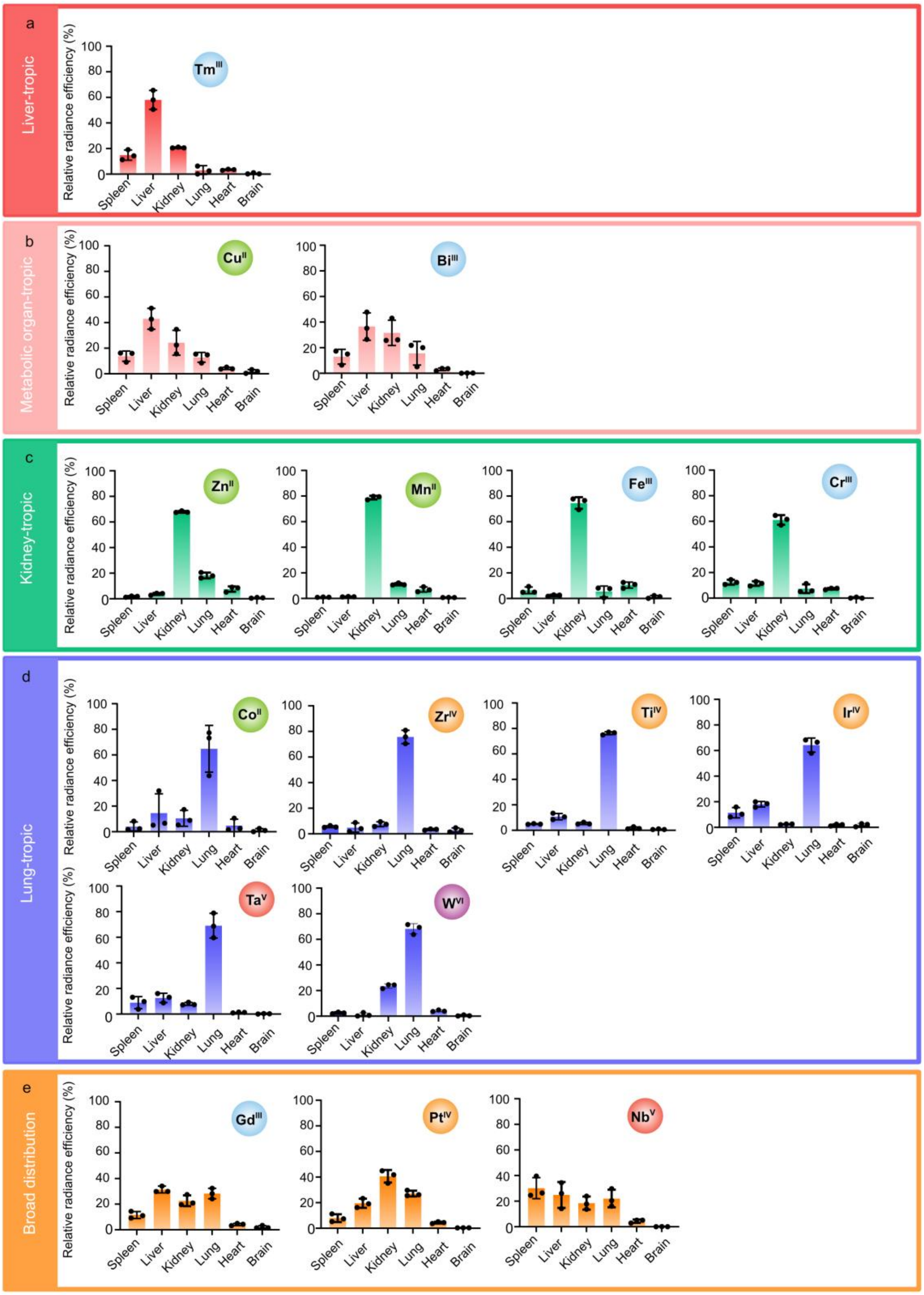

Supplementary Fig. 5 | Organ tropism enabled by MPN NPs after incorporating different metal ions. a–e, Biodistribution (by tracking Rh800 signal on IVIS) of liver-tropic (**a**), metabolic organ-tropic (**b**), kidney-tropic (**c**), lung-tropic (**d**), and broadly distributed (**e**) organ-tropic MPN NPs 6 h post-IV injection into C57BL/6J mice. $n = 3$ mice per group; and data are presented as mean ± SD. The dosage of MPN NPs was 5 mg kg^{-1}. NP composition: PEG (2 kDa), BSA, TA, and selected metal ions; metal ion-to-TA molar ratio is 2:1.

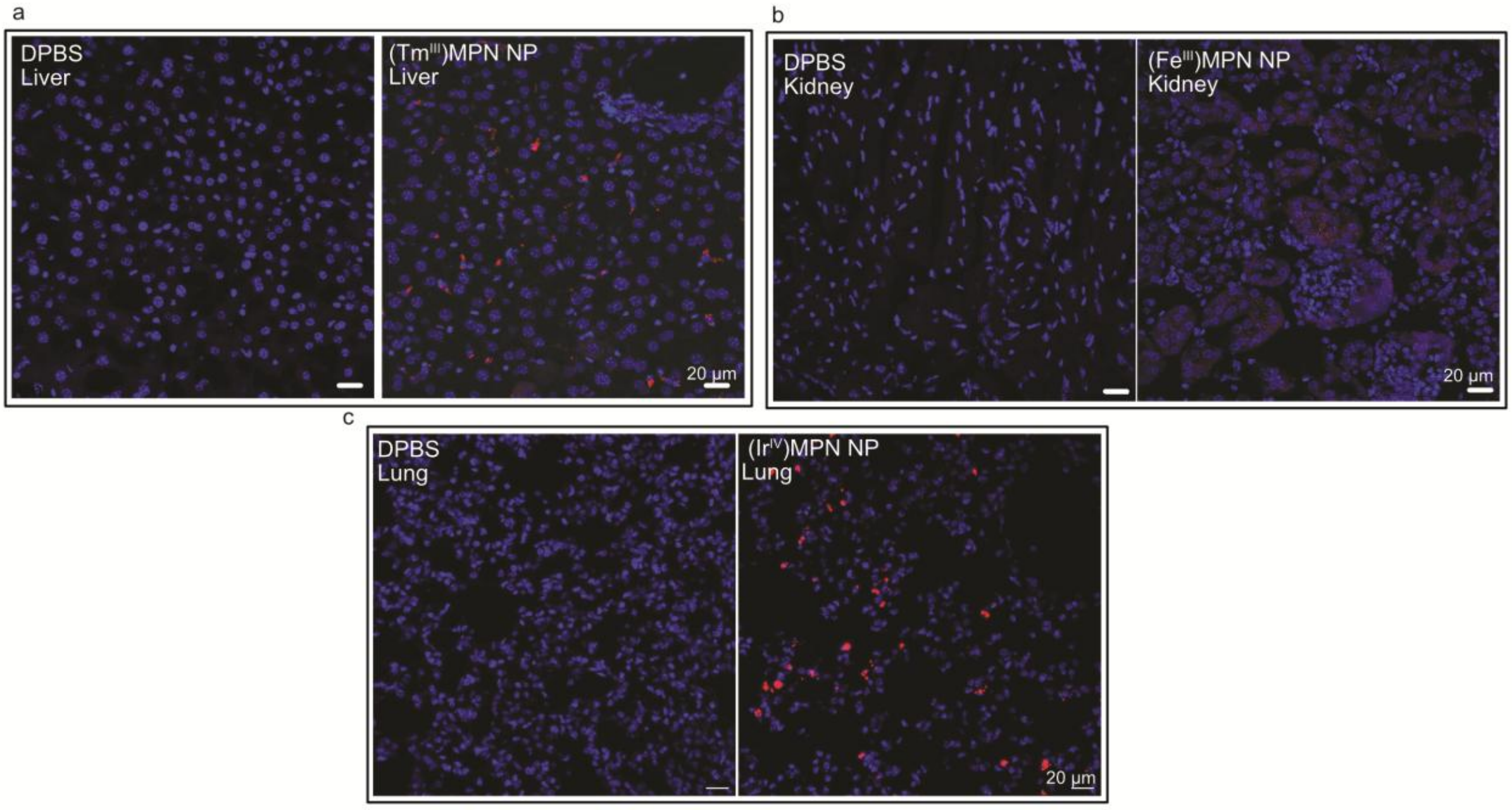

Supplementary Fig. 6 | Cellular association of MPN NPs comprising different metal ions on sectioned tissues. a–c, Representative confocal laser scanning microscopy (CLSM) images of sectioned liver (**a**), kidney (**b**), and lung (**c**) tissues 6 h post-IV injection of liver-tropic, kidney-tropic, and lung-tropic MPN NPs, respectively. The dosage of NPs was 5 mg kg^{-1}. NP composition: PEG (2 kDa), BSA, TA, and Tm^{III} (liver-tropic), Fe^{III} (kidney-tropic), or Ir^{IV} (lung-tropic); metal ion-to-TA molar ratio is 2:1. Cell nuclei were stained with DAPI (blue) and MPN NPs were labeled with Rh800 (red).

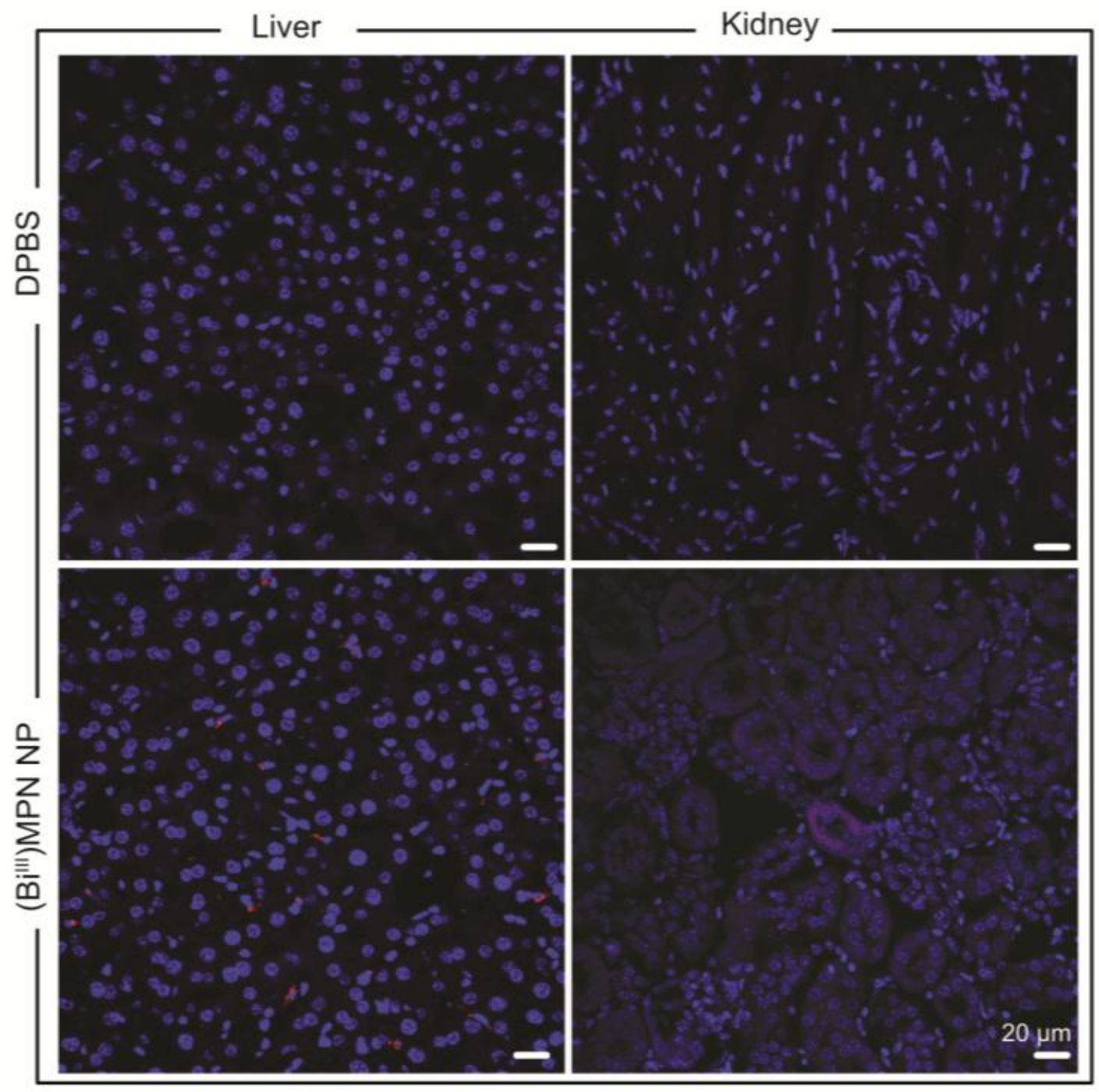

Supplementary Fig. 7 | Representative CLSM images of sectioned liver and kidney tissues 6 h post-IV injection of MPN NPs comprising metabolic organ-tropic metal ions. The NP dosage was 5 mg kg^{-1}. NP composition: PEG (2 kDa), BSA, TA, and Bi^{III}; metal ion-to-TA molar ratio is 2:1. Cell nuclei were stained with DAPI (blue) and MPN NPs were labeled with Rh800 (red).

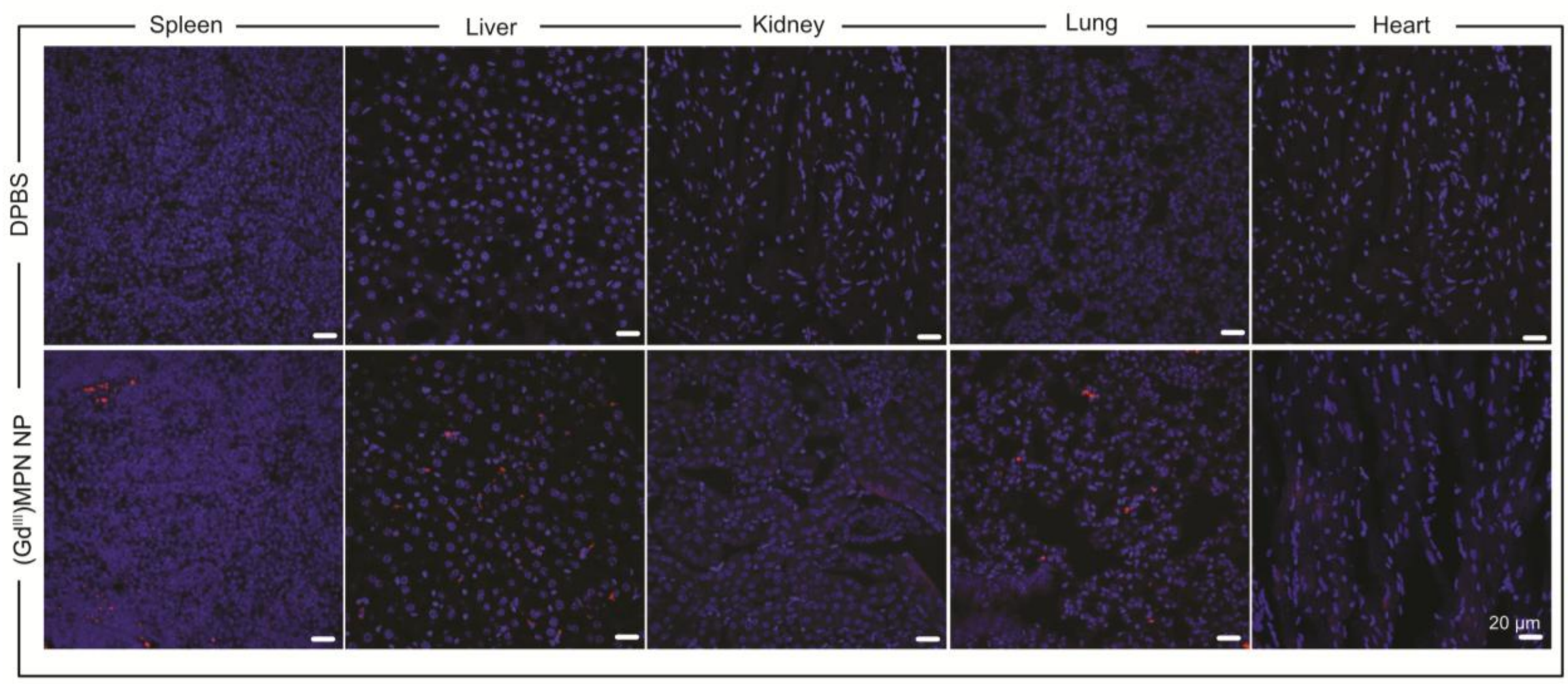

Supplementary Fig. 8 | Representative CLSM images of sectioned spleen, liver, kidney, lung, and heart tissues 6 h post-IV injection of MPN NP comprising metal ions that enable broad distribution. The NP dosage was 5 mg kg^{-1}. NP composition: PEG (2 kDa), BSA, TA, and Gd^{III}; metal ion-to-TA molar ratio is 2:1. Cell nuclei were stained with DAPI (blue) and MPN NPs were labeled with Rh800 (red).

a

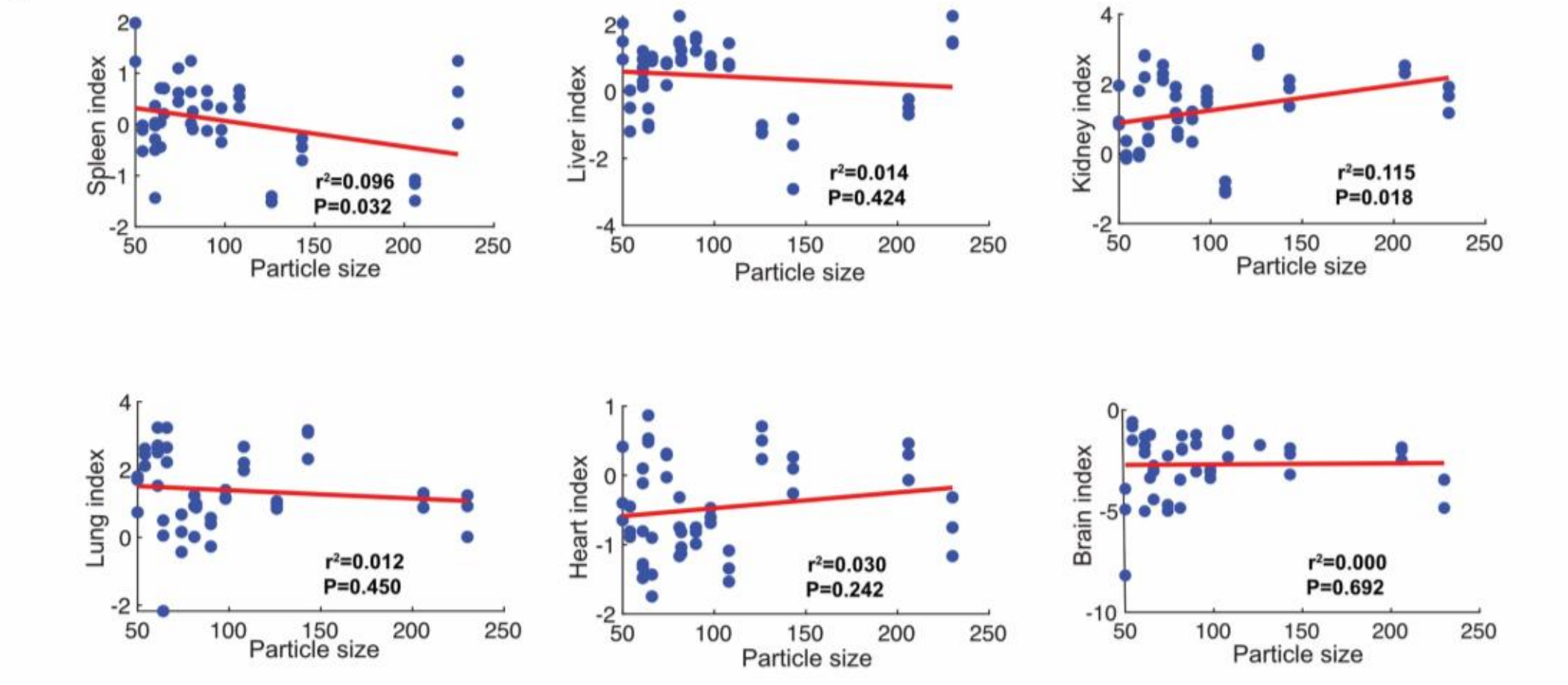

b

Organ	Spleen	Liver	Kidney	Lung	Heart	Brain
Pearson's correlation coefficient (*r*)	-0.31	-0.12	0.34	-0.11	0.17	0.02

Supplementary Fig. 9 | Linear regression analysis of the correlation between individual organ deposition and the size of MPN NPs comprising different metal ions. a, Scatter plots with the corresponding linear regression trend lines. r^2, ranging from 0 to 1, measures the proportion of the variance in the dependent variable. r^2 closer to 1 indicates a better fit of the data by the model. P evaluates the significance of individual variables within the model. A low P-value (i.e., <0.05) indicates a high likelihood of a variable influencing organ deposition. **b,** Pearson's correlation coefficients, corresponding to the correlation between particle size and the extent of deposition in harvested organs.

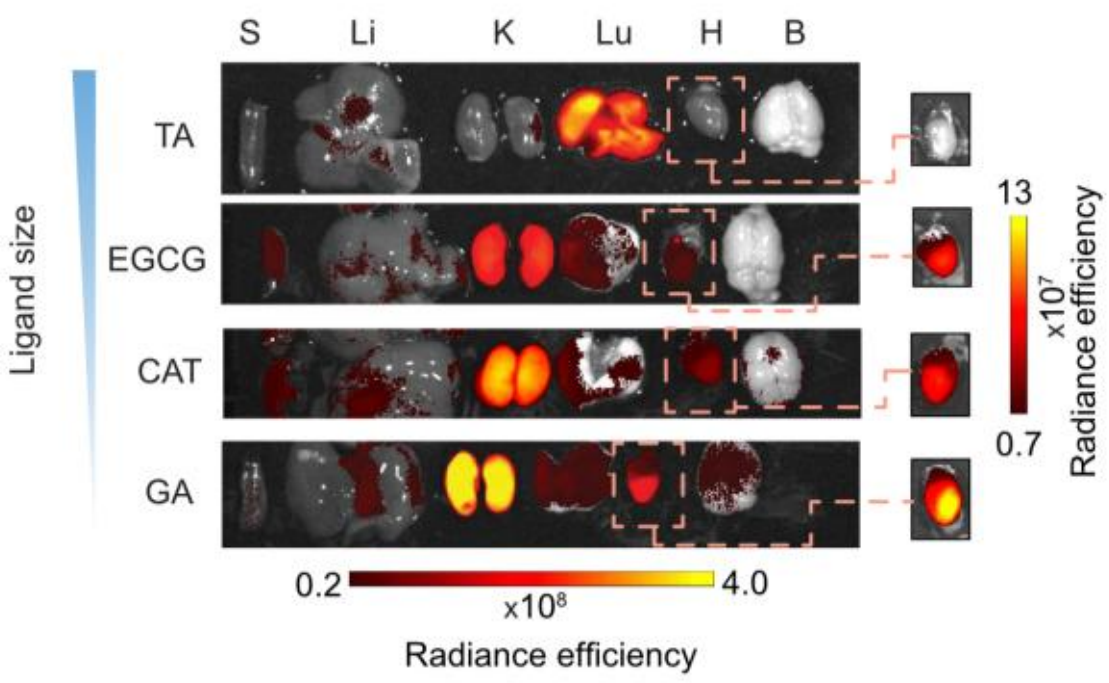

Supplementary Fig. 10 | Organ distribution of Ti^{IV}-incorporated MPN NPs comprising different phenolic ligands. Representative images were captured on IVIS 6 h post-IV injection of these NPs. The NP dosage was 5 mg kg^{-1}. IVIS images of the heart on the right at a higher magnification of fluorescence intensity were captured by only imaging the heart. This allowed for a clearer distinction of fluorescence intensity within the heart. The unit of radiance efficiency is [p/s/cm^2/sr][μW/cm^2]$^{-1}$. NP composition: 2 kDa PEG, BSA, Ti^{IV}, and phenolic ligands (TA, EGCG, CAT, or GA); Ti^{IV}-to-phenolic ligand molar ratio is 2:1.

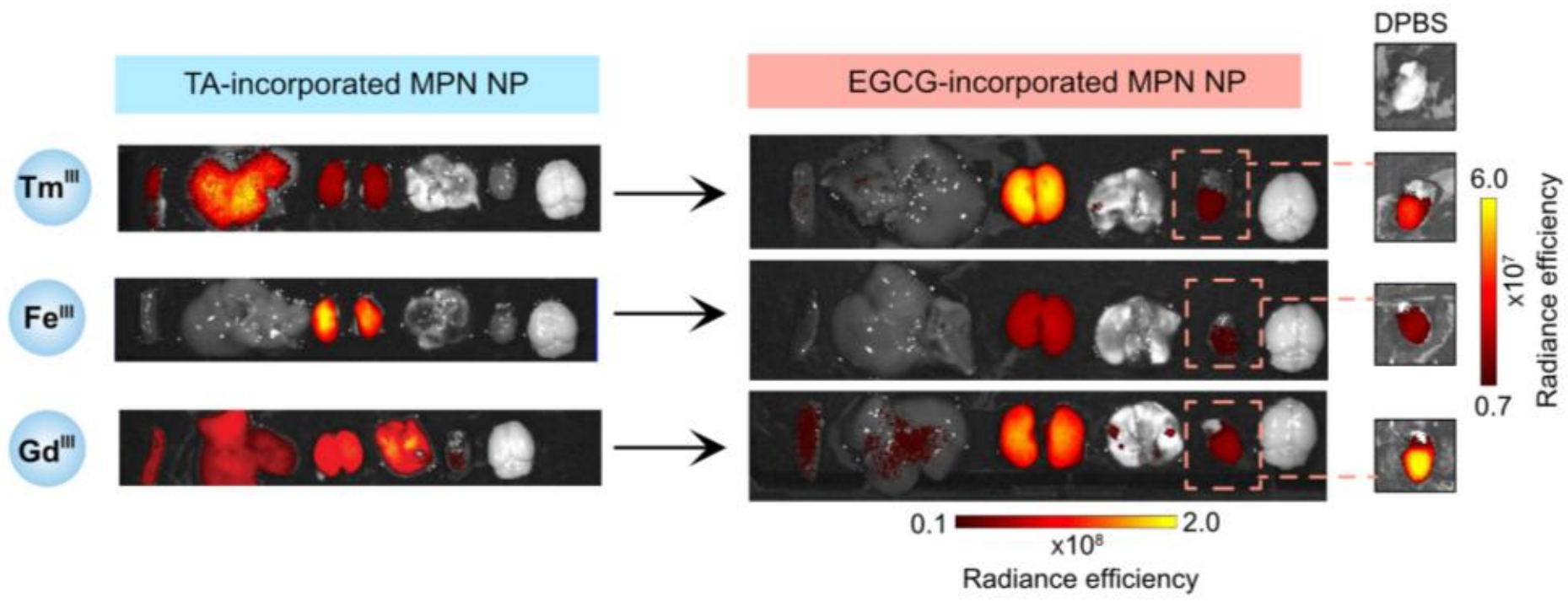

Supplementary Fig. 11 | Tuning of organ distribution tropism of MPN NP substrates comprising different metal ions after replacing TA with EGCG. TA-incorporated MPN NPs led to negligible deposition in the heart, whereas replacing TA with EGCG increased heart deposition. IVIS images of heart on the right at a higher magnification of fluorescence intensity were captured by only imaging the heart. Images were captured by IVIS 6 h post-IV injection of MPN NPs. The NP dosage was 5 mg kg^{-1}. The unit of radiance efficiency is [p/s/cm^2/sr][μW/cm^2]$^{-1}$. NP composition: PEG (2 kDa), BSA, TA or EGCG, and metal ions (Tm^{III}, Fe^{III}, or Gd^{III}); metal ion-to-phenolic ligand molar ratio is 2:1.

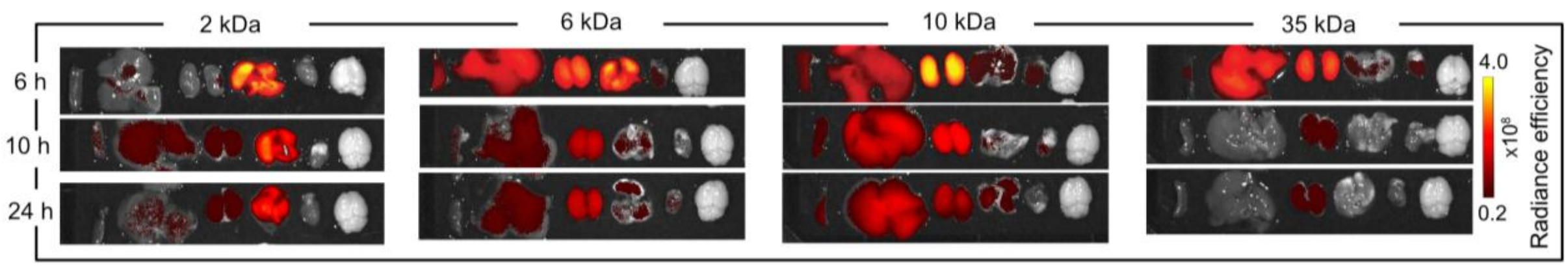

Supplementary Fig. 12 | Representative IVIS images of time-dependent biodistribution of MPN NPs comprising PEG of different M_w. The NP dosage was 5 mg kg^{-1}. NP composition: PEG (2, 6, 10, or 35 kDa), BSA, TA, and Ti^{IV}; Ti^{IV}-to-TA molar ratio is 2:1.

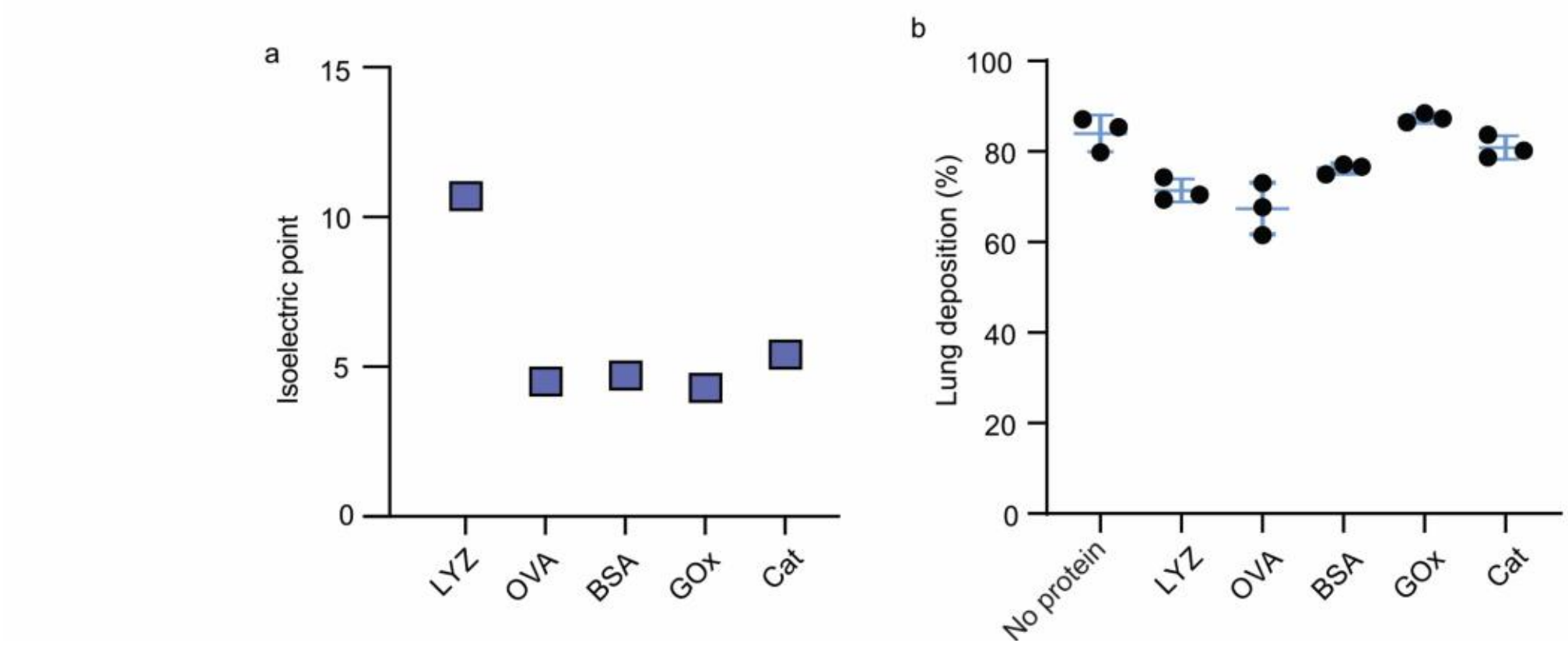

Supplementary Fig. 13 | Lung deposition of MPN NPs comprised of different proteins 6 h after IV administration into C57BL/6J mice. (**a**) Isoelectric point of encapsulated proteins. LYZ, lysozyme; OVA, ovalbumin; BSA, bovine serum albumin; GOx, glucose oxidase; Cat, catalase. (**b**) Quantitative results obtained on IVIS and Rh800 signal were tracked. $n = 3$ mice per group; data are presented as mean ± SD. NP composition: 2 kDa PEG, selected protein, TA, and Ti^{IV}; Ti^{IV}-to-TA molar ratio is 2:1.

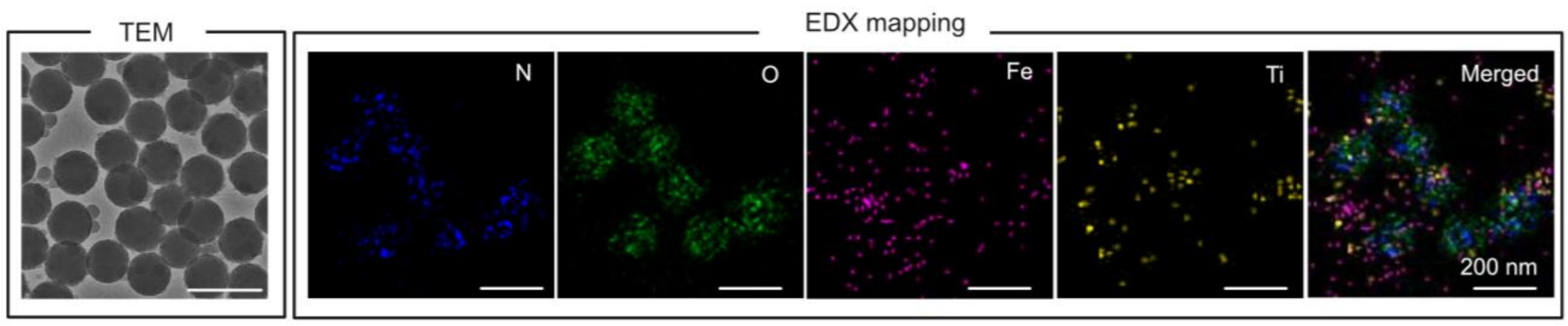

Supplementary Fig. 14 | Characterization of a lung-tropic MPN NP substrate (containing Ti^{IV}) with a kidney-tropic coating (containing Fe^{III}). Representative TEM image and EDX mapping of MPN NP@tMPN with a Ti^{IV}-dominant inner core and an Fe^{III}-dominant outer shell.

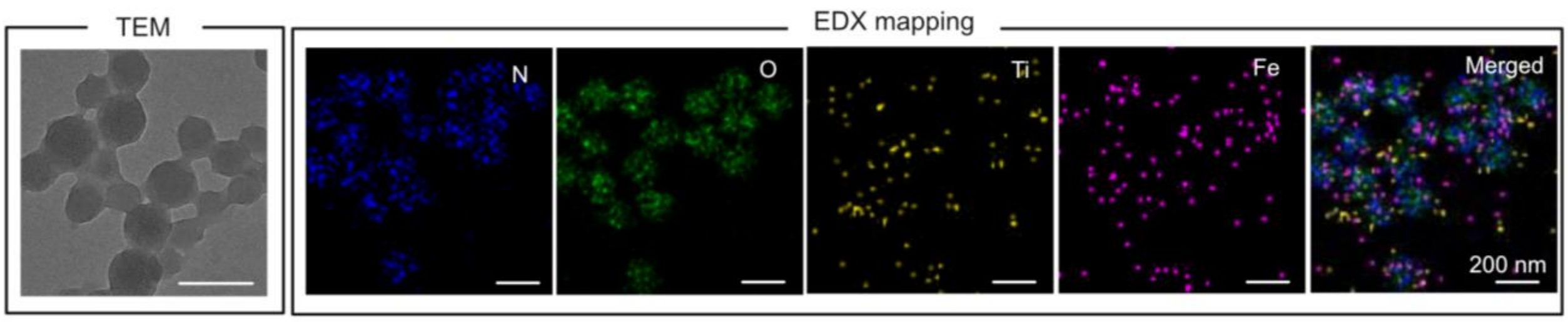

Supplementary Fig. 15 | Characterization of a kidney-tropic MPN NP substrate (containing Fe^{III}) with a lung-tropic coating (containing Ti^{IV}). Representative TEM image and EDX mapping of MPN NP@tMPN with an Fe^{III}-dominant inner core and a Ti^{IV}-dominant outer shell.

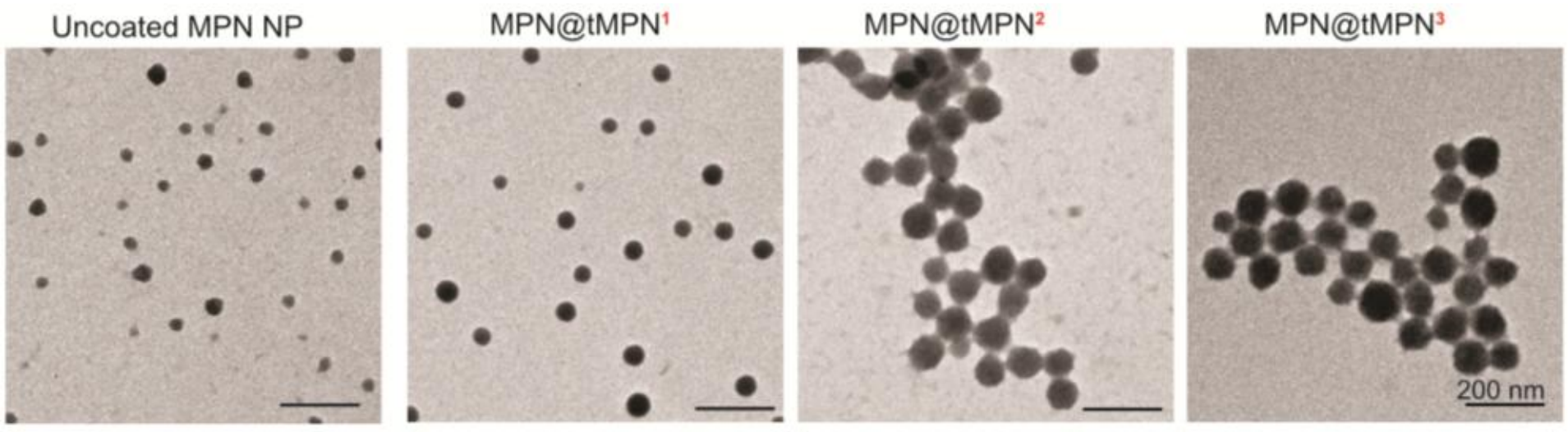

Supplementary Fig. 16 | TEM images of MPN NPs with different shell thicknesses prepared via repeated coating. The NP core and shells are comprised of PEG (2 kDa), BSA, TA, and Fe^{III}.

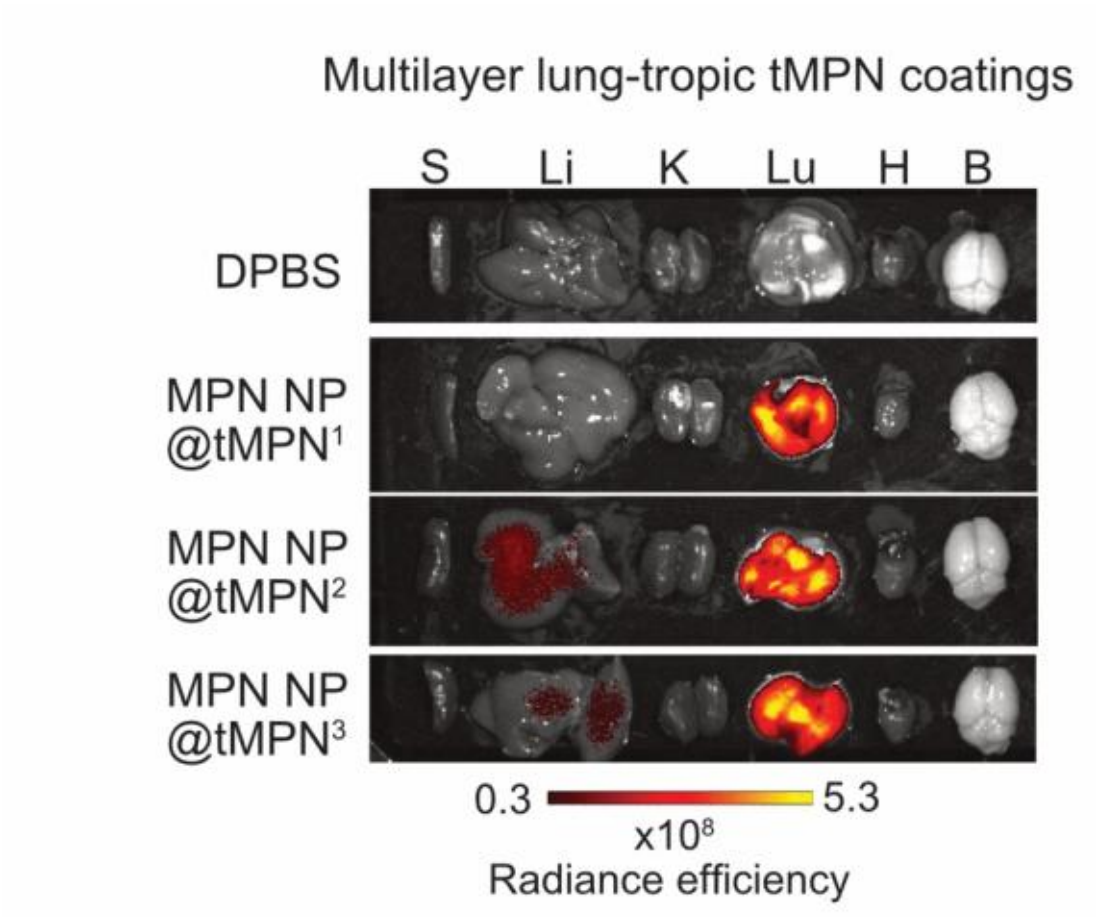

Supplementary Fig. 17 | Biodistribution of a lung-tropic MPN NP substrate (containing Ti^{IV}) with different layers of a lung-tropic coating (containing Ti^{IV}). The unit of radiance efficiency in all IVIS images is $[p/s/cm^2/sr][\mu W/cm^2]^{-1}$.

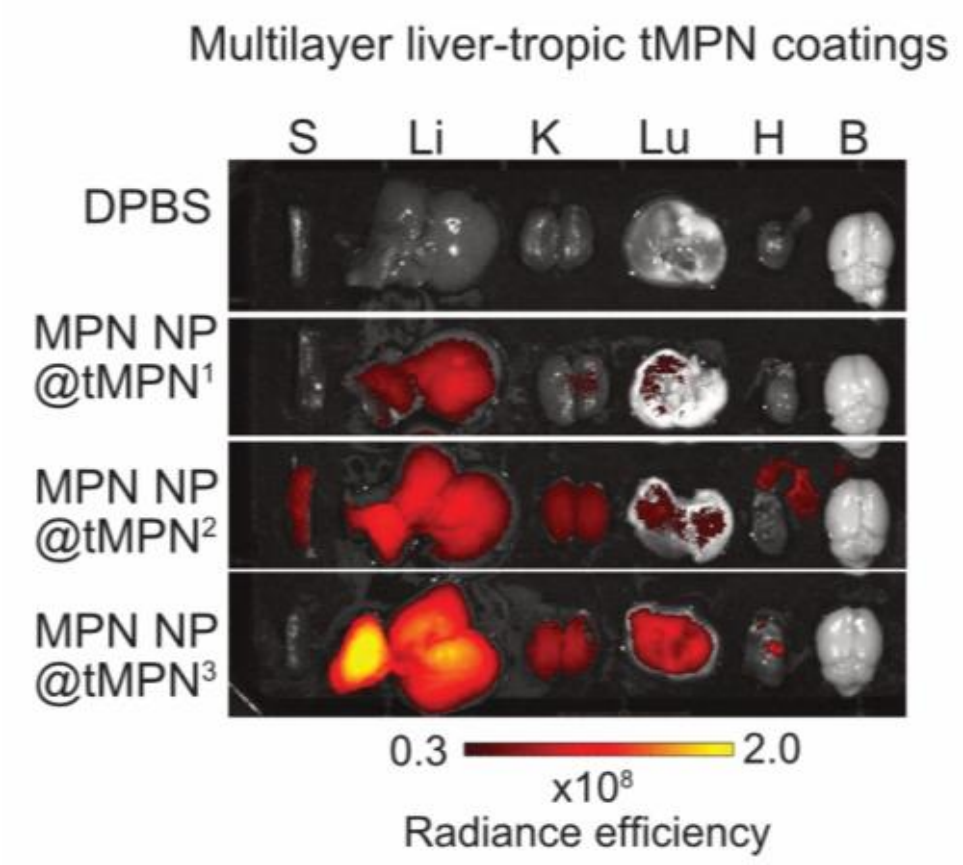

Supplementary Fig. 18 | Biodistribution of a liver-tropic MPN NP substrate (containing Tm^{III}) with different layers of a liver-tropic coating (containing Tm^{III}). The unit of radiance efficiency in all IVIS images is $[p/s/cm^2/sr][\mu W/cm^2]^{-1}$.

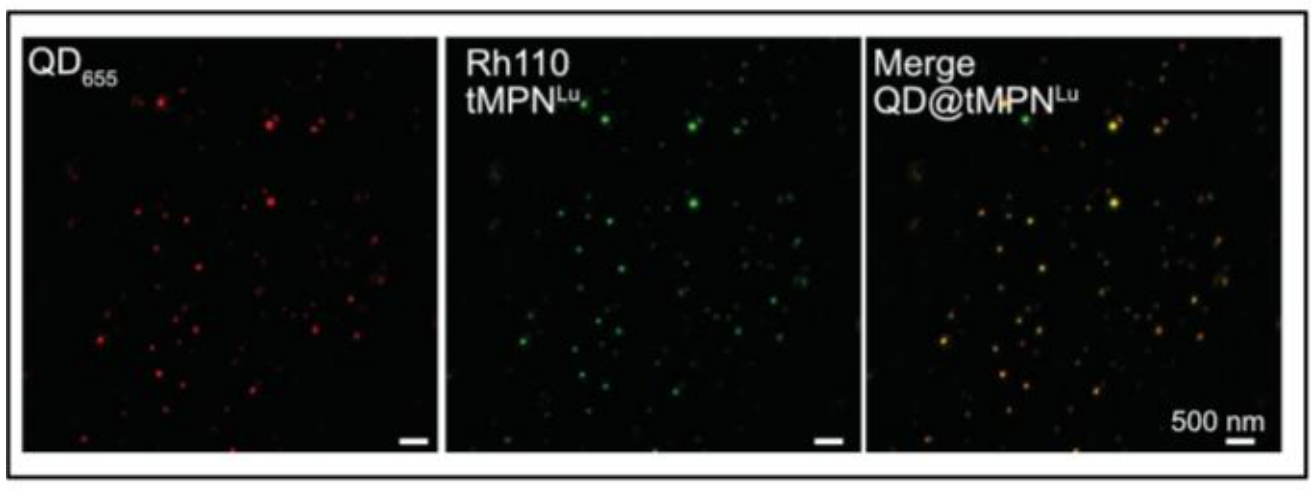

Supplementary Fig. 19 | Characterization of QD@tMPN^{Lu}. Representative lattice-SIM images showing colocalization of QD655 and Rh110 (from tMPN) after assembly. Colocalization of the two fluorophores indicates the formation of QD@tMPN^{Lu}. The composition of the tMPN^{Lu} coating was 2 kDa PEG, BSA, TA, and Co^{II}.

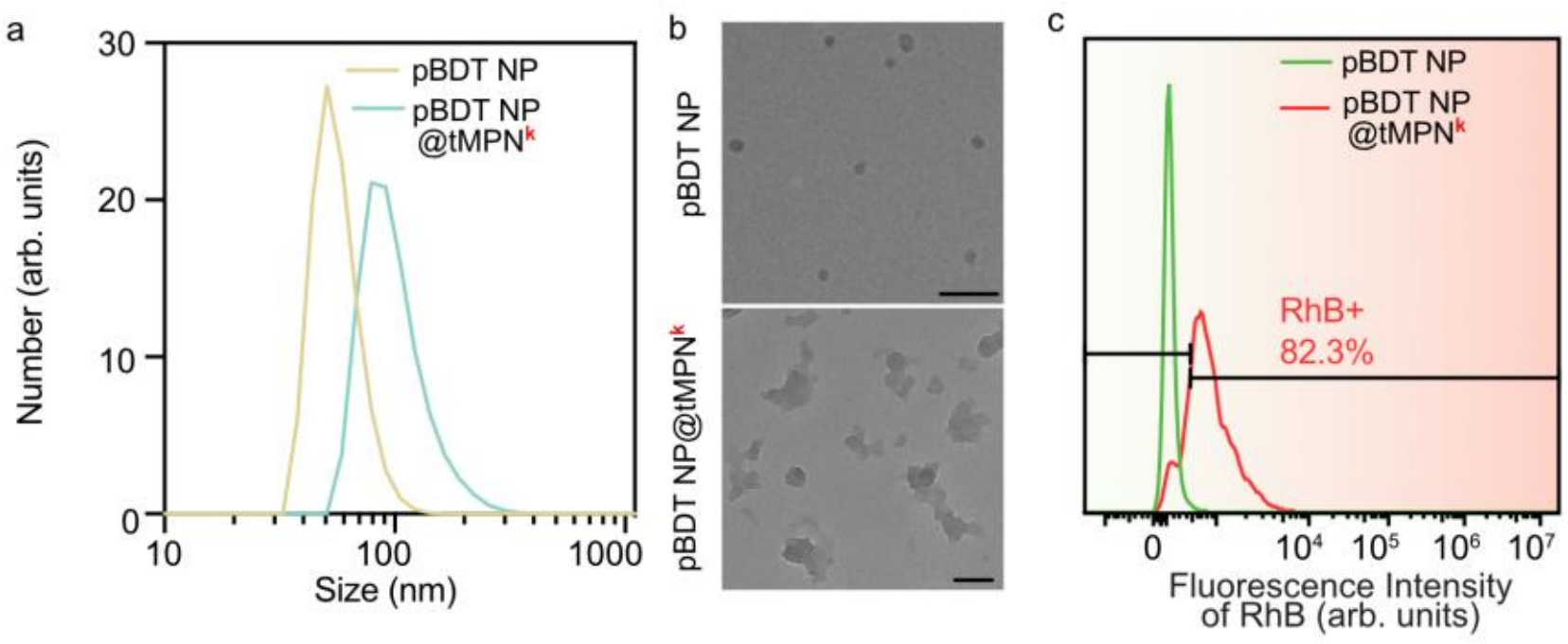

Supplementary Fig. 20 | Characterization of pBDT@tMPN^{k}. a,b, Size (diameter) distribution (**a**) and TEM images (**b**) of pBDT NPs and pBDT NP@tMPN^{K}. Scale bars in TEM images are 200 nm. **c,** Coating efficiency of tMPN^{K} on pBDT NPs, as evaluated from fluorescence intensity measurements on a CytoFLEX nano flow cytometer. Rhodamine B (RhB) was selected as the fluorescent dye to probe tMPN^{K}. The composition of the tMPN^{K} coating was 2 kDa PEG, BSA, TA, and Cr^{III}.

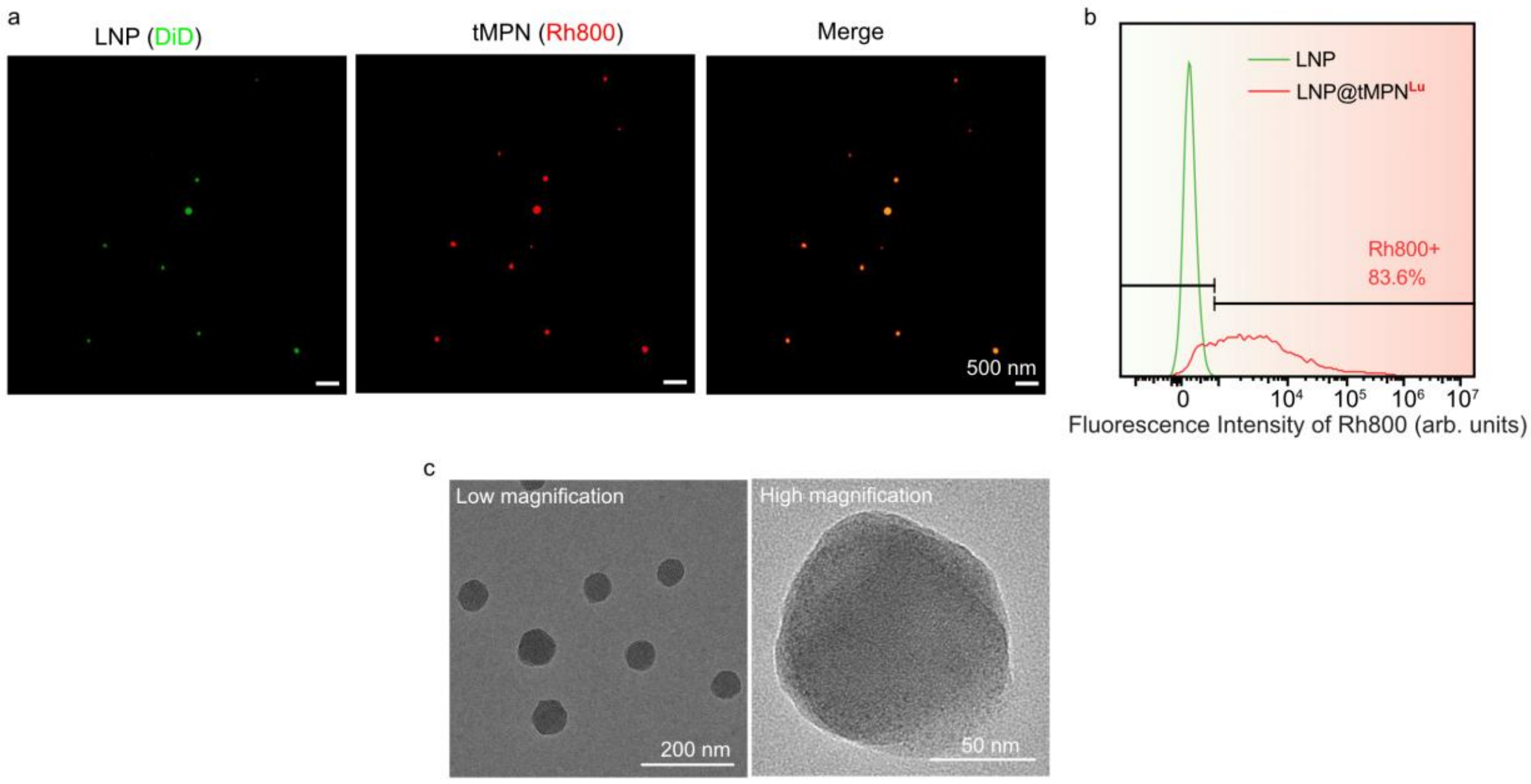

Supplementary Fig. 21 | Characterization of LNP@tMPN^{Lu}. a, Representative lattice-SIM images showing the colocalization of Rh800 and 1,1′-dioctadecyl-3,3,3′,3′-tetramethylindodicarbocyanine (DiD) signals. The colocalization of the two fluorophores indicates the formation of tMPN^{Lu} on LNPs. **b,** Coating efficiency of tMPN^{Lu} on LNPs, as evaluated from fluorescence intensity measurements on a CytoFLEX nano flow cytometer. Rh800 was used as the fluorescent dye to probe tMPN^{Lu}. **c,** TEM images of LNP@tMPN^{Lu} at low (scale bar 200 nm) and high (scale bar 50 nm) magnification. The composition of the tMPN^{Lu} coating was 2 kDa PEG, BSA, TA, and Co^{II}.

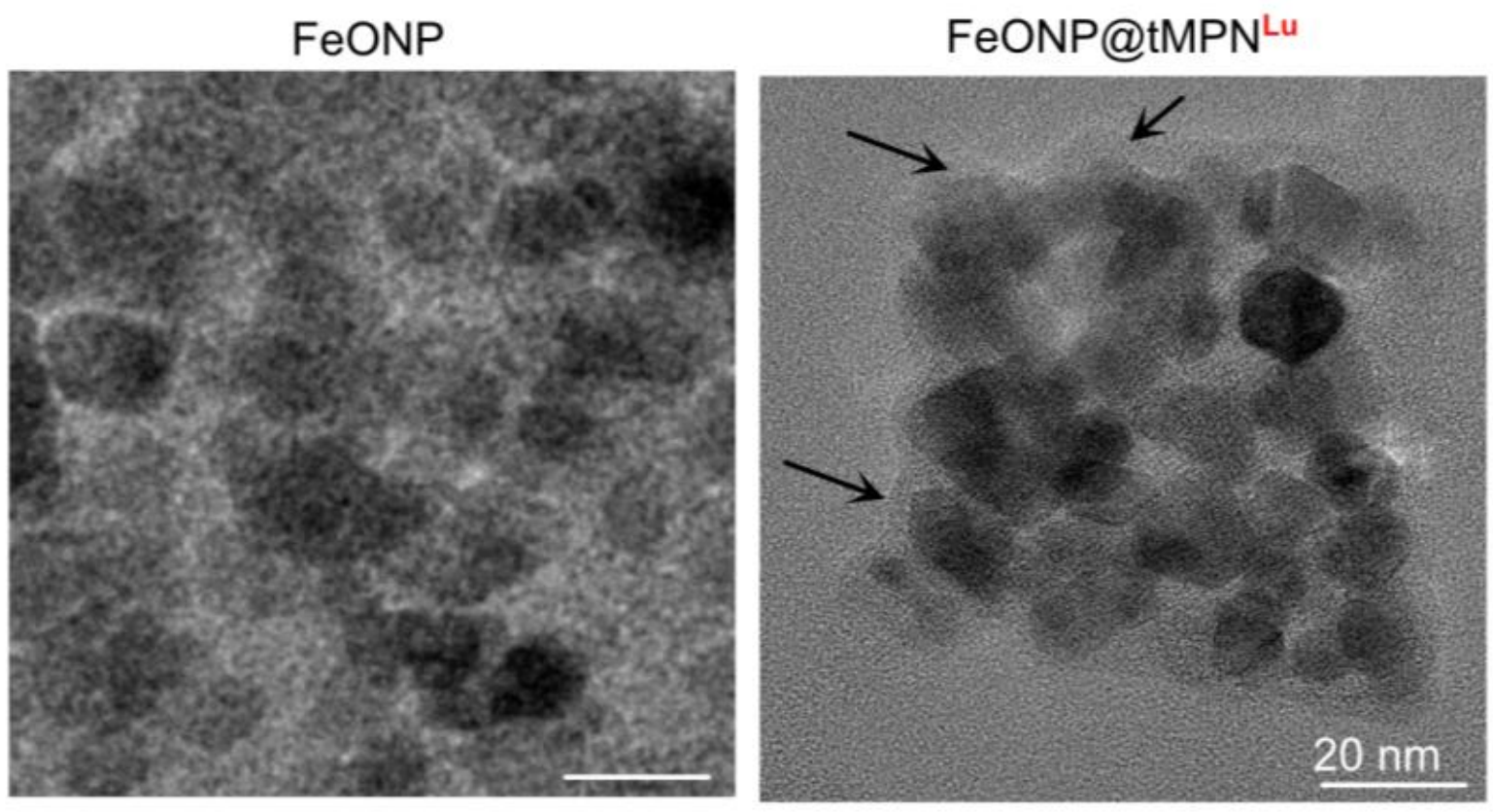

Supplementary Fig. 22 | Characterization of FeONP@tMPN^{Lu}. Representative TEM images of FeONPs and FeONP@tMPN^{Lu}. The composition of the tMPN^{Lu} coating was 2 kDa PEG, BSA, TA, and Ti^{IV}. The black arrows indicate representative surface changes upon tMPN^{Lu} coating formation.

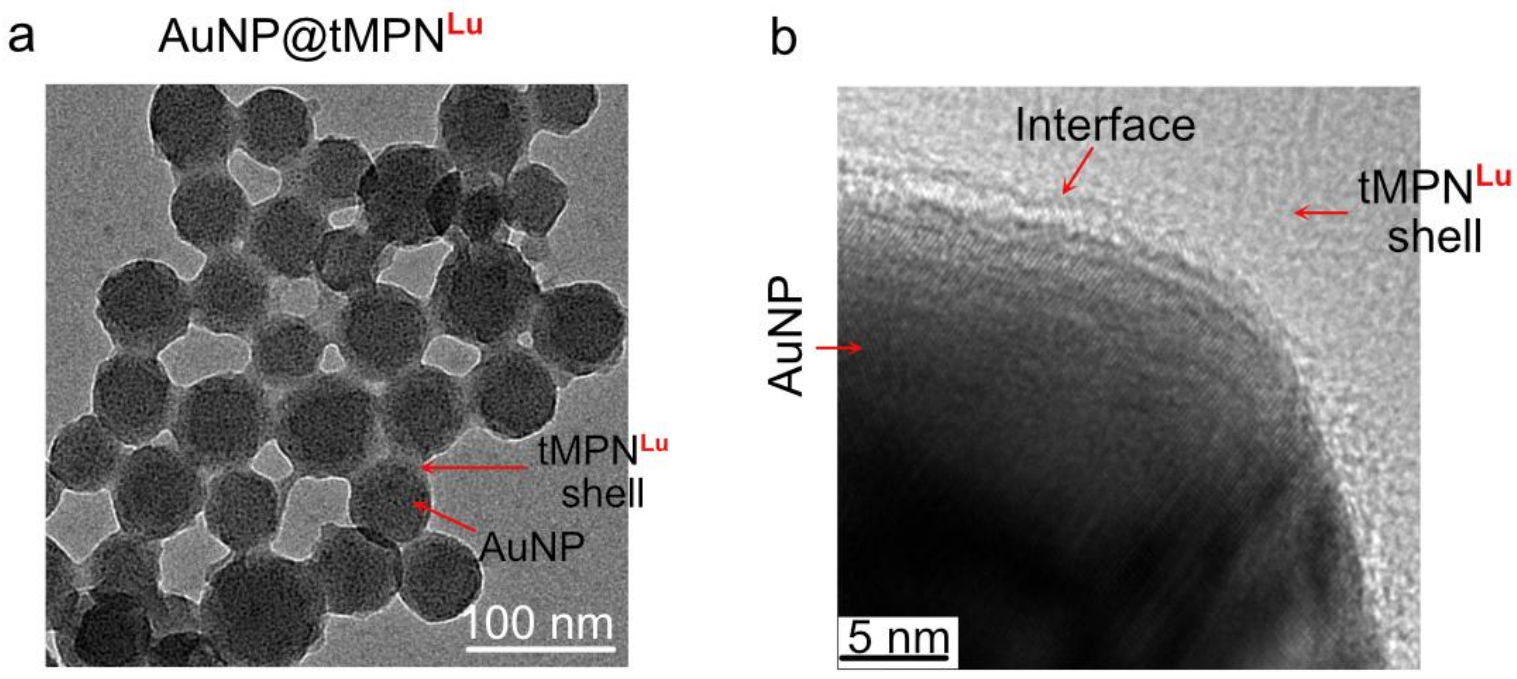

Supplementary Fig. 23 | Characterization of AuNP@tMPN^{Lu}. a,b, Representative TEM images of AuNP@tMPN^{Lu} at low (**a**) and high (**b**) magnification. The composition of the tMPN^{Lu} coating was 2 kDa PEG, BSA, TA, and Ti^{IV}.

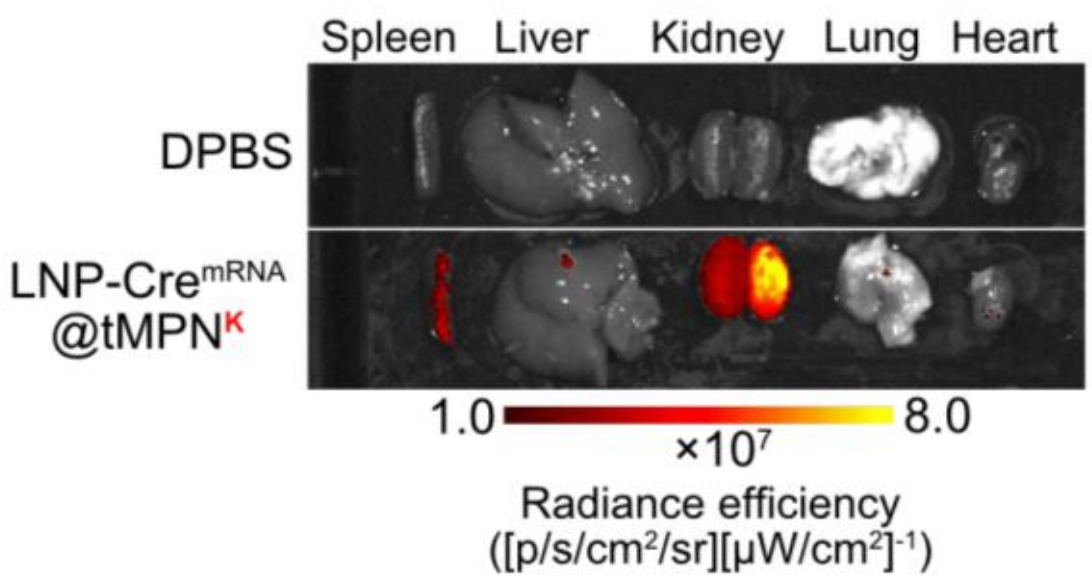

Supplementary Fig. 24 | Representative IVIS images showing organ tropism redirection of LNPs. The red fluorescence is activated tdTom, indicating successful Cre^{mRNA} transfection. Images were captured 3 days post-IV administration of Ai14 mice with mRNA at a dosage of 5 µg per mouse. The composition of the tMPN^{K} coating was 2 kDa PEG, BSA, TA, and Cr^{III}.

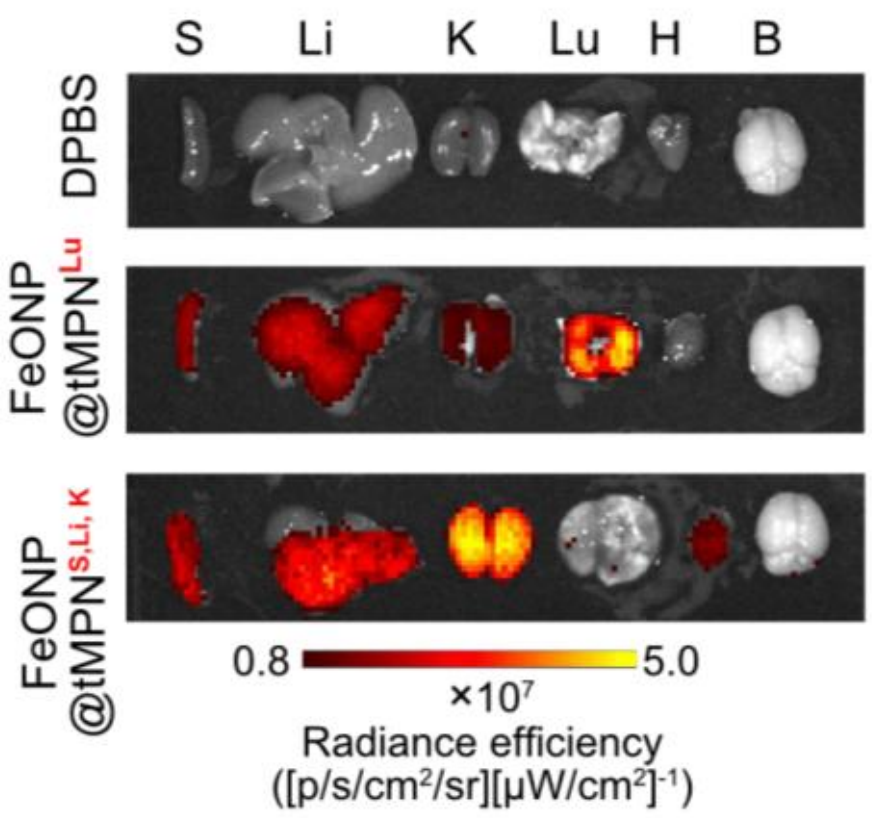

Supplementary Fig. 25 | Representative IVIS images showing organ tropism redirection of FeONPs. The red fluorescence represents Rh800 incorporated into tMPNs. Images were captured 6 h post-IV administration in C57BL/6J mice. The composition of the tMPN^{Lu} coating was 2 kDa PEG, BSA, TA, and Ti^{IV}. The composition of the tMPN^{S,Li,K} coating was 20 kDa PEG, BSA, TA, and Ti^{IV}.

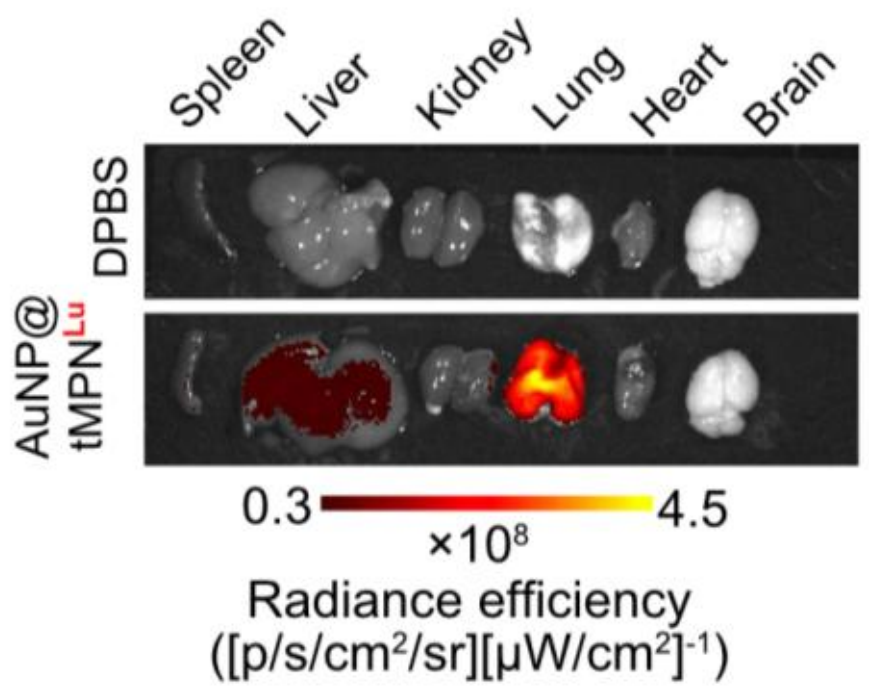

Supplementary Fig. 26 | Representative IVIS images showing organ tropism redirection of AuNPs (~50 nm). The red fluorescence represents Rh800 incorporated into tMPNs. Images were captured 6 h post-IV administration in C57BL/6J mice. The composition of the $tMPN^{Lu}$ coating was 2 kDa PEG, BSA, TA, and Ti^{IV}.

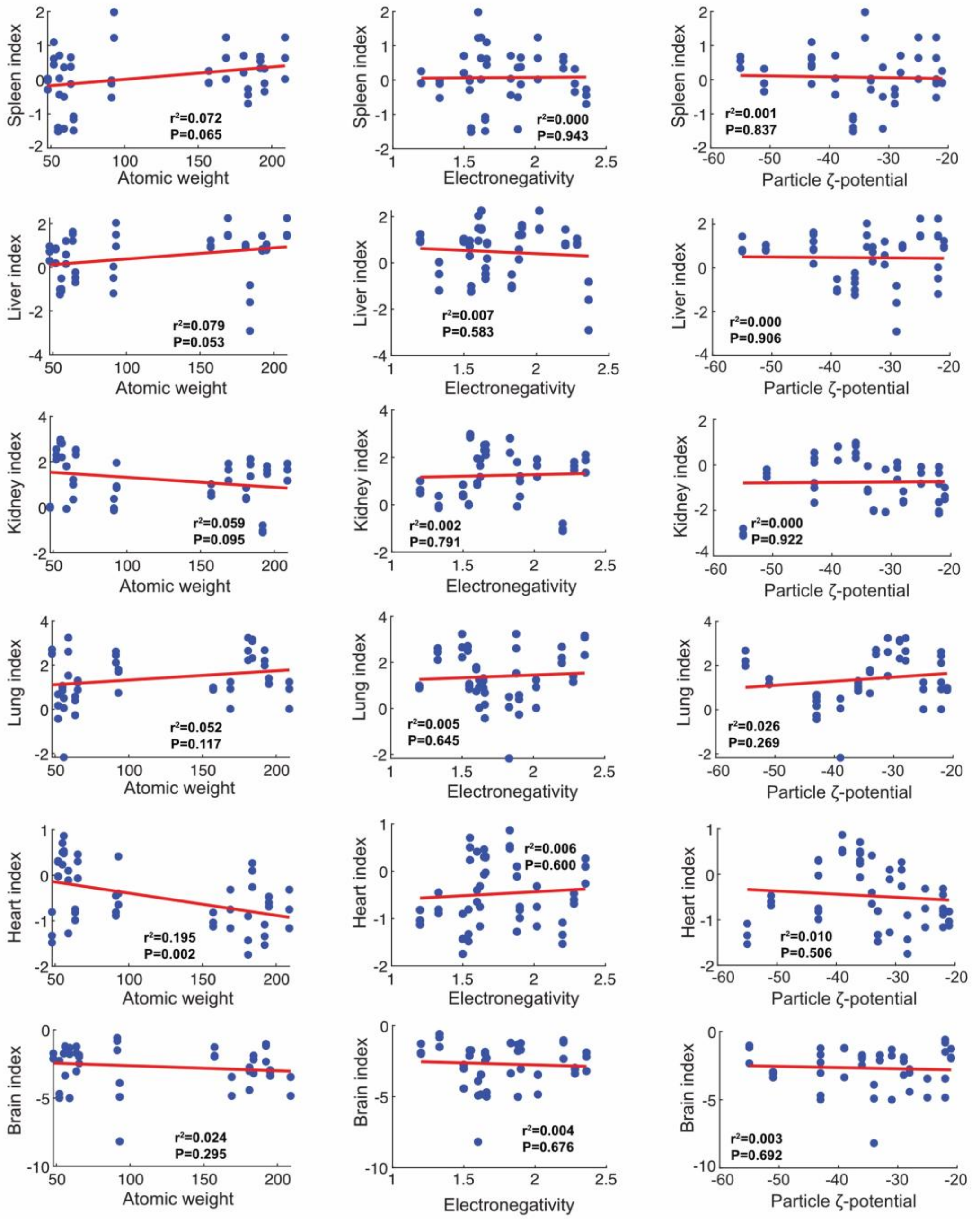

Supplementary Fig. 27 | Linear regression analysis of the correlation between individual NP organ deposition and metal atomic weight, electronegativity, and particle ζ-potential. r^2, ranging from 0 to 1, measures the proportion of the variance in the dependent variable. r^2 closer to 1 indicates a better fit of the data by the model. *P* evaluates the significance of individual variables within the model. A low *P*-value (i.e., <0.05) indicates a high likelihood of a variable influencing organ deposition.

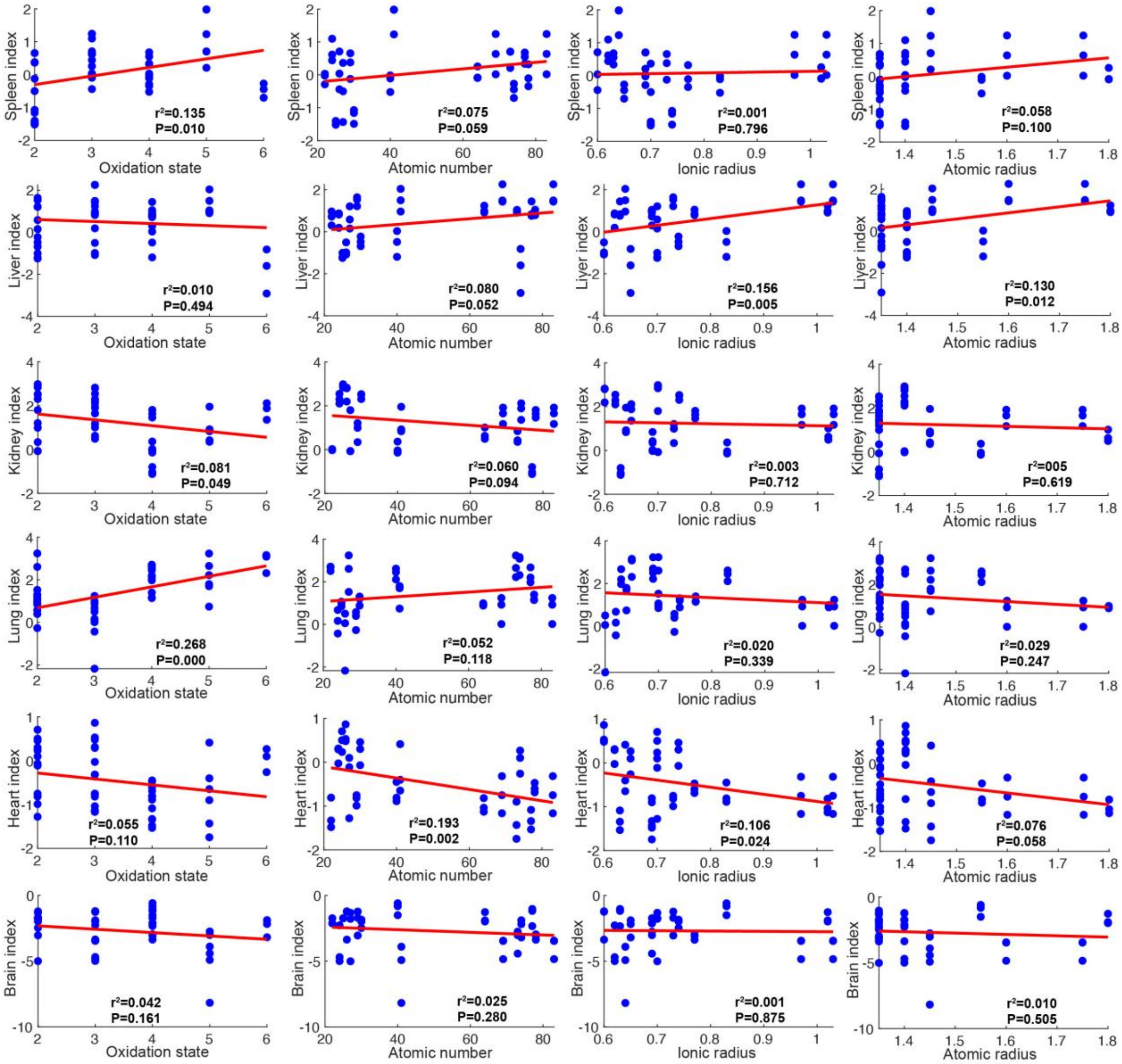

Supplementary Fig. 28 | Linear regression analysis of the correlation between individual NP organ deposition and oxidation state of metal, metal atomic number, and ionic and atomic radii of metal ions. r^2, ranging from 0 to 1, measures the proportion of the variance in the dependent variable. r^2 closer to 1 indicates a better fit of the data by the model. *P* evaluates the significance of individual variables within the model. A low *P*-value (i.e., <0.05) indicates a high likelihood of a variable influencing organ deposition.

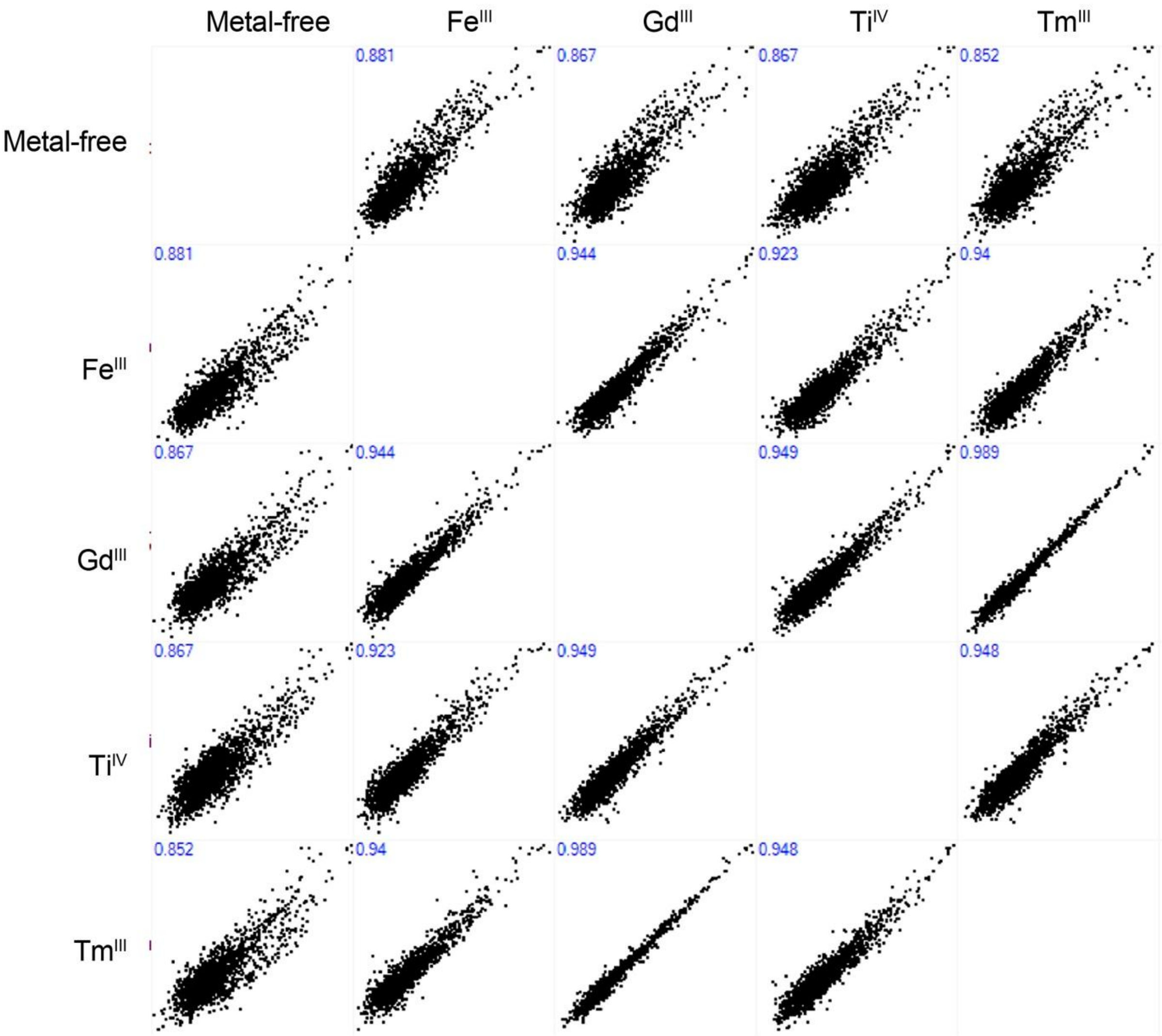

Supplementary Fig. 29 | Similarity analysis of protein corona profiles for selected organ-tropic MPN NP formulations. Correlation plots of identified plasma proteins from five types of MPN NPs with distinct organ tropisms with the Pearson correlation coefficient values.

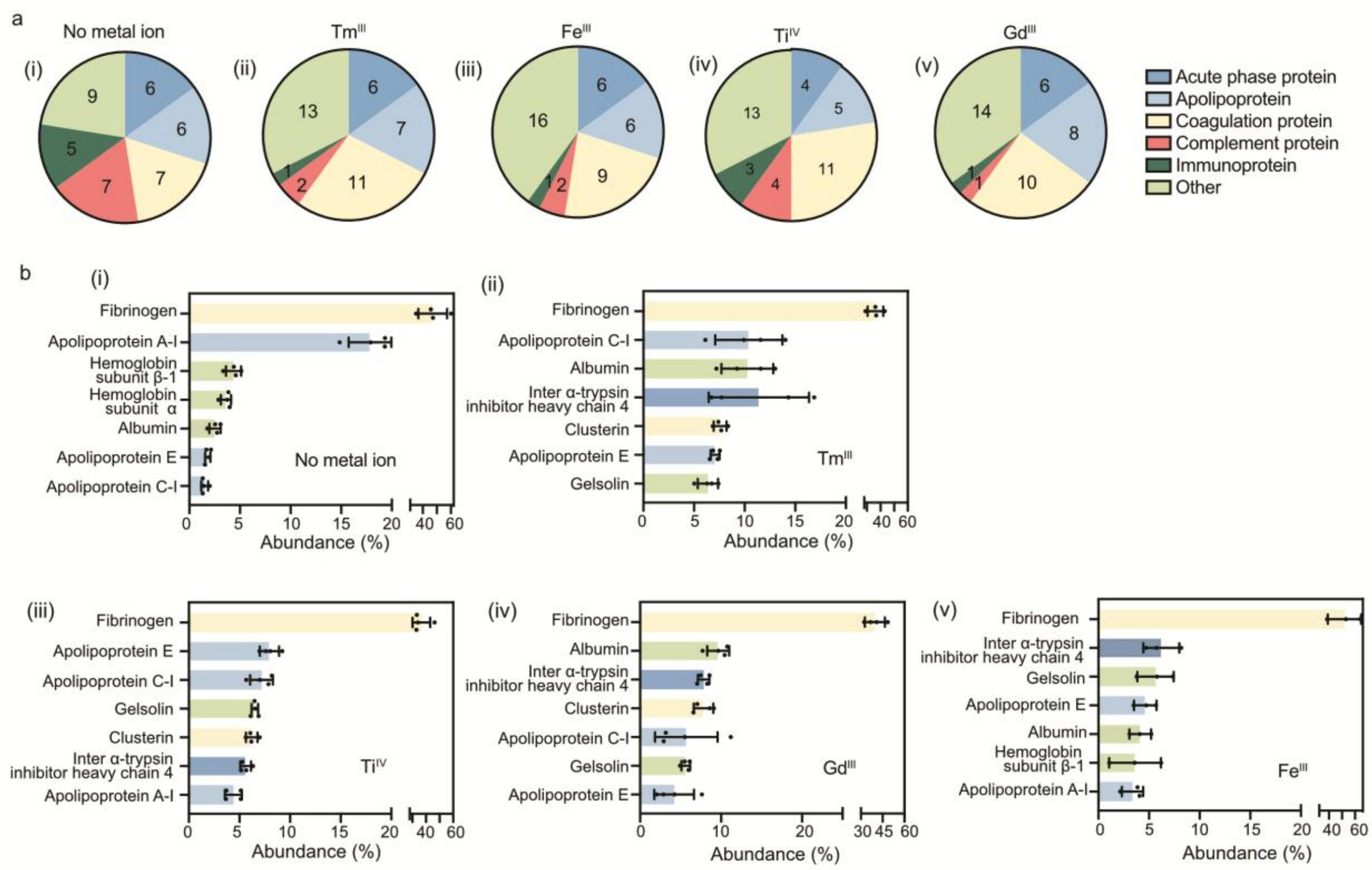

Supplementary Fig. 30 | Effect of metal ions on plasma proteins adsorbed onto the surface of MPN NPs. a, Average abundance of proteins with distinct biological functions in the protein coronas of NP formulations. Analysis was based on the top 40 most abundant plasma proteins identified in each selected MPN NPs. **b,** Top seven most abundant plasma proteins that bind to different MPN NPs prepared using different metal ions (No metal ion (i), Tm^{III} (ii), Ti^{IV} (iii), Gd^{III} (iv), Fe^{III} (v)). Note, proteins sourced from the complement protein and immunoprotein categories are absent from the top seven proteins. As alpha, beta, and gamma chains of fibrinogen constitute a substantial proportion of the proteins across all selected MPN NPs, they are grouped and analyzed collectively as "fibrinogen". NP composition: PEG (2 kDa), BSA, TA, and different metal ions. For the Fe^{III}-based NP group: n = 3 independent replicates of a single NP formulation, each incubated with aliquots from the same pooled mouse plasma sample. For the remaining groups: n = 4 independent replicates of a single NP formulation, each incubated with aliquots from the same pooled mouse plasma sample.

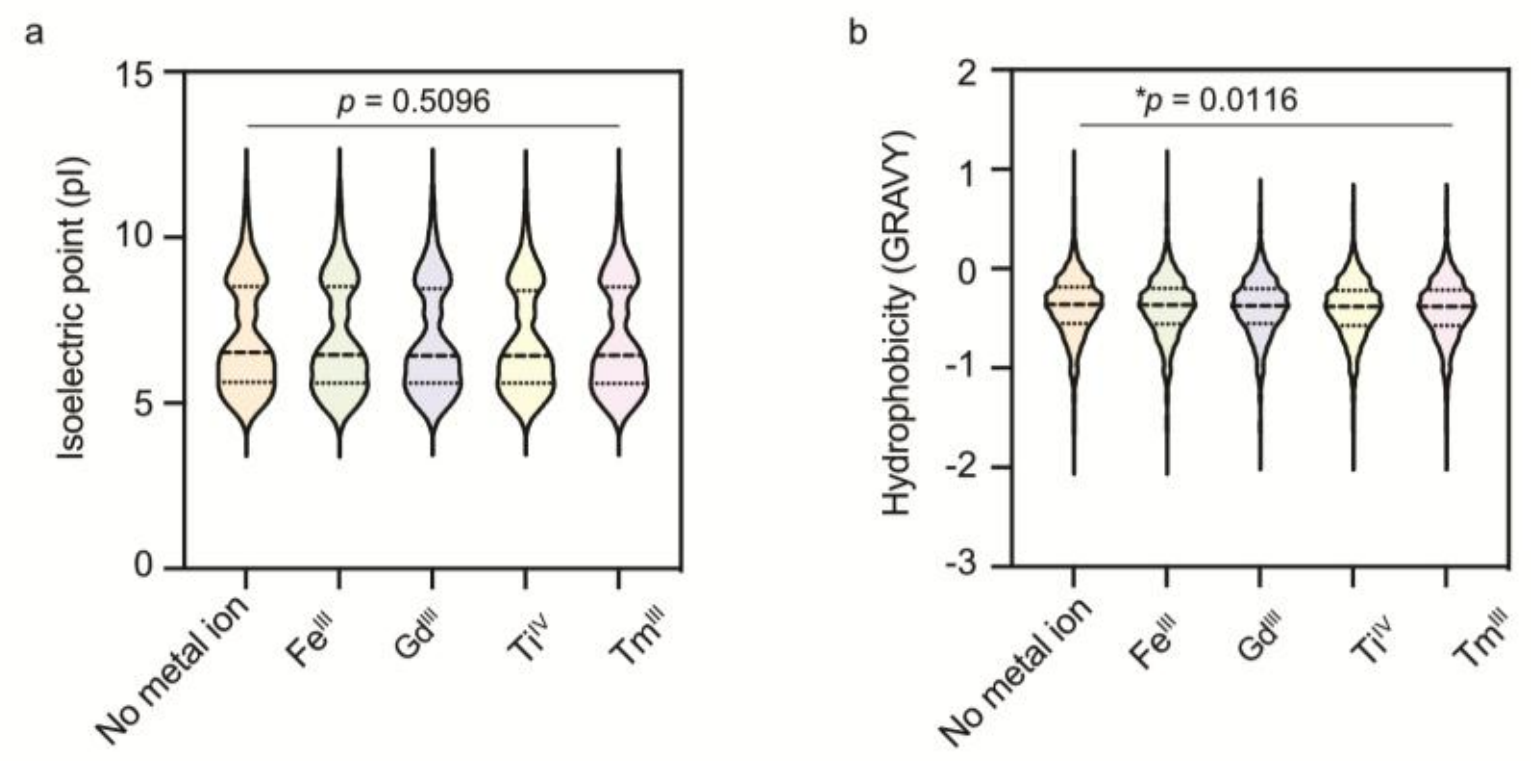

Supplementary Fig. 31 | Global physicochemical property distributions of plasma proteins adsorbed onto the surface of MPN NPs. a,b, Violin plots show the distributions of isoelectric point (**a**) and hydrophobicity (**b**) of proteins associated with the metal-free, TA/Fe^{III}-based, TA/Gd^{III}-based, TA/Ti^{IV}-based, and TA/Tm^{III}-based MPN NPs. Dashed lines denote median values and interquartile ranges. Proteins detected in at least one replicate were included in the analysis. Statistical significance was analyzed using one-way analysis of variance with Kruskal–Wallis test. The isoelectric point distributions were nearly identical across all groups (median ≈ 6.4; interquartile range = 5.6–8.5), indicating no significant formulation-dependent shift in overall protein charge characteristics. Similarly, hydrophobicity, assessed by the grand average of hydropathy (GRAVY), displayed largely overlapping distributions among formulations. Median GRAVY values were uniformly negative, indicating that the protein coronas were predominantly composed of hydrophilic proteins across all conditions. These values indicate that global protein hydrophobicity is largely maintained across the examined MPN NPs and is less likely to represent a dominant factor underlying metal ion-mediated biological behaviors.

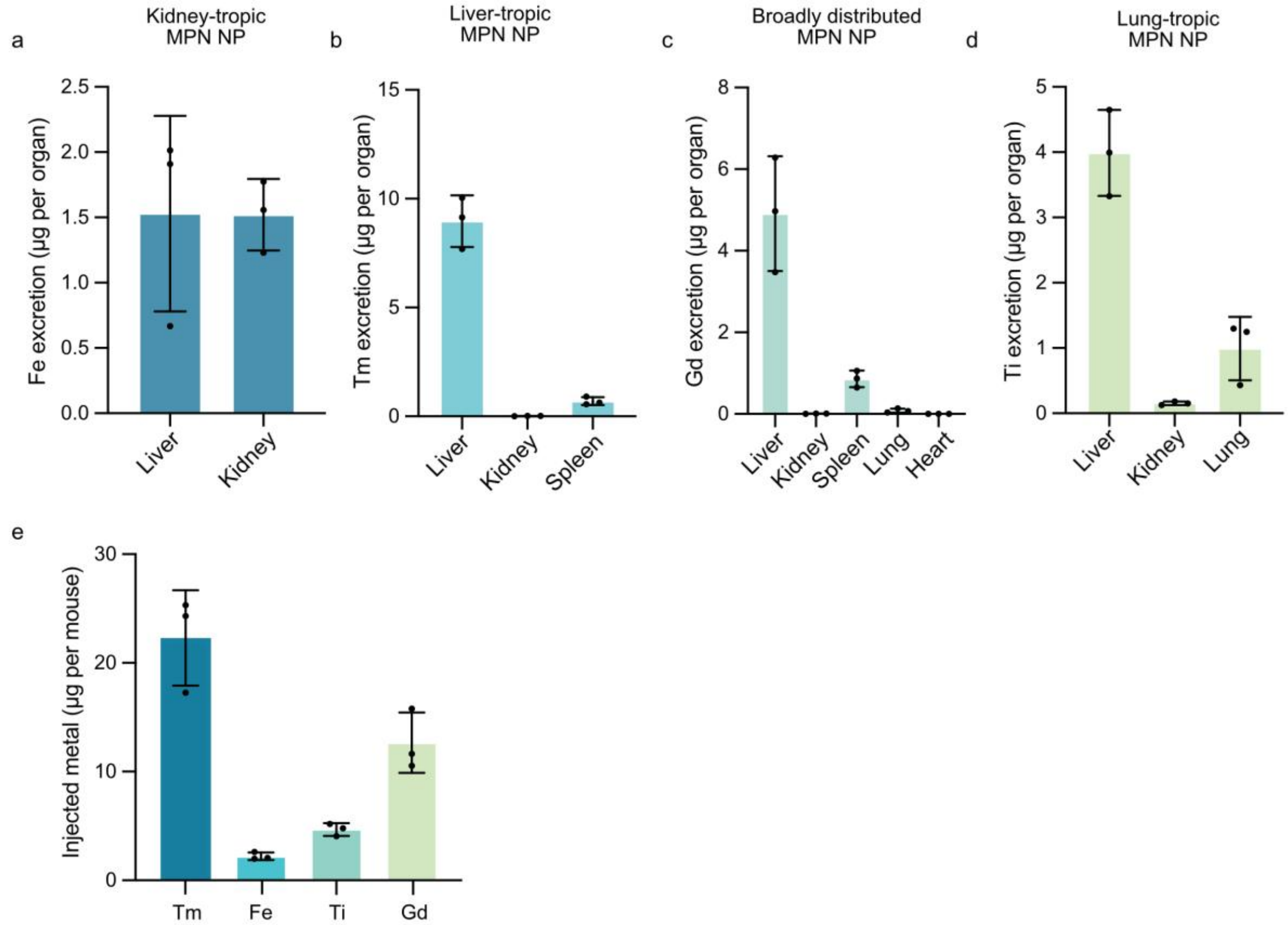

Supplementary Fig. 32 | Metal content analysis via ICP-MS. a–d, Retention of metal ions in harvested organs 7 days post-IV administration of different organ-tropic MPN NPs. **e,** Amount of metal ion introduced into mice. $n = 3$ mice per group; data are presented as mean ± SD. The dosage of NPs was 5 mg kg^{-1}. NP composition: 2 kDa PEG, BSA, and TA, and metal ions (Fe^{III}, Tm^{III}, Gd^{III}, or Ti^{IV}); metal ion-to-TA molar ratio is 2:1.

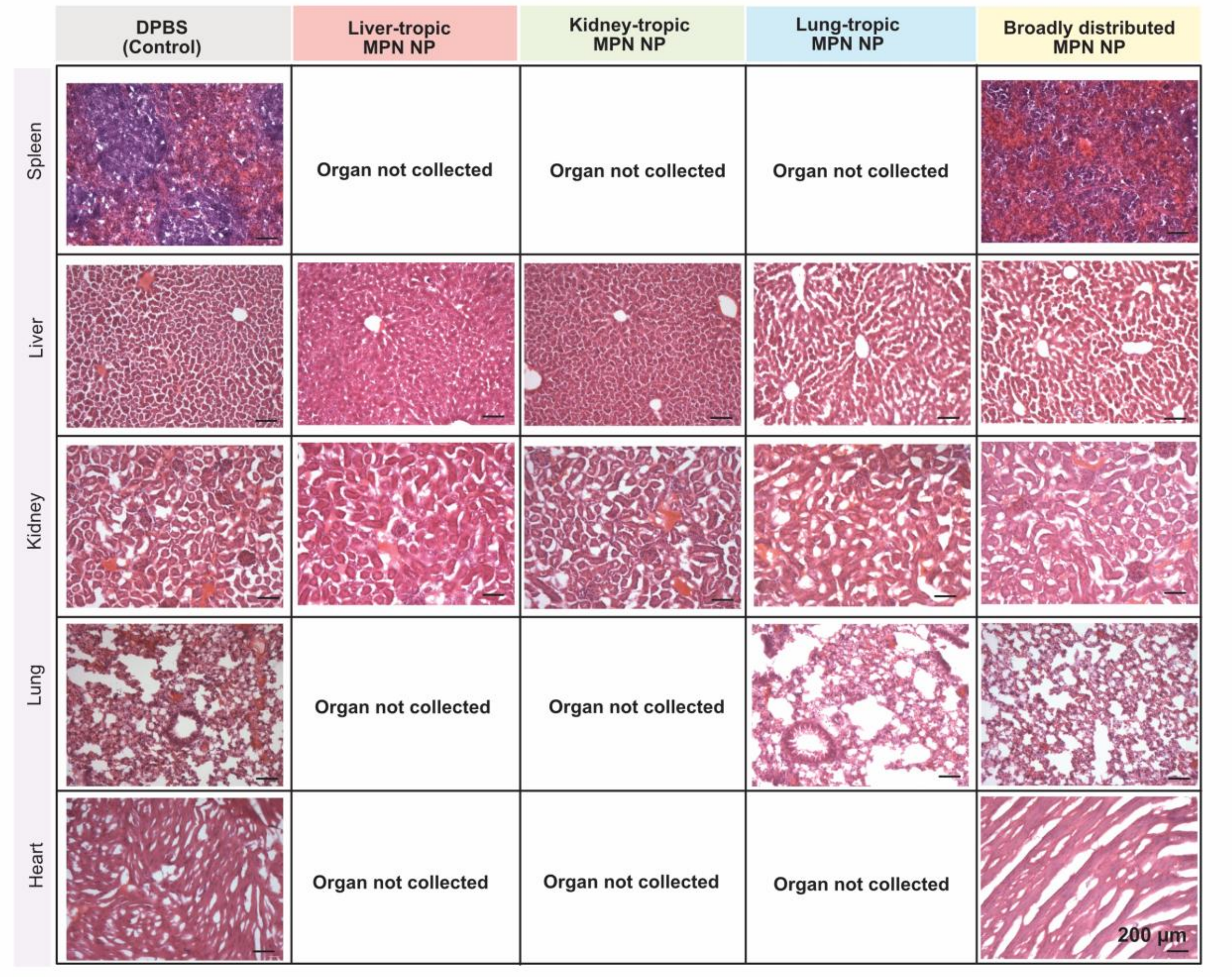

Supplementary Fig. 33 | Representative hematoxylin and eosin staining of sectioned tissues of mice treated with DPBS (negative control) or different organ-tropic TA-based MPN NPs after 7 days. Liver-tropic: metal-free and TA/Tm^{III}; kidney-tropic: TA/Fe^{III}; lung-tropic: TA/Ti^{IV}; broadly distributed: TA/Gd^{III}. Images are representative of n = 3 mice (for both DPBS and NP-treated groups).

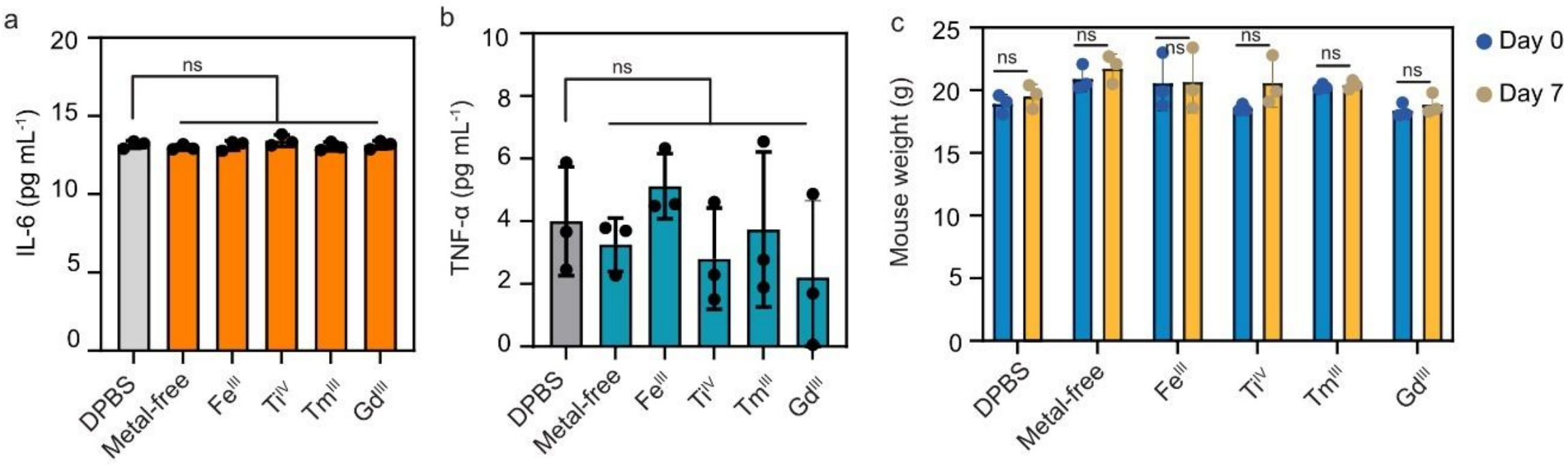

Supplementary Fig. 34 | Cytokine release profiles and changes in mouse body weight following IV administration of selected organ-tropic MPN NPs for each organ tropism. a,b, Levels of IL-6 (**a**) and TNF-α (**b**) in plasma collected from mice treated with DPBS (negative control) or different organ-tropic TA-based MPN NPs 7 days post-IV administration. **c,** Changes in mouse body weight 7 days after IV administration of DPBS or organ-tropic TA-based MPN NPs. n = 3 mice per group; and data are presented as mean ± SD. In (**c**), although the individual values in the Day 0 and Day 7 groups (for Ti^{IV}) do not overlap, the statistical analysis does not indicate a significant difference, possibly because of the limited sample size. The dosage of MPN NPs was 5 mg kg^{-1}. NP composition: 2 kDa PEG, BSA, and TA, with or without metal ions; metal ion-to-TA molar ratio is 2:1.

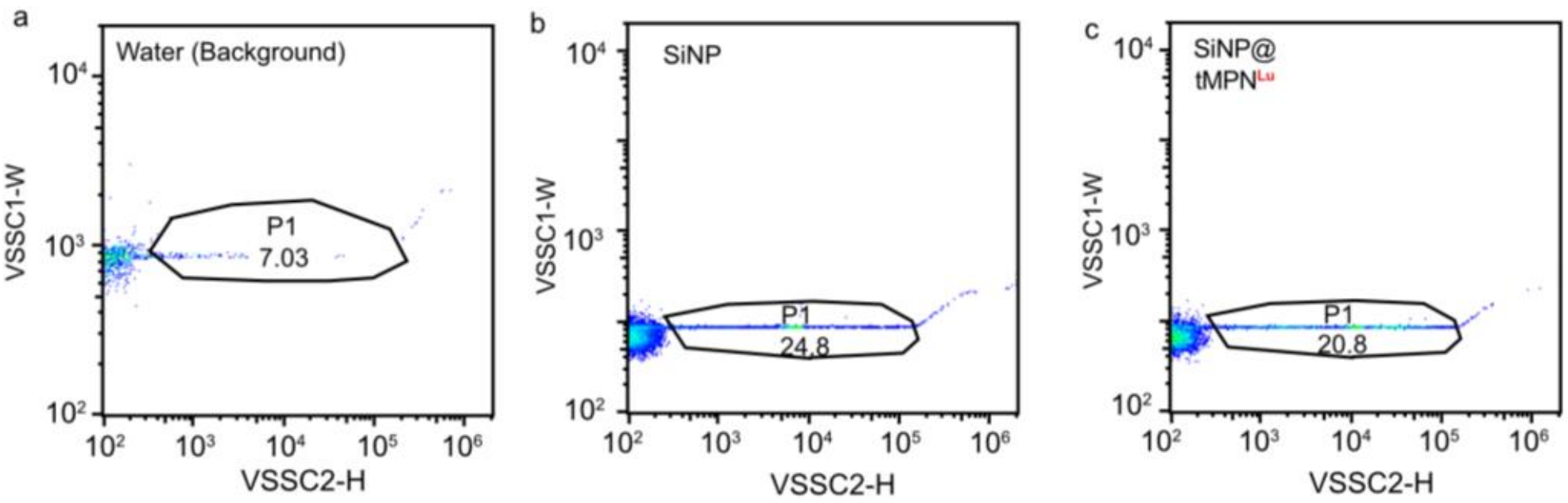

Supplementary Fig. 35 | Gating strategy for SiNP@$tMPN^{Lu}$ population evaluation. a–c, Representative gating strategies used in flow cytometry studies for the detection of SiNP (**b**) and SiNP@$tMPN^{Lu}$ (**c**) populations via violet side scatter (VSSC) with water (**a**) as the background. The composition of the $tMPN^{Lu}$ coating was 20 kDa PEG, BSA, TA, and Ti^{IV}.

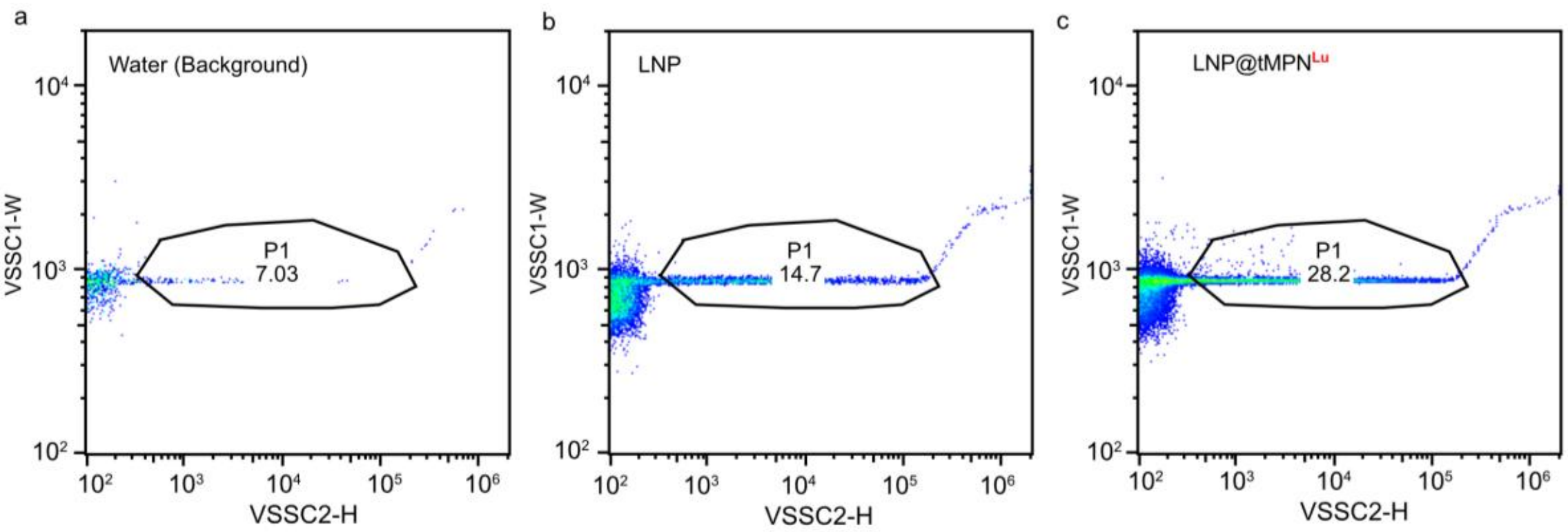

Supplementary Fig. 36 | Gating strategy for LNP@tMPN^{Lu} population evaluation. a–c, Representative gating strategies used in flow cytometry studies for the detection of LNP (**b**) and LNP@tMPN^{Lu} (**c**) populations via VSSC with water (**a**) as the background. The composition of the tMPN^{Lu} coating was 20 kDa PEG, BSA, TA, and Co^{II}.

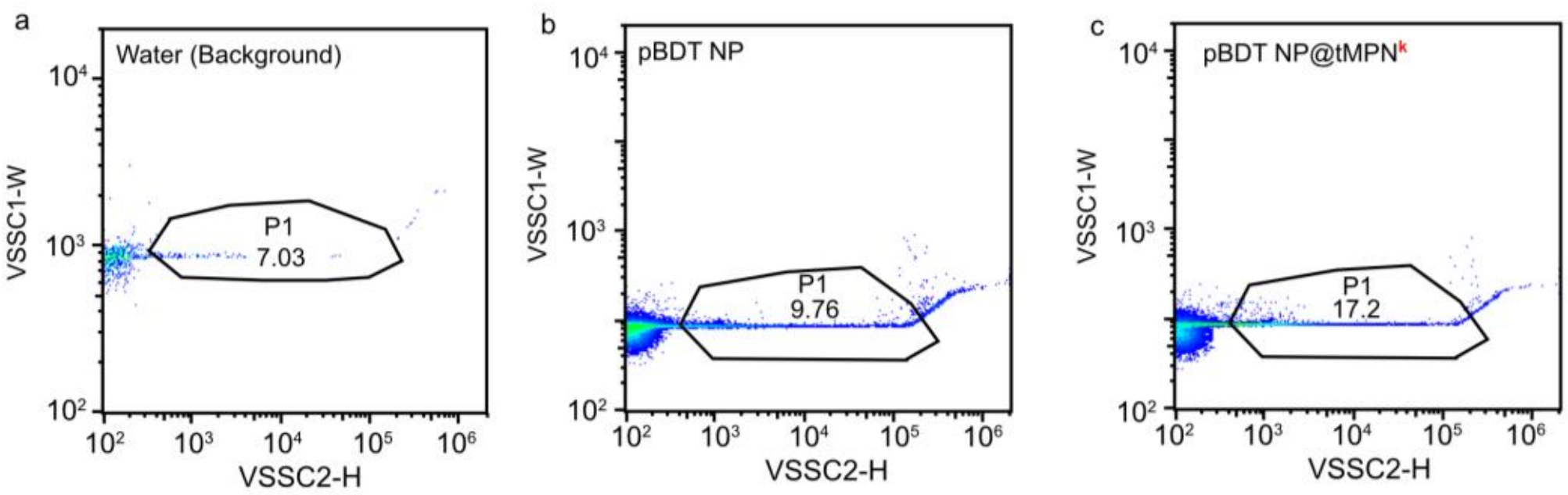

Supplementary Fig. 37 | Gating strategy for pBDT NP@tMPN^{K} population evaluation. a–c, Representative gating strategies used in flow cytometry studies for the detection of pBDT NP (**b**) and pBDT NP@tMPN^{K} (**c**) populations via VSSC with water (**a**) as the background. The composition of the tMPN^{K} coating was 20 kDa PEG, BSA, TA, and Cr^{III}.

Supplementary Tables

Supplementary Table 1 | Characterization of (Ti^{IV})MPN NPs assembled using different Ti^{IV}-to-TA molar ratios

Ti^{IV}-to-TA molar ratio	Size (diameter; nm)[a]	PDI	ζ-Potential (mV)[b]
0:1	211 ± 93	0.168	–56 ± 5
0.1:1	150 ± 45	0.057	–53 ± 7
0.5:1	94 ± 24	0.061	–46 ± 15
1:1	86 ± 28	0.162	–43 ± 4
2:1	61 ± 18	0.103	–33 ± 6

[a]Size reduction with increasing Ti^{IV}-to-TA molar ratio was likely due to increased cross-linking density. [b]Although the ζ-potential of MPN NPs became less negative with a higher proportion of metal salts, particles remained overall negatively charged. NP composition: 2 kDa PEG, BSA, TA, with or without Ti^{IV}. PDI, polydispersity index. n = 3 independent experiments, and the data are presented as mean ± SD.

Supplementary Table 2 | Characterization and primary organ tropism of TA-based organ-tropic MPN NPs assembled with or without metal ions

Metal ion	Size (diameter; nm)	PDI	ζ-Potential (mV)	Organ tropism
**Metal free**	211 ± 93	0.168	–56 ± 5	Liver-tropic
**Co^{II}**	61 ± 18	0.109	–31 ± 4	Lung-tropic
**Cu^{II}**	90 ± 39	0.107	–43 ± 4	Metabolic organ-tropic
**Zn^{II}**	206 ± 80	0.103	–33 ± 6	Kidney-tropic
**Mn^{II}**	126 ± 40	0.098	–36 ± 6	Kidney-tropic
**Fe^{III}**	64 ± 20	0.108	–39 ± 6	Kidney-tropic
**Cr^{III}**	74 ± 24	0.177	–43 ± 4	Kidney-tropic
**Tm^{III}**	81 ± 24	0.082	–22 ± 4	Liver-tropic
**Bi^{III}**	230 ± 65	0.149	–25 ± 4	Metabolic organ-tropic
**Gd^{III}**	82 ± 25	0.105	–21 ± 7	Broad distribution
**Zr^{IV}**	54 ± 14	0.093	–22 ± 5	Lung-tropic
**Ir^{IV}**	108 ± 56	0.179	–55 ± 6	Lung-tropic
**Pt^{IV}**	98 ± 48	0.201	–51 ± 7	Broad distribution
**Ta^{V}**	66 ± 25	0.219	–28 ± 4	Lung-tropic
**Nb^{V}**	50 ± 6	0.225	–34 ± 5	Broad distribution
**W^{VI}**	143 ± 64	0.179	–29 ± 5	Lung-tropic
**Ti^{IV}**	61 ± 18	0.103	–33 ± 6	Lung-tropic

NP composition: 2 kDa PEG, BSA, TA, and metal ions listed in the table. n = 3 independent experiments, and the data are presented as mean ± SD.

Supplementary Table 3 | Summary of intrinsic features of metals involved in the assembly of organ-tropic TA-based MPN NPs[4]

Metal	Oxidation state	Atomic number	Ionic radius (Å)	Atomic radius (Å)[a]	Atomic weight	Electro-negativity
**Cu**	2	29	0.73	1.35	63.55	1.90
**Zn**	2	30	0.74	1.35	65.38	1.65
**Co**	2	27	0.70	1.35	58.93	1.88
**Fe**	3	26	0.60	1.40	55.85	1.55
**Cr**	3	24	0.62	1.40	51.99	1.83
**Tm**	3	69	0.97	1.75	168.93	1.66
**Bi**	3	83	1.03	1.60	208.98	1.62
**Gd**	3	64	1.02	1.80	157.25	2.02
**Zr**	4	40	0.83	1.55	91.22	1.20
**Ti**	4	22	0.69	1.40	47.87	1.33
**Ir**	4	77	0.63	1.35	192.22	1.54
**Pt**	4	78	0.77	1.35	195.08	2.28
**Ta**	5	73	0.69	1.45	180.95	1.50
**Nb**	5	41	0.64	1.45	92.91	1.60
**W**	6	74	0.65	1.35	183.84	2.36

[a]Empirical atomic radii were used.

Supplementary Table 4 | Average abundance and physiological function of top 40 proteins detected in neat C57BL/6 plasma[a]

Protein	Abundance (%)	Functional class
**ALBUMIN**	25.55	Other
**APOLIPOPROTEIN A-I**	10.30	Apolipoprotein
**HEMOGLOBIN SUBUNIT BETA-1**	7.58	Other
**HEMOGLOBIN SUBUNIT ALPHA**	5.62	Other
**SEROTRANSFERRIN**	5.46	Other
**ALPHA-1-ANTITRYPSIN 1-4**	4.03	Acute phase protein
**HEMOPEXIN**	2.84	Other
**ALPHA-1-ANTITRYPSIN 1-5**	2.43	Acute phase protein
**PREGNANCY ZONE PROTEIN**	2.35	Immune protein
**SERINE PROTEASE INHIBITOR A3M**	2.19	Other
**TRANSTHYRETIN**	2.12	Other
**SERINE PROTEASE INHIBITOR A3K**	1.46	Immune protein
**HAPTOGLOBIN**	1.38	Other
**FIBRINOGEN BETA CHAIN**	1.18	Coagulation protein
**MAJOR URINARY PROTEIN 18**	0.96	Other
**IMMUNOGLOBULIN KAPPA CONSTANT**	0.91	Immune protein
**MURINOGLOBULIN-1**	0.88	Other
**FIBRINOGEN GAMMA CHAIN**	0.85	Coagulation protein
**CARBOXYLESTERASE 1C**	0.83	Other
**ALPHA-2-HS-GLYCOPROTEIN**	0.78	Other
**APOLIPOPROTEIN A-IV**	0.76	Apolipoprotein
**ALPHA-1-ANTITRYPSIN 1-1**	0.76	Acute phase protein
**APOLIPOPROTEIN C-III**	0.75	Apolipoprotein
**APOLIPOPROTEIN A-II**	0.69	Apolipoprotein
**FIBRINOGEN ALPHA CHAIN**	0.68	Coagulation protein
**IMMUNOGLOBULIN HEAVY CONSTANT GAMMA 2B**	0.66	Immune protein
**KININOGEN-1**	0.57	Coagulation protein
**COMPLEMENT C3**	0.53	Complement protein
**APOLIPOPROTEIN C-I**	0.52	Apolipoprotein
**APOLIPOPROTEIN E**	0.46	Apolipoprotein
**VITAMIN D-BINDING PROTEIN**	0.45	Other
**IG GAMMA-2A CHAIN C REGION SECRETED FORM**	0.45	Immune protein
**SERPIN A11**	0.44	Immune protein
**IMMUNOGLOBULIN HEAVY CONSTANT MU**	0.42	Immune protein
**ALPHA-1-ANTITRYPSIN 1-2**	0.41	Acute phase protein
**PLASMINOGEN**	0.38	Coagulation protein
**BETA-2-GLYCOPROTEIN 1**	0.35	Other
**H-2 CLASS I HISTOCOMPATIBILITY ANTIGEN, Q10 ALPHA CHAIN**	0.29	Immune protein
**ANTITHROMBIN-III**	0.29	Coagulation protein
**CLUSTERIN**	0.25	Apolipoprotein

[a] n = 4 independent replicates of a single pooled plasma sample.

Supplementary Table 5 | Average abundance and physiological function of top 40 proteins detected in plasma protein corona adsorbed onto metal-free MPN NPs (liver-tropic formulation)[a]

Protein	Abundance (%)	Functional class
**APOLIPOPROTEIN A-I**	17.85	Apolipoprotein
**FIBRINOGEN BETA CHAIN**	16.94	Coagulation protein
**FIBRINOGEN ALPHA CHAIN**	16.17	Coagulation protein
**FIBRINOGEN GAMMA CHAIN**	13.94	Coagulation protein
**HEMOGLOBIN SUBUNIT BETA-1**	4.36	Other
**HEMOGLOBIN SUBUNIT ALPHA**	3.61	Other
**ALBUMIN**	2.53	Other
**APOLIPOPROTEIN E**	1.82	Apolipoprotein
**APOLIPOPROTEIN C-I**	1.52	Apolipoprotein
**GELSOLIN**	1.36	Other
**FIBRONECTIN**	1.12	Acute phase protein
**LYSOZYME C-2**	0.89	Other
**COMPLEMENT C3**	0.78	Complement protein
**APOLIPOPROTEIN A-IV**	0.78	Apolipoprotein
**IMMUNOGLOBULIN KAPPA CONSTANT**	0.62	Immune protein
**APOLIPOPROTEIN C-III**	0.57	Apolipoprotein
**INTER ALPHA-TRYPSIN INHIBITOR, HEAVY CHAIN 4**	0.51	Acute phase protein
**SERUM AMYLOID A-1 PROTEIN**	0.51	Acute phase protein
**IG GAMMA-3 CHAIN C REGION**	0.48	Immune protein
**COMPLEMENT COMPONENT C8 GAMMA CHAIN**	0.40	Complement protein
**PLASMINOGEN**	0.39	Coagulation protein
**VITRONECTIN**	0.36	Other
**PREGNANCY ZONE PROTEIN**	0.36	Immune protein
**CLUSTERIN**	0.35	Apolipoprotein
**MANNOSE-BINDING PROTEIN A**	0.34	Complement protein
**IMMUNOGLOBULIN HEAVY CONSTANT MU**	0.34	Immune protein
**IG GAMMA-2A CHAIN C REGION SECRETED FORM**	0.34	Immune protein
**COMPLEMENT COMPONENT C8 BETA CHAIN**	0.30	Complement protein
**PLASMA PROTEASE C1 INHIBITOR**	0.30	Other
**SERUM AMYLOID A-4 PROTEIN**	0.30	Acute phase protein
**COAGULATION FACTOR XIII A CHAIN**	0.27	Coagulation protein
**COMPLEMENT C4-B**	0.27	Complement protein
**COMPLEMENT FACTOR H**	0.26	Complement protein
**ALPHA-2-HS-GLYCOPROTEIN**	0.26	Other
**SEROTRANSFERRIN**	0.25	Other
**COAGULATION FACTOR XIII B CHAIN**	0.24	Coagulation protein
**SERUM AMYLOID A-2 PROTEIN**	0.21	Acute phase protein
**COMPLEMENT C5**	0.20	Complement protein
**ALPHA-1-ANTITRYPSIN 1-2**	0.19	Acute phase protein
**ANTITHROMBIN-III**	0.18	Coagulation protein

[a]n = 4 independent replicates of metal-free MPN NPs, each incubated with aliquots from the same pooled mouse plasma sample.

Supplementary Table 6 | Average abundance and physiological function of the top 40 proteins detected in plasma protein corona adsorbed onto TA/Tm^{III}-based MPN NPs (liver-tropic formulation)[a]

Protein	Abundance (%)	Functional class
**FIBRINOGEN BETA CHAIN**	13.24	Coagulation protein
**FIBRINOGEN GAMMA CHAIN**	11.86	Coagulation protein
**FIBRINOGEN ALPHA CHAIN**	11.18	Coagulation protein
**APOLIPOPROTEIN C-I**	11.02	Apolipoprotein
**ALBUMIN**	7.67	Other
**INTER ALPHA-TRYPSIN INHIBITOR, HEAVY CHAIN 4**	7.29	Acute phase protein
**CLUSTERIN**	6.83	Coagulation protein
**APOLIPOPROTEIN E**	6.56	Apolipoprotein
**GELSOLIN**	5.13	Other
**APOLIPOPROTEIN A-I**	3.62	Apolipoprotein
**SERUM AMYLOID A-1 PROTEIN**	1.64	Acute phase protein
**HEMOGLOBIN SUBUNIT ALPHA**	1.31	Other
**HEMOGLOBIN SUBUNIT BETA-1**	1.24	Other
**ALPHA-2-HS-GLYCOPROTEIN**	0.75	Other
**PLASMA PROTEASE C1 INHIBITOR**	0.74	Other
**LYSOZYME C-2**	0.64	Other
**FIBRONECTIN**	0.61	Acute phase protein
**ANTITHROMBIN-III**	0.57	Coagulation protein
**SERUM AMYLOID A-2 PROTEIN**	0.52	Acute phase protein
**APOLIPOPROTEIN C-III**	0.46	Apolipoprotein
**MANNOSE-BINDING PROTEIN A**	0.42	Complement protein
**GLYCERALDEHYDE-3-PHOSPHATE DEHYDROGENASE**	0.33	Other
**ELONGATION FACTOR 1-ALPHA 1**	0.22	Other
**SELENOPROTEIN P**	0.21	Other
**GEM-ASSOCIATED PROTEIN 5**	0.19	Other
**ALPHA-1-ANTITRYPSIN 1-2**	0.19	Acute phase protein
**APOLIPOPROTEIN C-IV**	0.18	Apolipoprotein
**COAGULATION FACTOR XII**	0.18	Coagulation protein
**APOLIPOPROTEIN A-II**	0.17	Apolipoprotein
**KININOGEN-1**	0.17	Coagulation protein
**APOLIPOPROTEIN A-IV**	0.15	Apolipoprotein
**PLASMINOGEN**	0.11	Coagulation protein
**VITRONECTIN**	0.10	Other
**ALPHA-2-ANTIPLASMIN**	0.10	Coagulation protein
**COAGULATION FACTOR XIII A CHAIN**	0.10	Coagulation protein
**PREGNANCY ZONE PROTEIN**	0.09	Immune protein
**COAGULATION FACTOR XIII B CHAIN**	0.09	Coagulation protein
**LIPOPOLYSACCHARIDE-BINDING PROTEIN**	0.09	Acute phase protein
**COMPLEMENT C3**	0.09	Complement protein
**CYSTATIN-C**	0.09	Other

[a]n = 4 independent replicates of TA/Tm^{III}-based MPN NPs, each incubated with aliquots from the same pooled mouse plasma sample.

Supplementary Table 7 | Average abundance and physiological function of the top 40 proteins detected in plasma protein corona adsorbed onto TA/Ti^{IV}-based MPN NPs (lung-tropic formulation)[a]

Protein	Abundance (%)	Functional class
**FIBRINOGEN BETA CHAIN**	12.42	Coagulation protein
**FIBRINOGEN GAMMA CHAIN**	12.38	Coagulation protein
**FIBRINOGEN ALPHA CHAIN**	11.85	Coagulation protein
**APOLIPOPROTEIN E**	7.96	Apolipoprotein
**APOLIPOPROTEIN C-I**	7.18	Apolipoprotein
**GELSOLIN**	6.52	Other
**CLUSTERIN**	6.22	Coagulation protein
**INTER ALPHA-TRYPSIN INHIBITOR, HEAVY CHAIN 4**	5.61	Immune protein
**APOLIPOPROTEIN A-I**	4.41	Apolipoprotein
**ALBUMIN**	2.43	Other
**HEMOGLOBIN SUBUNIT BETA-1**	2.16	Other
**ALPHA-2-HS-GLYCOPROTEIN**	1.85	Other
**HEMOGLOBIN SUBUNIT ALPHA**	1.52	Other
**PLASMA PROTEASE C1 INHIBITOR**	1.37	Other
**FIBRONECTIN**	1.10	Acute phase protein
**ANTITHROMBIN-III**	1.08	Coagulation protein
**MANNOSE-BINDING PROTEIN A**	0.60	Complement protein
**SERUM AMYLOID A-1 PROTEIN**	0.58	Acute phase protein
**COMPLEMENT C3**	0.52	Complement protein
**LYSOZYME C-2**	0.45	Other
**GLYCERALDEHYDE-3-PHOSPHATE DEHYDROGENASE**	0.41	Other
**KININOGEN-1**	0.38	Coagulation protein
**PLASMINOGEN**	0.37	Coagulation protein
**APOLIPOPROTEIN C-III**	0.36	Apolipoprotein
**VITRONECTIN**	0.29	Other
**ALPHA-1-ANTITRYPSIN 1-2**	0.23	Acute phase protein
**HEMOPEXIN**	0.23	Other
**HISTIDINE-RICH GLYCOPROTEIN**	0.22	Coagulation protein
**ELONGATION FACTOR 1-ALPHA 1**	0.21	Other
**SERUM AMYLOID A-2 PROTEIN**	0.21	Acute phase protein
**COAGULATION FACTOR XII**	0.20	Coagulation protein
**IMMUNOGLOBULIN KAPPA CONSTANT**	0.19	Immune protein
**IG GAMMA-3 CHAIN C REGION**	0.18	Immune protein
**THROMBOSPONDIN-1**	0.18	Coagulation protein
**APOLIPOPROTEIN A-IV**	0.18	Apolipoprotein
**COMPLEMENT FACTOR H**	0.18	Complement protein
**SELENOPROTEIN P**	0.17	Other
**ALPHA-2-ANTIPLASMIN**	0.17	Coagulation protein
**COMPLEMENT C4-B**	0.17	Complement protein
**SULFHYDRYL OXIDASE 1**	0.17	Other

[a]n = 4 independent replicates of TA/Ti^{IV}-based MPN NPs, each incubated with aliquots from the same pooled mouse plasma sample.

Supplementary Table 8 | Average abundance and physiological function of the top 40 proteins detected in plasma protein corona adsorbed onto TA/Fe^{III}-based MPN NPs (kidney-tropic formulation)[a]

Protein	Abundance (%)	Functional class
**FIBRINOGEN BETA CHAIN**	19.86	Coagulation protein
**FIBRINOGEN GAMMA CHAIN**	18.30	Coagulation protein
**FIBRINOGEN ALPHA CHAIN**	13.75	Coagulation protein
**INTER ALPHA-TRYPSIN INHIBITOR, HEAVY CHAIN 4**	6.21	Acute phase protein
**GELSOLIN**	5.64	Other
**APOLIPOPROTEIN E**	4.63	Apolipoprotein
**ALBUMIN**	4.13	Other
**HEMOGLOBIN SUBUNIT BETA-1**	3.62	Other
**APOLIPOPROTEIN A-I**	3.36	Apolipoprotein
**HEMOGLOBIN SUBUNIT ALPHA**	3.36	Other
**CLUSTERIN**	2.89	Apolipoprotein
**APOLIPOPROTEIN C-I**	1.18	Apolipoprotein
**FIBRONECTIN**	0.74	Acute phase protein
**SERUM AMYLOID A-1 PROTEIN**	0.73	Acute phase protein
**HISTIDINE-RICH GLYCOPROTEIN**	0.63	Coagulation protein
**APOLIPOPROTEIN C-III**	0.60	Apolipoprotein
**GLYCERALDEHYDE-3-PHOSPHATE DEHYDROGENASE**	0.52	Other
**ALPHA-2-HS-GLYCOPROTEIN**	0.51	Other
**ANTITHROMBIN-III**	0.41	Coagulation protein
**ALPHA-1-ANTITRYPSIN 1-2**	0.41	Acute phase protein
**KININOGEN-1**	0.36	Coagulation protein
**SELENOPROTEIN P**	0.31	Other
**SERUM AMYLOID A-2 PROTEIN**	0.30	Acute phase protein
**COAGULATION FACTOR XII**	0.26	Coagulation protein
**ELONGATION FACTOR 1-ALPHA 1**	0.23	Other
**SERINE PROTEASE INHIBITOR A3M**	0.19	Other
**COMPLEMENT C3**	0.16	Complement protein
**APOLIPOPROTEIN A-IV**	0.15	Apolipoprotein
**MANNOSE-BINDING PROTEIN A**	0.15	Complement protein
**LYSOZYME C-2**	0.15	Other
**PLASMA KALLIKREIN**	0.15	Coagulation protein
**PREGNANCY ZONE PROTEIN**	0.13	Immune protein
**VITRONECTIN**	0.12	Other
**ALPHA-2-ANTIPLASMIN**	0.12	Coagulation protein
**PLASMA PROTEASE C1 INHIBITOR**	0.11	Other
**ALPHA-1-ANTITRYPSIN 1-3**	0.10	Acute phase protein
**TRANSTHYRETIN**	0.10	Other
**HISTONE H2A TYPE 2-C**	0.10	Other
**HISTONE H4**	0.10	Other
**EXTRACELLULAR MATRIX PROTEIN 1**	0.09	Other

[a] n = 3 independent replicates of TA/Fe^{III}-based MPN NPs, each incubated with aliquots from the same pooled mouse plasma sample.

Supplementary Table 9 | Average abundance and physiological function of the top 40 proteins detected in plasma protein corona adsorbed onto TA/Gd^{III}-based MPN NPs (formulation with broad distribution)[a]

Protein	Abundance (%)	Functional class
**FIBRINOGEN BETA CHAIN**	14.37	Coagulation protein
**FIBRINOGEN GAMMA CHAIN**	12.88	Coagulation protein
**FIBRINOGEN ALPHA CHAIN**	12.37	Coagulation protein
**ALBUMIN**	9.65	Other
**INTER ALPHA-TRYPSIN INHIBITOR, HEAVY CHAIN 4**	7.84	Acute phase protein
**CLUSTERIN**	7.84	Apolipoprotein
**APOLIPOPROTEIN C-I**	5.71	Apolipoprotein
**GELSOLIN**	5.64	Other
**APOLIPOPROTEIN E**	4.21	Apolipoprotein
**APOLIPOPROTEIN A-I**	3.10	Apolipoprotein
**SERUM AMYLOID A-1 PROTEIN**	1.88	Acute phase protein
**HEMOGLOBIN SUBUNIT BETA-1**	1.22	Other
**HEMOGLOBIN SUBUNIT ALPHA**	1.18	Other
**PLASMA PROTEASE C1 INHIBITOR**	0.94	Other
**ALPHA-2-HS-GLYCOPROTEIN**	0.72	Other
**SERUM AMYLOID A-2 PROTEIN**	0.62	Acute phase protein
**FIBRONECTIN**	0.62	Acute phase protein
**MANNOSE-BINDING PROTEIN A**	0.58	Complement protein
**ANTITHROMBIN-III**	0.55	Coagulation protein
**LYSOZYME C-2**	0.53	Other
**GLYCERALDEHYDE-3-PHOSPHATE DEHYDROGENASE**	0.37	Other
**APOLIPOPROTEIN C-III**	0.36	Apolipoprotein
**ALPHA-1-ANTITRYPSIN 1-2**	0.27	Acute phase protein
**GEM-ASSOCIATED PROTEIN 5**	0.22	Other
**ELONGATION FACTOR 1-ALPHA 1**	0.21	Other
**SELENOPROTEIN P**	0.21	Other
**APOLIPOPROTEIN C-IV**	0.16	Apolipoprotein
**COAGULATION FACTOR XII**	0.15	Coagulation protein
**KININOGEN-1**	0.15	Coagulation protein
**APOLIPOPROTEIN A-II**	0.14	Apolipoprotein
**PLASMINOGEN**	0.14	Coagulation protein
**COAGULATION FACTOR XIII B CHAIN**	0.13	Coagulation protein
**ALPHA-2-ANTIPLASMIN**	0.11	Coagulation protein
**PREGNANCY ZONE PROTEIN**	0.11	Immune protein
**COAGULATION FACTOR XIII A CHAIN**	0.11	Coagulation protein
**TRANSTHYRETIN**	0.11	Other
**VITRONECTIN**	0.10	Other
**LIPOPOLYSACCHARIDE-BINDING PROTEIN**	0.10	Acute phase protein
**SERINE PROTEASE INHIBITOR A3M**	0.10	Other
**APOLIPOPROTEIN A-IV**	0.10	Apolipoprotein

[a]n = 4 independent replicates of TA/Gd^{III}-based MPN NPs, each incubated with aliquots from the same pooled mouse plasma sample.

Supplementary Table 10 | Average abundance and physiological function of the top 40 proteins detected in plasma protein corona adsorbed onto CAT/Ti^{IV}-based MPN NPs^{a}

Protein	Abundance (%)	Functional class
**ALBUMIN**	39.12	Others
**APOLIPOPROTEIN A-I**	14.40	Apolipoprotein
**ALPHA-1-ANTITRYPSIN 1-4**	6.66	Acute phase protein
**SEROTRANSFERRIN**	4.51	Other
**TRANSTHYRETIN**	2.84	Other
**SERINE PROTEASE INHIBITOR A3M**	2.13	Other
**PREGNANCY ZONE PROTEIN**	2.10	Immunoprotein
**HEMOPEXIN**	1.72	Acute phase protein
**FIBRINOGEN BETA CHAIN**	2.49	Coagulation protein
**ALPHA-1-ANTITRYPSIN 1-5**	1.41	Acute phase protein
**FIBRINOGEN ALPHA CHAIN**	1.91	Coagulation protein
**CARBOXYLESTERASE 1C**	1.26	Other
**FIBRINOGEN GAMMA CHAIN**	1.89	Coagulation protein
**ALPHA-2-HS-GLYCOPROTEIN**	1.23	Other
**APOLIPOPROTEIN C-I**	1.07	Apolipoprotein
**APOLIPOPROTEIN A-II**	0.88	Apolipoprotein
**IMMUNOGLOBULIN KAPPA CONSTANT**	1.06	Immunoprotein
**SERINE PROTEASE INHIBITOR A3K**	1.23	Other
**ALPHA-1-ANTITRYPSIN 1-1**	1.09	Acute phase protein
**APOLIPOPROTEIN A-IV**	0.94	Apolipoprotein
**APOLIPOPROTEIN E**	0.82	Apolipoprotein
**ALPHA-1-ANTITRYPSIN 1-2**	0.67	Acute phase protein
**MURINOGLOBULIN-1**	0.69	Other
**COMPLEMENT C3**	0.73	Complement protein
**IMMUNOGLOBULIN HEAVY CONSTANT GAMMA 2B**	0.74	Immunoprotein
**KININOGEN-1**	0.56	Coagulation protein
**IMMUNOGLOBULIN HEAVY CONSTANT MU**	0.67	Immunoprotein
**BETA-2-GLYCOPROTEIN 1**	0.50	Other
**PLASMINOGEN**	0.42	Coagulation protein
**APOLIPOPROTEIN C-III**	1.21	Apolipoprotein
**VITAMIN D-BINDING PROTEIN**	0.35	Other
**GELSOLIN**	0.41	Other
**IMMUNOGLOBULIN HEAVY CONSTANT GAMMA 2C (FRAGMENT)**	0.35	Immunoprotein
**MAJOR URINARY PROTEIN 12**	0.27	Other
**ANTITHROMBIN-III**	0.31	Coagulation protein
**IMMUNOGLOBULIN HEAVY CONSTANT GAMMA 3 (FRAGMENT)**	0.33	Immunoprotein
**CLUSTERIN**	0.29	Apolipoprotein
**HISTONE H4**	0.22	Other
**PROTHROMBIN**	0.27	Coagulation protein
**ALPHA-2-ANTIPLASMIN**	0.24	Coagulation protein

^{a}n = 4 independent replicates of CAT/Ti^{IV}-based MPN NPs, each incubated with aliquots from the same pooled mouse plasma sample.

Supplementary Table 11 | Average abundance and physiological function of the top 40 proteins detected in plasma protein corona adsorbed onto GA/Ti^{IV}-based MPN NPs^{a}

Protein	Abundance (%)	Functional class
**ALBUMIN**	33.57	Other
**APOLIPOPROTEIN A-I**	18.72	Apolipoprotein
**ALPHA-1-ANTITRYPSIN 1-4**	7.75	Acute phase protein
**SEROTRANSFERRIN**	4.48	Other
**TRANSTHYRETIN**	3.61	Other
**SERINE PROTEASE INHIBITOR A3M**	2.33	Other
**PREGNANCY ZONE PROTEIN**	2.44	Immunoprotein
**ALPHA-1-ANTITRYPSIN 1-5**	2.14	Acute phase protein
**FIBRINOGEN BETA CHAIN**	1.97	Coagulation protein
**SERINE PROTEASE INHIBITOR A3K**	1.71	Other
**HEMOPEXIN**	1.64	Acute phase protein
**CARBOXYLESTERASE 1C**	1.58	Other
**ALPHA-1-ANTITRYPSIN 1-1**	1.31	Acute phase protein
**FIBRINOGEN GAMMA CHAIN**	1.67	Coagulation protein
**APOLIPOPROTEIN C-III**	1.14	Apolipoprotein
**IMMUNOGLOBULIN KAPPA CONSTANT**	1.18	Immunoprotein
**APOLIPOPROTEIN C-I**	1.05	Apolipoprotein
**APOLIPOPROTEIN A-IV**	1.00	Apolipoprotein
**APOLIPOPROTEIN E**	0.83	Apolipoprotein
**ALPHA-1-ANTITRYPSIN 1-2**	0.81	Acute phase protein
**MURINOGLOBULIN-1**	0.81	Other
**ALPHA-2-HS-GLYCOPROTEIN**	0.88	Other
**IMMUNOGLOBULIN HEAVY CONSTANT GAMMA 2B**	0.70	Immunoprotein
**COMPLEMENT C3**	0.80	Complement protein
**IMMUNOGLOBULIN HEAVY CONSTANT MU**	0.67	Immunoprotein
**APOLIPOPROTEIN A-II**	0.63	Apolipoprotein
**HISTONE H4**	0.65	Other
**KININOGEN-1**	0.54	Coagulation protein
**IMMUNOGLOBULIN HEAVY CONSTANT GAMMA 2C (FRAGMENT)**	0.36	Immunoprotein
**MAJOR URINARY PROTEIN 12**	0.36	Other
**ANTITHROMBIN-III**	0.39	Coagulation protein
**VITAMIN D-BINDING PROTEIN**	0.40	Other
**GELSOLIN**	0.34	Other
**IMMUNOGLOBULIN HEAVY CONSTANT GAMMA 3 (FRAGMENT)**	0.33	Immunoprotein
**CLUSTERIN**	0.31	Apolipoprotein
**ALPHA-2-ANTIPLASMIN**	0.26	Coagulation protein
**PLASMINOGEN**	0.33	Coagulation protein
**FIBRONECTIN**	0.32	Acute phase protein
**HISTONE H2A TYPE 2-A**	0.41	Other

$^{a}n = 4$ independent replicates of GA/Ti^{IV}-based MPN NPs, each incubated with aliquots from the same pooled mouse plasma sample.

Supplementary References

1 Zhou, J. *et al.* Particle engineering enabled by polyphenol-mediated supramolecular networks. *Nat. Commun.* **11**, 4804 (2020).

2 Zhou, J. *et al.* Robust and versatile coatings engineered via simultaneous covalent and noncovalent Interactions. *Angew. Chem. Int. Ed.* **60**, 20225–20230 (2021).

3 Iida, H., Takayanagi, K., Nakanishi, T. & Osaka, T. Synthesis of Fe_3O_4 nanoparticles with various sizes and magnetic properties by controlled hydrolysis. *J. Colloid Interface Sci.* **314**, 274–280 (2007).

4 Wang, Z. *et al.* Machine learning guided dopant selection for metal oxide-based photoelectrochemical water splitting: the case study of Fe_2O_3 and CuO. *Adv. Mater.* **34**, e2106776 (2022).
