# Supplementary material for: Selective organ deposition of nanoparticles directed by metal–organic network coatings
Source: Nat Commun. 2026 Sep 2;17:10440. doi: 10.1038/s41467-026-77181-0 (PMC13631259; doi:10.1038/s41467-026-77181-0)
Supplement: Supplementary file 2 [file 41467_2026_77181_MOESM2_ESM.pdf]

nature portfolio

Corresponding author(s): Frank Caruso

Last updated by author(s): Jul 20, 2026

Reporting Summary

Nature Portfolio wishes to improve the reproducibility of the work that we publish. This form provides structure for consistency and transparency in reporting. For further information on Nature Portfolio policies, see our Editorial Policies and the Editorial Policy Checklist.

Statistics

For all statistical analyses, confirm that the following items are present in the figure legend, table legend, main text, or Methods section.

n/a	Confirmed	
☐	☒	The exact sample size (*n*) for each experimental group/condition, given as a discrete number and unit of measurement
☐	☒	A statement on whether measurements were taken from distinct samples or whether the same sample was measured repeatedly
☐	☒	The statistical test(s) used AND whether they are one- or two-sided<br>*Only common tests should be described solely by name; describe more complex techniques in the Methods section.*
☒	☐	A description of all covariates tested
☐	☒	A description of any assumptions or corrections, such as tests of normality and adjustment for multiple comparisons
☐	☒	A full description of the statistical parameters including central tendency (e.g. means) or other basic estimates (e.g. regression coefficient) AND variation (e.g. standard deviation) or associated estimates of uncertainty (e.g. confidence intervals)
☐	☒	For null hypothesis testing, the test statistic (e.g. *F*, *t*, *r*) with confidence intervals, effect sizes, degrees of freedom and *P* value noted<br>*Give P values as exact values whenever suitable.*
☒	☐	For Bayesian analysis, information on the choice of priors and Markov chain Monte Carlo settings
☒	☐	For hierarchical and complex designs, identification of the appropriate level for tests and full reporting of outcomes
☐	☒	Estimates of effect sizes (e.g. Cohen's *d*, Pearson's *r*), indicating how they were calculated

Our web collection on statistics for biologists contains articles on many of the points above.

Software and code

Policy information about availability of computer code

Data collection	No custom algorithms or software was used.
Data analysis	No custom algorithms or software was used. We used Graph Pad Prism V.9 and V.10 for data analysis. For In vivo imaging system (IVIS) analysis, we used Living Image Software (V4.4). For microscopy images analysis we used ImageJ V2.3.0. For linear regression and correlation analysis we used MATLAB_R2024b. For flow cytometry analysis, we used FlowJo V10. DIA data were analyzed using the direct DIA analysis workflow with default settings on the Spectronaut software (v. 19.4.241 104.62635) and against the Mus Musculus database (Uniprot, downloaded Mar 2025).

For manuscripts utilizing custom algorithms or software that are central to the research but not yet described in published literature, software must be made available to editors and reviewers. We strongly encourage code deposition in a community repository (e.g. GitHub). See the Nature Portfolio guidelines for submitting code & software for further information.

Data

Policy information about availability of data

All manuscripts must include a data availability statement. This statement should provide the following information, where applicable:

- Accession codes, unique identifiers, or web links for publicly available datasets
- A description of any restrictions on data availability
- For clinical datasets or third party data, please ensure that the statement adheres to our policy

All relevant data supporting the findings of this study are available within the paper and Supplementary Information. Source data are provided in this paper. The

mass spectrometry proteomics data generated in this study have been deposited in the ProteomeXchange Consortium via the PRIDE46 partner repository under accession code PXD077631. All data underlying this study are available from the corresponding author upon request.

Research involving human participants, their data, or biological material

Policy information about studies with human participants or human data. See also policy information about sex, gender (identity/presentation), and sexual orientation and race, ethnicity and racism.

Reporting on sex and gender	*Use the terms sex (biological attribute) and gender (shaped by social and cultural circumstances) carefully in order to avoid confusing both terms. Indicate if findings apply to only one sex or gender; describe whether sex and gender were considered in study design; whether sex and/or gender was determined based on self-reporting or assigned and methods used.* *Provide in the source data disaggregated sex and gender data, where this information has been collected, and if consent has been obtained for sharing of individual-level data; provide overall numbers in this Reporting Summary. Please state if this information has not been collected.* *Report sex- and gender-based analyses where performed, justify reasons for lack of sex- and gender-based analysis.*
Reporting on race, ethnicity, or other socially relevant groupings	*Please specify the socially constructed or socially relevant categorization variable(s) used in your manuscript and explain why they were used. Please note that such variables should not be used as proxies for other socially constructed/relevant variables (for example, race or ethnicity should not be used as a proxy for socioeconomic status).* *Provide clear definitions of the relevant terms used, how they were provided (by the participants/respondents, the researchers, or third parties), and the method(s) used to classify people into the different categories (e.g. self-report, census or administrative data, social media data, etc.)* *Please provide details about how you controlled for confounding variables in your analyses.*
Population characteristics	*Describe the covariate-relevant population characteristics of the human research participants (e.g. age, genotypic information, past and current diagnosis and treatment categories). If you filled out the behavioural & social sciences study design questions and have nothing to add here, write "See above."*
Recruitment	*Describe how participants were recruited. Outline any potential self-selection bias or other biases that may be present and how these are likely to impact results.*
Ethics oversight	*Identify the organization(s) that approved the study protocol.*

Note that full information on the approval of the study protocol must also be provided in the manuscript.

Field-specific reporting

Please select the one below that is the best fit for your research. If you are not sure, read the appropriate sections before making your selection.

☒ Life sciences ☐ Behavioural & social sciences ☐ Ecological, evolutionary & environmental sciences

For a reference copy of the document with all sections, see nature.com/documents/nr-reporting-summary-flat.pdf

Life sciences study design

All studies must disclose on these points even when the disclosure is negative.

Sample size	No sample-size calculations were performed. Sample sizes were chosen based on previous comparable mouse biodistribution and in vivo delivery studies, preliminary experiments, expected effect sizes, and the need to minimize animal use. For biodistribution experiments, three mice per group were used to assess robust organ-level distribution patterns. Larger sample sizes were used where greater biological variability or downstream endpoint analyses were expected, such as proteomics studies. Exact sample sizes and statistical tests are provided in the corresponding figure legends.
Data exclusions	No data were excluded.
Replication	All experiments were replicated three to four times independently.
Randomization	Mice were randomized in different groups before being assayed.
Blinding	Blinding was not relevant to this study because no bias could be made by the subject or the tester in the experiments performed.

Reporting for specific materials, systems and methods

We require information from authors about some types of materials, experimental systems and methods used in many studies. Here, indicate whether each material, system or method listed is relevant to your study. If you are not sure if a list item applies to your research, read the appropriate section before selecting a response.

Materials & experimental systems

n/a	Involved in the study	
☒	☐	Antibodies
☐	☒	Eukaryotic cell lines
☒	☐	Palaeontology and archaeology
☐	☒	Animals and other organisms
☒	☐	Clinical data
☒	☐	Dual use research of concern
☒	☐	Plants

Methods

n/a	Involved in the study	
☒	☐	ChIP-seq
☐	☒	Flow cytometry
☒	☐	MRI-based neuroimaging

Eukaryotic cell lines

Policy information about cell lines and Sex and Gender in Research

Cell line source(s)	MDA-MB-231 and HEK293T were obtained from the American Type Culture Collection.
Authentication	Cell lines were not independently authenticated by our laboratory; both lines were obtained directly from ATCC, which performs STR profiling on its reference stocks prior to distribution.
Mycoplasma contamination	All cell lines tested negative for mycoplasma contamination.
Commonly misidentified lines (See ICLAC register)	None of the cell lines used (MDA-MB-231, HEK293T) are listed on the ICLAC register of commonly misidentified cell lines.

Animals and other research organisms

Policy information about studies involving animals; ARRIVE guidelines recommended for reporting animal research, and Sex and Gender in Research

Laboratory animals	6–12 weeks old C57BL/6J mice and B6.Cg-Gt(ROSA)26Sortm14(CAG-tdTomato)Hze/J mice (also known as Ai14 mice) with mixed genders were sourced from the Bioresources Facility from Peter Doherty Institute (Victoria, Australia), Water and Eliza Hall Institute of Medical Research (Victoria, Australia), and Australian Bio Resources (New South Wales, Australia). C57BL/6J mice (mixed gender; 6–12 weeks old) were used for biodistribution studies, elemental analysis, plasma isolation for subsequent proteomics analysis, and safety and biocompatibility assays. Ai14 mice (mixed gender; 6–12 weeks old) were used for tdTomato expression studies.
Wild animals	This study did not involve wild animals.
Reporting on sex	In vivo biodistribution data were generated using mice with mixed genders. Sex-disaggregated analysis was not performed because sex was not the primary biological variable under investigation, and the study was not designed to assess sex-based differences. No gender-related data were collected.
Field-collected samples	This study did not involve samples collected from the field.
Ethics oversight	This work was conducted in accordance with the Australian code for the care and use of animals for scientific purposes, and experiments were approved by the University of Melbourne Animal Ethics Committee (Ethics 10404, 31339, and 27608).

Note that full information on the approval of the study protocol must also be provided in the manuscript.

Plants

Seed stocks	*Report on the source of all seed stocks or other plant material used. If applicable, state the seed stock centre and catalogue number. If plant specimens were collected from the field, describe the collection location, date and sampling procedures.*
Novel plant genotypes	*Describe the methods by which all novel plant genotypes were produced. This includes those generated by transgenic approaches, gene editing, chemical/radiation-based mutagenesis and hybridization. For transgenic lines, describe the transformation method, the number of independent lines analyzed and the generation upon which experiments were performed. For gene-edited lines, describe the editor used, the endogenous sequence targeted for editing, the targeting guide RNA sequence (if applicable) and how the editor was applied.*
Authentication	*Describe any authentication procedures for each seed stock used or novel genotype generated. Describe any experiments used to assess the effect of a mutation and, where applicable, how potential secondary effects (e.g. second site T-DNA insertions, mosiacism, off-target gene editing) were examined.*

Flow Cytometry

Plots

Confirm that:

- [x] The axis labels state the marker and fluorochrome used (e.g. CD4-FITC).
- [x] The axis scales are clearly visible. Include numbers along axes only for bottom left plot of group (a 'group' is an analysis of identical markers).
- [ ] All plots are contour plots with outliers or pseudocolor plots.
- [x] A numerical value for number of cells or percentage (with statistics) is provided.

Methodology

Sample preparation	Sample preparation procedures are described in the Methods section.
Instrument	CytoFLEX Nano (Beckman Coulter, USA) was used for nanoparticle flow cytometry.
Software	Data collection: CytExpert Nano<br>Data analysis: FlowJo V10
Cell population abundance	Up to 100% (nanoparticle suspensions).
Gating strategy	For nanoparticle analysis, background noise and debris were excluded based on FSC-H vs SSC-H. Events exceeding the fluorescence intensity threshold defined from uncoated nanoparticle controls were considered "positive".<br><br>Note: The CONTOUR PLOT plot box was not checked because these experiments used nanoparticle flow cytometry on acellular nanoparticle suspensions, rather than conventional cell-based flow cytometry. Therefore, cell population contour plots are not directly applicable.

- [x] Tick this box to confirm that a figure exemplifying the gating strategy is provided in the Supplementary Information.
