# Supplementary material for: Selective organ deposition of nanoparticles directed by metal–organic network coatings
Source: Nat Commun. 2026 Sep 2;17:10440. doi: 10.1038/s41467-026-77181-0 (PMC13631259; doi:10.1038/s41467-026-77181-0)
Supplement: Supplementary file 3 [file 41467_2026_77181_MOESM3_ESM.pdf]

nature portfolio

Peer Review File

Selective organ deposition of nanoparticles directed by metal–organic network coatings

Corresponding Author: Professor Frank Caruso

This manuscript has been previously reviewed at another journal. This document only contains information relating to versions considered at Nature Communications.

This file contains all reviewer reports in order by version, followed by all author rebuttals in order by version.

Version 0:

Reviewer comments:

Reviewer #1

(Remarks to the Author)
The authors have addressed most of my comments, especially my main comment was adressed by the authors.

Concern/Question 3: could the authors give a theoretical density of PEG (PEG per nm2) e.g. by calculations?

Concern/Question 4: I agree that DLS is a great method, especially when we think that these nanocarriers are in aqueous media. But: why can't the authors measure (at least some) samples in TEM and show the size increase?

Concerning my concern 5. ("Fig 3e..."): this was referring to the TEM picture (which is actually Fig 3a, please excuse my typo). The CytoFlex data is totally fine and expected as pointed out by the authors.

I would recommend to publish the paper after minor revisions.

Reviewer #2

(Remarks to the Author)
I am herein completing an additional review of the manuscript "Selective organ deposition of nanoparticles directed by metal–organic network coatings" that was initially peer reviewed at Nature Nanotechnology, and now a suitably revised version of the manuscript is under consideration at Nature Communications as a transferred manuscript.

I agree with the Editors that the scope and impact of this manuscript is suitable for Nature Communications.

I read the revised manuscript, checked the Response to Reviewer file, the new text, and the added Figures. In my view, these new data and conclusions address the prior reviewer concerns. Although Reviewer #2 raised concerns about novelty, clinical impact, and MPN NP characterization, I do not think that these concerns are relevant for potential publication, including at Nature Communications. The rebuttal letter provided suitable evidence for the claims made in this manuscript. I think that the approaches used are appropriate and sufficient to support the conclusions, and that the points raised by Reviewer #2 have been adequately addressed.

In short, the authors have addressed prior concerns well, and I recommend to accept the paper for publication in Nature Communications.

Version 1:

Reviewer comments:

Reviewer #1

(Remarks to the Author)
The authors have adressed my remarks in full. Publish the manuscript as is.

Open Access This Peer Review File is licensed under a Creative Commons Attribution 4.0 International License, which permits use, sharing, adaptation, distribution and reproduction in any medium or format, as long as you give appropriate credit to the original author(s) and the source, provide a link to the Creative Commons license, and indicate if changes were made.
In cases where reviewers are anonymous, credit should be given to 'Anonymous Referee' and the source.
The images or other third party material in this Peer Review File are included in the article's Creative Commons license, unless indicated otherwise in a credit line to the material. If material is not included in the article's Creative Commons license and your intended use is not permitted by statutory regulation or exceeds the permitted use, you will need to obtain permission directly from the copyright holder.
To view a copy of this license, visit https://creativecommons.org/licenses/by/4.0/
